# Model for Assessing Human Papillomavirus Vaccination Strategies

**DOI:** 10.3201/eid1301.060438

**Published:** 2007-01

**Authors:** Elamin H. Elbasha, Erik J. Dasbach, Ralph P. Insinga

**Affiliations:** * Merck Research Laboratories, North Wales, Pennsylvania, USA

**Keywords:** human papillomavirus, vaccines, cost-effectiveness analysis, cervical intraepithelial neoplasia, uterine cervical neoplasms, condylomata acuminate, theoretical models, nonlinear dynamics, herd immunity, research

## Abstract

We present a transmission dynamic model that can assess the epidemiologic consequences and cost-effectiveness of alternative strategies of administering a prophylactic quadrivalent (types 6/11/16/18) human papillomavirus (HPV) vaccine in a setting of organized cervical cancer screening in the United States. Compared with current practice, vaccinating girls before the age of 12 years would reduce the incidence of genital warts (83%) and cervical cancer (78%) due to HPV 6/11/16/18. The incremental cost-effectiveness ratio (ICER) of augmenting this strategy with a temporary catch-up program for 12- to 24-year olds was US $4,666 per quality-adjusted life year (QALY) gained. Relative to other commonly accepted healthcare programs, vaccinating girls and women appears cost-effective. Including men and boys in the program was the most effective strategy, reducing the incidence of genital warts, cervical intraepithelial neoplasia, and cervical cancer by 97%, 91%, and 91%, respectively. The ICER of this strategy was $45,056 per QALY.

## Abstract 2

A prophylactic quadrivalent vaccine can cost-effectively reduce the incidence of cervical cancer, cervical intraepithelial neoplasia, and genital warts.

Human papillomavirus (HPV) causes cervical intraepithelial neoplasia (CIN); cervical, anal, penile, vaginal, vulvar, and head/neck cancers; anogenital warts; and recurrent respiratory papillomatoses, resulting in disease and death in both women and men (*1*). Cervical cancer incidence and deaths have substantially decreased in countries with organized cervical cancer screening programs (*2*). However, despite this success, cervical cancer is the second most common malignancy among women and a leading cause of cancer death worldwide, with an estimated 493,000 new cases and 274,000 deaths in 2002 (*3*).

In the United States, public health authorities recommend that girls and women 11–26 years of age be vaccinated with the newly licensed quadrivalent HPV vaccine, Gardasil (Merck & Co., Inc., Whitehouse Station, NJ, USA), to prevent cervical cancer, precancerous and low-grade lesions, and genital warts caused by HPV types 6, 11, 16, or 18. Policymakers will need information on the epidemiologic and economic impact of HPV vaccination to formulate guidelines (*4**,**5*). Cohort models provided some of this information but could not fully assess the impact of HPV vaccination (*6*). In particular, vaccination will not only directly protect through vaccine-derived immunity but also indirectly through herd immunity. To account for these direct and indirect effects, a population dynamic model is necessary (*7*). Moreover, a dynamic model can evaluate a broader range of vaccination strategies (e.g., vaccination of boys and men). A few dynamic models exists (*6**,**8*), but only 1 has examined the cost-effectiveness of bivalent HPV (16/18) vaccination strategies (*9*).

We developed a dynamic model to assess the epidemiologic consequences and cost-effectiveness of alternative quadrivalent HPV (6/11/16/18) vaccination strategies. A Supplementary Appendix describes in detail the model structure and inputs. Specifically, we examined 2 questions: What is the potential impact of a quadrivalent HPV vaccine on HPV infection and disease in the US population? What is the cost-effectiveness of a quadrivalent HPV vaccine program when added to the current standard of care from the perspective of the US healthcare system?

## Methods

### Screening and Vaccination Strategies

We assumed that the vaccine will be combined with current screening and HPV disease treatment practices. We defined the reference vaccination strategy to be routine HPV vaccination of girls by age 12 (F12-only) (*10*). We also examined the following strategies: 1) routine vaccination of girls and boys by age 12 (F&M12), 2) routine vaccination of girls by age 12 and catch-up female vaccination for those ages 12–24 (F12-only+CUF-only), 3) routine vaccination of boys and girls by age 12 years and catch-up female vaccination for those ages 12–24 years (F&M12+CUF-only), and 4) routine vaccination of boys and girls by age 12 and catch-up female and male vaccination for those ages 12–24 (F&M12+CUF&M).

### Dynamic Model Structure

Our dynamic model has demographic and epidemiologic components (*11*, Supplementary Appendix). The demographic model defines the demographic characteristics of the population being simulated and describes how persons enter, age, and exit various categories. The heterosexually mixing population is divided into 17 age groups. Each age group consists of persons with low, medium, or high sexual activity.

Twelve-year-old persons enter the population at a gender-specific and sexual activity–specific rate. Persons then move between successive age groups at an age- and gender-specific rate per year (*11*). Persons exit the model upon death at an age- and gender-specific per capita death rate per year. Cervical cancer patients have an additional age- and stage-dependent death rate. Patients with CIN or genital warts do not face an additional risk for death.

**Figure 1:**
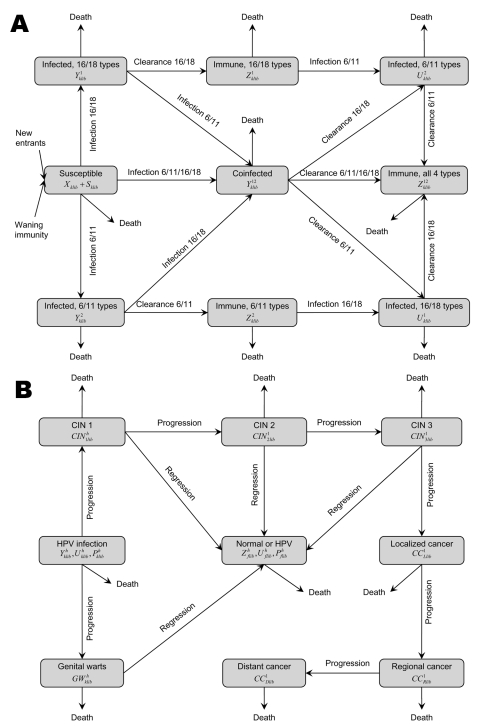
A simplified schematic diagram of human papillomavirus (HPV) infection and disease state transitions, lifetime duration of infection-derived immunity, unvaccinated compartments. A) Persons enter into the susceptible (*X*) compartment and leave all compartments at sex- and age-specific rate. A susceptible host may be infected by either or both HPV types. A host infected with a given type can also be infected with the other type and move into compartment (*Y*^12^). An infected person can clear infection with 1 type and can become immune to that type (*Z^h^*) and get infected with the other type (*U^h^*). Infection with and clearance of all types results in lifetime immunity. B) Cervical intraepithelial neoplasia (CIN) develops in females and progresses though several histologic states: infected with a normal cervix; CIN 1; CIN 2; CIN 3; localized, regional, and distant cervical cancer. CIN can regress to normal with or without infection. Genital warts can develop and clear in those infected with HPV 6/11.

The epidemiologic model simulates HPV transmission and the occurrence of CIN, cervical cancer, and external genital warts in this age-structured population. The acquisition of infection and progression of persons from infection to disease follow a similar natural history structure, as assumed in previous models for HPV 16/18 (*6*). We also incorporated HPV 6/11 infection and genital warts, and grouped infections into HPV 16/18, HPV 6/11, or HPV 6/11/16/18. We divided the population into distinct epidemiologic categories, according to the person’s status with respect to infection, disease, screening, and treatment (Supplementary Appendix, Figure 1A–B).

### Parameters for Estimates and Sources

A comprehensive search of the literature was conducted to obtain baseline values for the parameters of the model (Appendix^1^, Tables 1, 2, and 3). We used age-stratified data to estimate cytology screening rates (*12**–**14*). Estimates of cytology screening sensitivities and specificities were based on published studies (*15**,**16*).

The degree of protection from the vaccine (the proportion of challenges against which a recipient is protected) against incident infection (HPV 6/11 or 16/18) was 90%; against associated disease the degree of protection was 100% (*17**,**18*). We assumed the duration of protection was lifelong for the reference case (*6*) and examined a 10-year duration in sensitivity analyses. We assumed the natural course of disease was unaltered following vaccine failure or loss of vaccine-induced immunity. Because Gardasil is a prophylactic vaccine, we did not include any therapeutic benefits to recipients already infected with the vaccine types. We assumed that up to 70% of 12-year-olds received a 3-dose vaccine (*6*). Coverage increased linearly from 0% up to 70% during the first 5 years of the program (e.g., 14% in year 1, 28% in year 2) and remained at 70% thereafter. Vaccine coverage for the catch-up program increased linearly from 0% up to 50% during the first 5 years (e.g., 10% in year 1, 28% of unvaccinated in year 2), and the program was eliminated after year 5.

We assumed the cost of the HPV vaccine for 3 doses and administration would be US $360 (range $300–$500), consistent with previous analyses (*6*). All costs were updated to 2005 US dollars. Costs and quality-adjusted life years (QALY) were discounted at 3%.

### Simulation Method

We assessed the epidemiologic impact and cost-effectiveness of each vaccination strategy over a planning horizon of 100 years. We solved the model for the prevaccination steady-state values of the variables and used them as initial values for the vaccination model. Next, we solved the model for the entire time path of the variables until the system approached a steady-state.

### Validation Analyses

We established the face validity of the model by consulting with experts on assumptions regarding the natural history of HPV infection and disease (*19*). The accompanying Supplementary Appendix allows for further critical review of the model assumptions and provides the mathematical equations necessary to reproduce the results (*19*,*20*). The predictive validity of the model was evaluated by comparing model results with epidemiologic data from unscreened and screened populations in the United States (*2**,**21**–**23*).

### Sensitivity Analyses

Because of the large number of equations and inputs, we used a smaller version of the model to determine the most influential inputs. Based on these results, 1-way sensitivity analyses using the full model were performed on vaccine parameters (duration, degree, coverage, cost, target age), quality-of-life weights, discounting, and duration of natural immunity. We also conducted a multivariate sensitivity analysis that examined a pessimistic scenario (i.e., duration of protection = 10 years; vaccine coverage = 50%; health utility for genital warts; CIN 1, 2, 3, and CIS = 0.97; degree of protection against infection = 75%; and degree of protection against HPV-related disease = 85%). We also examined the role of herd immunity.

## Results

### Model Validation

**Figure 2:**
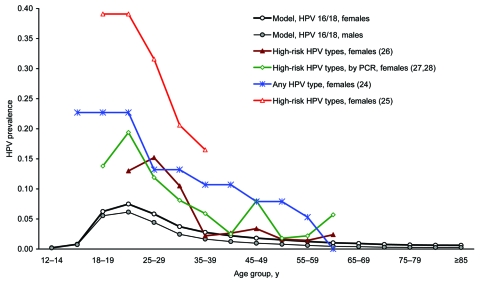
Human papillomavirus (HPV) prevalence by sex and age group, as predicted by the model and reported in selected studies from North America. HPV high risk includes types 16, 18, 31, 33, 35, 39, 45, 51, 52, 56, 58, 59, 68, 73, and 82.

Model predictions generally fell within the range of values reported in the literature. Overall, HPV 6/11 steady-state prevalence among females was 0.7%, which is similar to that reported by Giuliano et al. (*24*) for15- to 59-year-old women. The predicted age-specific HPV prevalence curve had a shape and magnitude at peak similar to data reported in the literature (*24*–*28*) (Figure 2). Without screening, the predicted HPV 16/18-attributable cervical cancer incidence curve had a shape and magnitude at peak (39 per 100,000 women-years for ages 45–50) similar to those estimated from unscreened US populations (*22*,*29*). The model predicted that 20% of all cervical cancer cases occurred among women who were never screened, similar to what has been observed in US populations (*30*). Also, the cervical cancer incidence curve (HPV 16/18 attributable) had a shape and magnitude at peak (8.3 per 100,000 women-years for ages 30−39 years) similar to that observed among recent cohorts of US women (*23*). However, the model predicted lower cervical cancer incidence among older cohorts. This approximation may be reasonable given that future cohorts of older women are expected to have lower cervical cancer incidence than women currently in older age groups (fewer women missed screening at younger ages among more recent cohorts [*13*,*14*]). Finally, with screening, the age-specific incidence curves for CIN and genital warts generally had shapes and magnitudes at peak similar to data reported in the literature (*21*,*31*).

### Epidemiologic Impact of HPV Vaccination Strategies (Reference Case)

Steady-state HPV prevalence rates were higher for boys or men than for girls or women across all age groups (Figure 2). Overall, HPV 16/18 steady-state prevalence among girls and women >12 years of age (2.4%) was higher than that for boys or men (1.7%) and increased with level of sexual activity (data not shown). For both sexes, prevalence increased with age, reached a peak in the 20- to 24-year age group and continuously declined thereafter.

**Figure 3:**
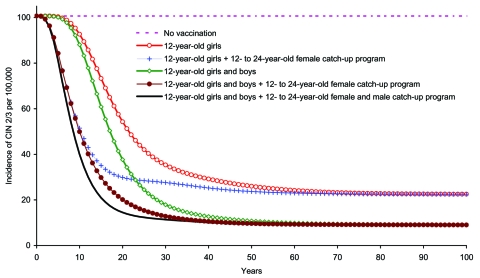
Incidence of cervical intraepithelial neoplasia (CIN) 2/3 due to human papillomavirus 6/11/16/18 infection among girls and women >12 years of age, by vaccination strategy.

**Figure 4:**
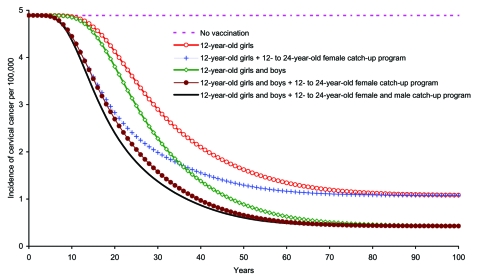
Incidence of cervical cancer due to human papillomavirus 16/18 infection among girls and women >12 years of age, by vaccination strategy.

Across all strategies, the effect of the vaccine was to steadily reduce CIN 2/3 incidence until the system approached a steady state (Figure 3). The largest reduction was accomplished by adopting F&M12+CUF&M. Cervical cancer curves shared the same qualitative features of those of CIN 2/3 (Figure 4). However, because cervical cancer progresses slowly, the effect of vaccination on the reduction in incidence and cancer deaths was more gradual compared with that for CIN 2/3 (Figures 3 and 4).

**Figure 5:**
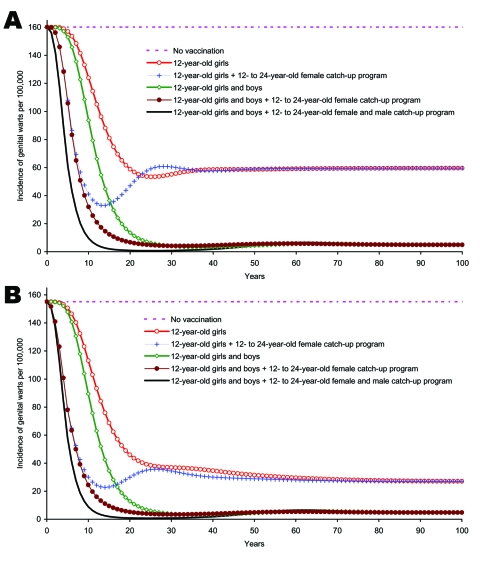
A) Incidence of genital warts due to human papillomavirus (HPV) 6/11 infection among boys and men >12 years of age by strategy. B) Incidence of genital warts due to HPV 6/11 infection among girls and women >12 years, of age by strategy.

For genital warts, the reduction occurred sooner (Figure 5A and 5B). Female-only vaccination strategies were effective in reducing genital warts incidence among adolescent girls and women (Figure 5B) and were also effective in reducing the incidence of genital warts among males, but were not as effective as strategies that included male vaccination (Figure 5A).

F&M12+CUF&M had the most effect on the number of cases of genital warts, CIN, and cervical cancer. Compared with screening only, this strategy substantially reduced the long-run, overall number of genital warts (97%), CIN 2/3 (91%), and cervical cancer cases (91%) among adolescent girls and women.

### Economic Impact of HPV Vaccination Strategies (Reference Case)

F&M12 was less effective and more costly (dominated) than F12-only+CUF-only (Table 1). The incremental cost-effectiveness ratio (ICER) of F12-only+CUF-only was US $4,666/QALY, and the most effective strategy (F&M12+CUF&M) had an ICER of $45,056/QALY.

**Table 1:** Cost-effectiveness analysis of alternative HPV vaccination strategies*

	Discounted total		Incremental		
Strategy	Costs	QALY	Costs	QALY	$/QALY†
No vaccination	72,659,302	2,698,711	––	––	––
12-y-old girls	74,042,990	2,699,178	1,383,687	467	2,964
12-y-old girls and boys	78,707,825	2,699,327	4,664,835	149	Dominated
12-y-old girls plus 12- to 24-y-old females catch-up	74,815,667	2,699,343	–3,892,159	16	4,666
12-y-old girls and boys plus 12− to 24-y-old females catch-up	79,746,357	2,699,461	4,930,690	118	41,803
12-y-old girls and boys plus 12− to 24-y-old females and males catch-up	81,761,210	2,699,506	2,014,853	45	45,056

*Assumes cost of vaccination series is US $360 and duration of protection is lifelong. All costs are measured in 2005 US dollars, and costs and QALY are discounted at 3%. HPV, human papillomavirus; QALY, quality-adjusted life years. †Compared with the preceding nondominated strategy. Strategy A is dominated if there is another strategy, B, that is more effective and less costly than strategy A.

### Sensitivity Analyses

**Figure 6:**
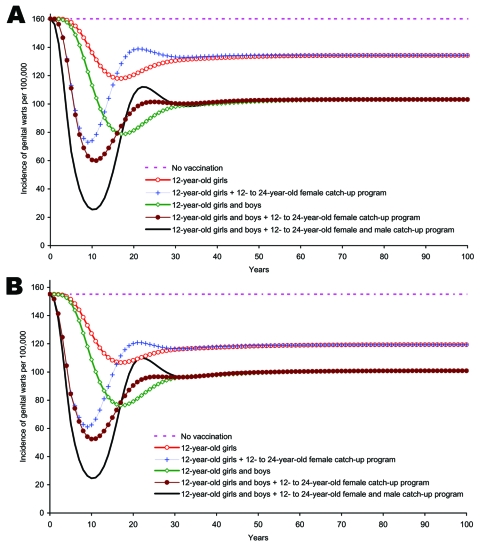
Sensitivity analysis. A) Incidence of genital warts due to human papillomavirus (HPV) 6/11 infection among boys and men >12 years of age, by strategy, 10 years’ duration of protection. B) Incidence of genital warts due to (HPV 6/11 infection among girls and women >12 years of age by strategy, 10 years’ duration of protection.

With 10 years’ duration of protection, vaccination reduced disease incidence steadily until ≈10–15 years after vaccination, when the loss of immunity among vaccinated persons and increased numbers of unvaccinated persons reversed these trends and caused the incidence to rise (Figures 6A and 6B). The rise in incidence continued until years 20–30, after which, it fell steadily until a steady state was approached. The timing and magnitude of the reduction and resurgence in incidence depended on the strategy. The largest reduction and lowest rebound were accomplished by using F&M12+CUF&M. If the duration of protection was only 10 years, long-term reductions in the annual number of cases of genital warts among males, CIN 2/3, and cervical cancer would be 36%, 25%, and 28%, respectively. In addition, ICERs increased by changing the duration of protection from lifelong to 10 years (Table 2).

**Table 2:** Sensitivity of incremental cost-effectiveness ratios (US $/QALY) of alternative HPV vaccination strategies to changes in inputs*

Input	F12 only	F12-only+ CUF only	F&M12+ CUF-only	F&M12+ CUF&M†
Baseline	2,964	4,666	41,803	45,056
Cost of vaccination series = $300	997	2,422	33,469	36,161
Cost of vaccination series = $500	7,553	9,900	61,250	65,810
Utility weights for CIN, CIS, GW = 0.97	5,241	7,739	82,700	83,714
Duration of protection = 10 y	Weakly dominated	21,121	54,755	54,928
Degree of protection against HPV 6/11/16/18 = 100%	2,094	4,187	Weakly dominated	51,436
Degree of protection against HPV 6/11/16/18 = 74%	4,273	5,403	39,990	43,930
Degree of protection against disease = 87%	3,116	4,922	40,269	43,974
Coverage with vaccination = 50%	2,636	4,221	23,862	36,235
Coverage with vaccination = 90%	3,449	5,269	Weakly dominated	100,418

*Unless specified otherwise, cost of vaccination series is US $360, and duration of protection is lifelong. QALY, quality-adjusted life years; HPV, human papillomavirus; F12-only, female vaccination by age 12; CUF, catch-up female vaccination for ages 12–24; F&M12+CUF only, female and male vaccination by age 12 and CUF; CIN, cervical intraepithelial neoplasia; CIS, carcinoma in situ; GW, genital warts. †Compared with the preceding nondominated strategy. Strategy A is dominated if there is another strategy, B, that is more effective and less costly than strategy A. The strategy of female and male vaccination by age 12 that did not include a catch-up program was dominated. A strategy is weakly dominated if there is another more effective program that has a lower incremental cost-effectiveness ratio.

**Figure 7:**
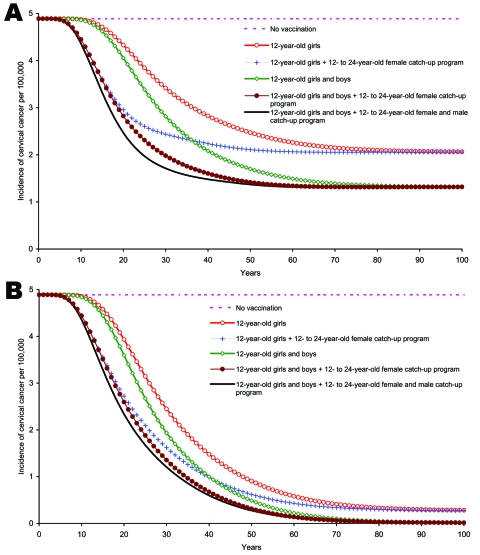
Sensitivity analysis. A) Incidence of cervical cancer due to human papillomavirus (HPV) 16/18 infection among girls and women >12 years of age with 50% coverage. B) Incidence of cervical cancer due to HPV 16/18 infection among girls and women >12 years of age with 90% coverage.

The long-term cervical cancer incidence and ICER were not very sensitive to changes in the degree of vaccine protection against infection and disease. However, the results were sensitive to varying vaccination coverage. For example, the impact of vaccination on cervical cancer was lower when coverage was 50% compared with 90% (Figure 7A and 7B). Lower coverage made vaccinating adolescent boys and men more cost-effective (Table 2). Increasing vaccination cost and quality of life weights increased ICERs.

Lower discount rates resulted in higher costs and QALY for each vaccination strategy. Discounting both costs and QALY at 1% decreased ICERs of the nondominated strategies: F12-only+CUF-only had an ICER of $448/QALY, whereas the ICER of F&M12+CUF&M was $28,614 /QALY. With a 5% discount rate, ICERs of these 2 strategies increased to $10,138/QALY and $64,413/QALY, respectively. HPV prevalence and burden of HPV-related diseases increased with shorter duration of natural immunity. A higher background rate of disease made the impact of vaccination look more favorable. For example, with 10-year duration of natural immunity, F12-only+CUF-only was cost-saving, whereas the ICER of F&M12+CUF&M was $11,567/QALY.

When the effects of herd immunity and benefits of prevention of HPV 6/11 were removed, the ICER of F12-only increased to $21,404. If 1 assumes a pessimistic scenario, the ICER of the F12-only+CUF-only strategy increased from $4,446/QALY to $29,053/QALY and the ICER of the F&M12+CUF&M increased from $45,056/QALY to $124,063/QALY.

**Figure 8:**
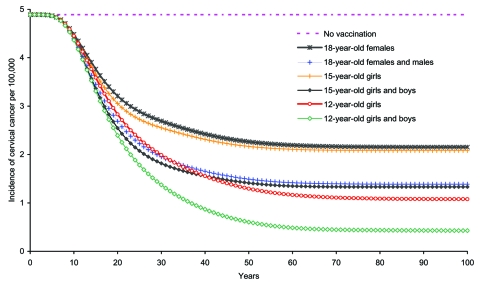
Impact of age that vaccination was begun on cervical cancer incidence due to human papillomavirus 16/18 infection among girls and women >12 years of age.

Because vaccination coverage rates are expected to be lower among older age groups, we assumed a rate of 50% among 15- and 18-year-olds. With these rates, F12-only+CUF-only had an ICER of $8,357/QALY compared with delaying age of vaccination to 18 years (Table 3). ICERs of vaccinating by age 12 years increased when coverage rates among persons of ages 15 and 18 years were higher. Increasing the target age of vaccination decreased the benefits of vaccination (Figure 8, Table 3).

**Table 3:** Incremental cost-effectiveness ratios of alternative HPV vaccination strategies with varying start age of vaccination*

Strategy	Discounted total		Incremental
	Costs	QALY	$/QALY†
18-y-old women plus 18- to 24-y-old female catch-up	73,553,847	2,699,192	1,858
15-y-old female adolescents plus 15- to 24-y-old female catch-up	73,895,046	2,699,214	Weakly dominated
12-y-old girls plus 12- to 24-y-old female catch-up	74,815,667	2,699,343	8,357
18-y-old women and men plus 18- to 24-y-old female and male catch-up	77,535,383	2,699,385	Weakly dominated
15-y-old female and male adolescents plus 15- to 24-y-old female and male catch-up	78,455,750	2,699,404	Weakly dominated
12-y-old female and male adolescents plus 12- to 24-y-old female and male catch-up program	81,761,210	2,699,506	42,697

*Assumes cost of vaccination series is US $360, duration of protection is lifelong, and coverage rate of 50% among age 15- to 24-y-olds. HPV, human papillomavirus; QALY, quality-adjusted life years. †Compared with the preceding nondominated strategy. Strategy A is dominated if there is another strategy, B, that is more effective and less costly than strategy A. A strategy is weakly dominated if there is another more effective program that has a incremental cost-effectiveness ratio.

Finally, to estimate the additional value of preventing HPV 6/11 infection, we conducted an analysis in which we assumed that persons had no protection against HPV 6/11 infection and related disease. The results of this analysis showed that ICERs of F12-only+CUF-only and F&M12+CUF&M increased to $11,254/QALY and $74,151/QALY, respectively.

## Discussion

We developed an integrated transmission dynamic model and economic evaluation to inform HPV vaccine policy recommendations and decisions. We gained valuable insights by comparing various vaccination strategies. In general, the results suggest that a quadrivalent HPV vaccine program that targets female adolescents and women, ages 12–24 years, can be cost-effective ($4,666/QALY) when compared with other commonly accepted medical interventions (*32*). These findings are consistent with other cohort-based cost-effectiveness analyses, which generally show that vaccination of 12-year-old girls can be cost-effective but also illustrate the substantial herd immunity benefits provided by vaccination.

Some results from this model were qualitatively similar to the results of other studies with respect to the finding that male vaccination was more attractive the lower the coverage among girls and women (*9*). However, the results of our base case differ qualitatively from that of Taira et al. (*9*) regarding the conclusion that vaccinating males and females would not be cost-effective. This difference in results may be explained as follows. First, unlike Taira et al., we accounted for the additional benefits conferred by protecting against HPV 6/11 infection among adolescent boys and girls, women, and men. Second, we were able to account for all the benefits and costs of vaccination realized by both those vaccinated and not vaccinated. Third, we assumed lower weights for the quality of life of women with CIN. However, the comparison is not perfect because our model tracks a population, whereas the model of Taira et al. follows a cohort. Hence, the composition of the numerators and denominators used in the ICERs differs between models. Finally, other methodologic differences occur between the 2 approaches that may explain the differences in results. For example, Taira et al. used steady-state values of HPV infection rates as inputs in their cost-effectiveness model, whereas we measured all outcomes over time, thereby capturing all the effects of transient dynamics generated from widespread vaccination. We also note that the results of the sensitivity analysis, when the effects of herd immunity and benefits of prevention of HPV 6/11 were removed, suggest that the ICER of the female vaccination strategy was $21,404/QALY, which is close to the value of $22,755/QALY reported in another study by Sanders and Taira (*33*).

An important finding from this analysis was that catch-up vaccination can substantially reduce disease in the short term. As a result, the female and male strategy that did not include a catch-up program was less effective and more costly.

One of the influential inputs was vaccine coverage. As female coverage rates decreased, male vaccination became more efficient. Another influential input in the analysis was the quality-of-life weights. The less HPV disease affected quality of life, the more the ICERs increased.

Duration of protection was also an influential parameter. Decreasing duration of vaccine protection to 10 years increased ICERs. However, the impact of this decrease may be mitigated by introducing a booster program. A reasonable approximation for how this program might fare would be to look at the sensitivity of ICERs to changes in vaccination cost. Thus, increasing the cost of the HPV vaccine series to $500 increased ICERs (Table 2). However, all nondominated (i.e., either are less costly or have lower ICERs than more effective strategies) female strategies remained cost-effective. Another influential parameter was the age vaccination was begun. Earlier vaccination resulted in greater benefits. F&M12+CUF&M was cost-effective ($42,697/QALY). However, vaccination by age 12 became less efficient, the higher the vaccination coverage was among older age groups.

Vaccination shifted the age of infection and disease to older age groups. For example, the age of peak cervical cancer incidence increased after introducing vaccination. The upward shifting of age of infection is a common feature of many vaccination programs (*11*).

We believe our modeling approach has several strengths. First, we did extensive validation with existing data. The model is also flexible enough to incorporate better data as they become available. Second, this model accounts for actual screening practices in the United States. Third, because output from this model is population based, the comparison with national registry data is better aligned than comparison of cohort model output with population data (*6*). Finally, all equations and inputs for this model are available to facilitate replication of findings and independent review of the model.

Several enhancements and extensions are desired. First, more relevant data on the natural history of type-specific HPV infection and disease (e.g., HPV transmission probability per sexual contact) are needed. Also, given the influence utility weights have on ICERs, more studies are needed to collect health utilities data on HPV disease states.

Second, we modeled only 4 HPV types and their associated diseases and assumed that HPV types have independent natural histories with no interaction among them. If cross-immunity exists between HPV types, a vaccine that reduces the prevalence of 1 type may promote the prevalence of other types through a process of competitive release. If, however, current or prior infection with 1 HPV type facilitates concurrent or subsequent infection with another HPV type, or if the vaccine provides cross-protection against other types, HPV vaccination could have the additional benefit of reducing the prevalence of HPV infection of types not covered by the vaccine (*34*). The evidence on interaction among HPV types to date is mixed and inconclusive (*35*–*39*).

Third, we modeled neither coinfection after disease developed in a person nor the coexistence of CIN lesions due to multiple HPV types in the cervix. By accounting for all the cost of vaccinating persons with undetected disease and no benefits for them as a result of the protection against the type that did not cause the disease, our results are biased against the catch-up program.

Fourth, the model assumed that all persons have equal access to healthcare, be it vaccination, screening, or treatment. However, this assumption may not be realistic and may overestimate the benefits of vaccination if women who have limited access to screening are also less likely to get vaccinated. Further studies are required to determine whether those who do not get vaccinated are also likely not to get screened.

Fifth, the current version of the model focused on heterosexual transmission of HPV and did not incorporate transmission between homosexual and heterosexual persons. Sixth, the scope of the model has been limited to cervical diseases and genital warts. HPV infection has also been associated with recurrent respiratory papillomatoses and cancers of the anus, penis, vagina, vulva, and head and neck. As evidence becomes available, the scope of the model will be broadened to incorporate the potential effects of vaccination on these other HPV conditions. Including these diseases in the model would render more favorable ICERs for vaccination.

Seventh, we did not include death and productivity costs (lost wages), as was done in other analyses (*40*). Including these costs would further reduce ICERs.

Finally, we did not consider vaccination strategies that include infants or mid-adults because current data available on vaccine safety and efficacy are limited to ages 9–26 years (*18*). As data for these other age groups become available, the model can examine these strategies.

In summary, the results from this model suggest that in a setting of organized cervical cancer screening, a prophylactic quadrivalent HPV (16/18/6/11) vaccine can 1) substantially reduce genital warts, CIN, and cervical cancer, 2) improve quality of life and survival, 3) be cost-effective (across a reasonably wide range of assumptions) when administered to girls before age 12 years (with or without a catch-up program), and 4) have a cost-effectiveness ratio near or below (depending on the underlying assumptions of the model) that of several other recommended vaccines, when implemented as a strategy that combines vaccination of both girls and boys before age 12 with a 12–24 years of age catch-up program.

## Supplementary Material

Appendix^1^ Demographic Model

Supplementary Appendix A Technical Report Accompanying Manuscript
