# Supplementary material for: Model for Assessing Human Papillomavirus Vaccination Strategies
Source: Emerg Infect Dis. 2007 Jan;13(1):28–41. doi: 10.3201/eid1301.060438 (PMC2725801; doi:10.3201/eid1301.060438)
Supplement: Appendix1 — Demographic Model [file 06-0438_appR-s1.pdf]

Publisher: CDC; Journal: Emerging Infectious Diseases
Article Type: Research; Volume: 13; Issue: 1; Year: 2007; Article ID: 06-0438
DOI: 10.3201/eid1301.060438; TOC Head: Research

Appendix[1]

[1]Refer to the Appendix References, below, for citations in this Appendix.

Demographic Model

The demographic model stratifies the population by gender and 17 age groups (12–14, 15–17, 18–19, 20–24, 25–29, 30–34, 35–39, 40–44, 45–49, 50–54, 55–59, 60–64, 65–69, 70–74, 75–79, 80–84, and ≥85 years). This age grouping permits age-specific inputs for patterns of sexual activity and cervical cancer screening and allows for age-specific outputs such as rates of cervical human papillomavirus (HPV) disease among girls and women, and genital warts among both males and females. Similar age groupings have been used by other sexually transmitted disease models (*1,2*). We further stratified each age group into 3 sexual activity groups (high, medium, low). We defined sexual activity according to the rates of sex partner change per year: low (0–1 per year), medium (2–4 per year), and high (≥5 per year). The number and the initial distribution of new entrants into the population by each gender were chosen to satisfy the Lotka characteristic equation with zero population growth (*3*). This allowed for variation in results across strategies to primarily be due to epidemiologic and program model features and not to changes in the demographic characteristics of the population over time (*3*).

The model starts with 12-year-olds entering the population at a gender-specific and sexual activity–specific rate, and transfers persons between successive age groups at an age- and gender-specific rate per year. The transfer rate depends on the rate of population growth, age- and gender-specific per capita mortality rate, and the number of years within an age group (*3*). We assumed equilibrium in the age distribution with zero population growth.

We set the population size in the model to 100,000 persons divided equally between females and males. Death rates for males and for females without cervical cancer were obtained from Vital Statistics data on gender- and age-specific mortality rates across all races for 2002 (*4*). Death rates among adolescent girls and women with cervical cancer were obtained from Surveillance Epidemiology and End Results (SEER)

Program data for 1997–2002 (*5*). Other demographic data were obtained from US Vital Statistics and the 2000 Census (*4,6*).

Epidemiologic Model

The epidemiologic model simulates HPV infection and occurrence of HPV disease (cervical intraepithelial neoplasia [CIN], cervical cancer, and genital warts) in the population. The acquisition of infection and progression from infection to disease follow a similar natural history structure, as assumed in previous models for HPV 16 and 18 (*7*). Building on these previous models, we also incorporated HPV 6 and 11 infection and genital warts and modeled infection by using 3 groups of HPV types (HPV 16/18, HPV 6/11, or HPV 6/11/16/18).

To simulate the occurrence of CIN, genital warts, and cervical cancer among those infected with HPV, we divided the population into distinct epidemiologic categories, according to the population's susceptibility to infection or the population's status with respect to infection, disease, screening, and treatment. These categories were similar to what has previously been defined in other models (*7*). The following, along with Figure 1, describes the movement of the population through these categories.

HPV Infection: Acquisition and Transmission

The epidemiologic model begins with 12-year-olds entering into the susceptible category *X*. Susceptible persons acquire HPV infection with a given type (HPV 16/18 infected only, HPV 6/11 infected only, or HPV 6/11 and HPV 16/18 infected) at a rate dependent upon gender, sexual activity group, age, and time. The rate at which persons of a given gender, sexual activity group, and age class at a given time acquire infection with a certain type (per capita force of infection) depends on the number of sexual partnerships and how these persons form partnerships with persons of the opposite sex, the fraction of infected sex partners, and the transmission probability per partnership. The formation of sexual partnerships is governed by a conditional probability sexual mixing matrix. Each cell in the mixing matrix represents the probability of a person of a given gender, sexual activity group, and age class having a sexual activity group, age-class specific partner from the opposite gender. In generating the mixing matrix, we used 2 parameters to depict the degree of mixing between age and sexual activity groups. This strategy

allowed us to represent a wide range of mixing patterns in the matrix, from fully assortative (as for persons with like persons when parameter is zero) to proportionate (random partners when parameter is 1) mixing (*1,2,8,9*). The baseline parameter values for the rate of sexual partner change, stratified by gender, sexual activity, and age, were calculated by using data from the National Health and Social Life Survey (*10*) and methods outlined in Garnett and Anderson (*2*) (Appendix Table 1).

Once HPV transmission occurs, susceptible persons enter the category of infected persons, *Y*. Persons leave this category when the infectious period for HPV ends and enter the category of recovered persons with a fixed duration of immunity, *Z*. In the base case, we assumed that duration of natural immunity is lifelong. Unvaccinated infected persons clear infection at a type-specific per capita rate. Persons in the immune (*Z*) category who are susceptible to only 1 type can be infected with that type and move to another infected/immune category, *U*.

A fraction of susceptible persons are vaccinated and move into the vaccination category *V*. The movement of those vaccinated through the model is similar to the movement of those unvaccinated, shown in Figure 1A. The remaining fraction of persons who are not vaccinated remains in the susceptible category *X*. The vaccine-induced immunity of those in the vaccinated category may wane over time. As a result, persons can eventually move to the susceptible category *S* at an age- and gender-dependent rate. We assumed that when a person loses vaccine-derived immunity, he or she becomes susceptible to infection with any of the types. In the base case, the duration of vaccine-derived immunity is assumed to be lifelong. Vaccinated persons can also experience a breakthrough infection and enter the category of infectious persons, *W,* at a per capita rate that depends on the degree of protection offered by the vaccine. Vaccinated persons can recover from an HPV infection at an age- and gender-specific rate by a factor that is different from the recovery rate for unvaccinated infected persons. Vaccinated persons then move to a category with fixed duration of immunity, *Q*. Persons in this category who are susceptible to 1 type can be infected with that type and move to another vaccinated infected/immune category, *P*.

No epidemiologic studies have estimated the probability of HPV infection transmission per partnership and by type. We assumed that this probability is higher for transmission from males to females (0.8) than that for transmission from females to males (0.7) (*12–15*). Using data on participants in the placebo arm of Merck's HPV vaccine clinical trials, we estimated mean duration of HPV infection before progression to CIN, or regression, at 1.2 years for HPV 16/18 and 0.7 years for HPV 6/11 (R. Insinga, unpub. data).

CIN, Cervical Cancer, and Genital Warts

CIN develops in infected girls and women at a specified rate and moves to the HPV disease categories of the model (Figure 1B). Several categories represent the true histologic health status of a woman: CIN grade 1 (CIN 1), CIN grade 2 (CIN 2), CIN grade 3 (CIN 3), localized cervical cancer (LCC), regional cervical cancer (RCC), distant cervical cancer (DCC), and cervical cancer survivors who are free from cancer. Women with CIN and cancer were further classified into undetected, detected, or treated categories. Two additional absorbing categories are for women who are no longer at risk for cervical cancer (*16*). These include the following: 1) women who have had a benign hysterectomy for reasons other than cervical cancer (at an age-specific rate) and 2) women treated and cured for cervical cancer. Finally, infection with the low-risk type can result in genital warts in females and males and move to the genital warts category, *GW* (*17*). We assumed women with benign hysterectomies can be infected and are at risk for genital warts (*18*). Women and men recovering from genital warts move to category *Z*.

We assumed all progression and regression rates to HPV and cancer states to be independent of age (*19–23*). Annual transition rates from HPV infection to clinically detectable CIN were calculated from studies by Winer et al. (*17*) and Insinga (R. Insinga, unpub. data). Several published reports were also used to estimate annual rates of CIN regression and progression to cervical cancer (*24–31*) (Merck, unpub. data). Incidence and regression rates for genital warts were obtained from Winer et al. (*17*) (Appendix Table 2). Hysterectomy rates; cervical cancer screening coverage, sensitivity, and specificity; and treatment efficacy were derived from several published studies (*32–40*) (Appendix Table 3).

Economic Parameters

All model costs were updated to 2005 US dollars by using the medical care component of the Consumer Price Index (*41*). The direct medical costs for screening and treatment for CIN, genital warts, and cervical cancer were based on administrative claims data and other sources (*42–44*). We measured the cost of cytology screening per unit time as the product of the cost per test, the test compliance rate, the frequency of administering the test per unit time, and the size of the unidentified population that is eligible for screening. We estimated the cost of following up on false-positive results of the cytology test as a function of the specificities of the cytology test and colposcopy procedure and the costs of colposcopy and biopsy. The cost of the HPV vaccine for 3 doses was assumed to be $360, which was consistent with HPV vaccination costs used in previous cost-effectiveness analyses (*7*). Productivity losses as a result of HPV disease or death were not included in the analyses (*45*).

Quality adjusted life years (QALYs) were measured by weighting survival time by the quality-of-life adjustment weights associated with each health state and integrating the sum of adjusted time in all these health states over the planning horizon. We measured survival time as the total number of years spent alive by the active population during a given period. The health utility values used to estimate QALYs were derived from various sources (*46–48*). Health utility values for diagnosed invasive cancer states were estimated by Myers et al. (*47*) at 0.76 for localized cancer and 0.67 for regional cancer; these values were derived from Gold et al. at 0.48 for distant cancer (*46*). We assumed that the quality of life for cervical cancer survivors after successful treatment would continue to be lower (0.76) than that of healthy women (*49,50*). Diagnosed and treated CIN 1 and CIN 2/3 states were assumed to have quality weights of 0.91 and 0.87, respectively (*47,48*). We assumed the quality weight for genital warts to be 0.91 (*47*) (Appendix Table 4).

Undiagnosed and asymptomatic HPV, CIN, and cancer states and successfully treated CIN states were assumed to have a quality-of-life weight similar to those of persons without these conditions. Gender- and *46*age-specific quality weights for non-HPV disease states were also derived from Gold et al. (). Time in these states was

multiplied by the age- and gender-specific weights to reflect the variation of quality of life by age and gender groups. We assumed that quality of life did not vary by sexual activity groups. Finally, all costs and effects were discounted to present value at a rate of 3%.

Appendix References

1. Garnett GP, Anderson RM. Factors controlling the spread of HIV in heterosexual communities in developing countries: patterns of mixing between age and sexual activity classes. Philos Trans R Soc Lond B Biol Sci. 1993;342:137–59.

2. Garnett GP, Anderson RM. Balancing sexual partnerships in age and activity stratified model of HIV transmission in heterosexual populations. IMA J Math Appl Med Biol. 1994;11:161–92.

3. Hethcote H. The mathematics of infectious diseases. SIAM Review. 2000;42:599–653.

4. Kochanek KD, Murphy SL, Anderson RN, Scott C. Deaths: final data for 2002. Natl Vital Stat Rep 53 (5). Hyattsville (MD): National Center for Health Statistics; 2004.

5. Surveillance, Epidemiology, and End Results (SEER) program. Public-use data (1973–2002), National Cancer Institute, DCCPS, Surveillance Research Program, Cancer Statistics Branch. Released 2005 Apr, based on the November 2004 submission. (cited 2006 Mar 13). Available from http://www.seer.cancer.gov

6. US Census Bureau. Census 2000 summary file 1. Washington: US Census Bureau; 2002.

7. Dasbach EJ, Elbasha EH, Insinga RP. Mathematical models for predicting the epidemiologic and economic impact of vaccination against human papillomavirus infection and disease. Epidemiol Rev. 2006;28:88–100.

8. Garnett GP, Hughes JP, Anderson RM, Stoner BP, Aral SO, Whittington WL, et al. The determination of the sexual mixing pattern of patients attending STD and other clinics in Seattle, USA, by contact tracing. Sex Transm Dis. 1996;23:248–57.

9. Garnett GP, Anderson RM. Contact tracing and the estimation of sexual mixing patterns: the epidemiology of gonococcal infections. Sex Transm Dis. 1993;20:181–91.

10. Lauman E, Gagnon J, Michael R, Michaels S. The social organization of sexuality. Chicago: University of Chicago Press; 1994.

11. Abma JC, Sonenstein FL. Sexual activity and contraceptive practices among teenagers in the United States, 1988 and 1995. National Center for Health Statistics. Vital Health Stat. 2001;23:1–79.

12. Hughes JP, Garnett GP, Koutsky L. The theoretical population level impact of a prophylactic human papillomavirus vaccine. Epidemiology. 2002;13:631–9.

13. Oriel JD. Natural history of genital warts. Br J Vener Dis. 1971;47:1–13.

14. Frega A, Stentella P, Villani C, Ruzza D, Marcomin G, Rota F, et al. Correlation between cervical intraepithelial neoplasia and human papillomavirus male infections: a longitudinal study. Eur J Gynaecol Oncol. 1999;20:228–30.

15. Burchell AN, Richardson H, Mahmud SM, Trottier H, Tellier PP, Hanley J, et al. Modeling the sexual transmissibility of human papillomavirus infection using stochastic computer simulation and empirical data from a cohort study of young women in Montreal, Canada. Am J Epidemiol. 2006;163:534–43.

16. Myers ER, McCrory DC, Nanda K, Bastian L, Matchar DB. Mathematical model for the natural history of human papillomavirus infection and cervical carcinogenesis. Am J Epidemiol. 2000;151:1158–71.

17. Winer RL, Kiviat NB, Hughes JP, Adam DE, Lee S-K, Kuypers JM, et al. Development and duration of human papillomavirus lesions, after initial infection. J Infect Dis. 2005;191:731–8.

18. Castle PE, Schiffman M, Bratti MC, Hildesheim A, Herrero R, Hutchinson ML, et al. A population-based study of vaginal human papillomavirus infection in hysterectomized women. J Infect Dis. 2004;190:458–67.

19. Castle PE, Schiffman M, Herrero R, Hildesheim A, Rodriguez AC, Bratti MC, et al. A prospective study of age trends in cervical human papillomavirus acquisition and persistence in Guanacaste, Costa Rica. J Infect Dis. 2005;191:1808–16.

20. Syrjanen S, Shabalova I, Petrovichev N, Podistov J, Ivanchenko O, Zakharenko S, et al. Age-specific incidence and clearance of high-risk human papillomavirus infections in women in the former Soviet Union. Int J STD AIDS. 2005;16:217–23.

21. Dalstein V, Riethmuller D, Pretet JL, Le Bail Carval K, Sautiere JL, Carbillet J-P, et al. Persistence and load of high-risk HPV are predictors for development of high-grade cervical lesions: a longitudinal French cohort study. Int J Cancer. 2003;106:396–403.

22. Molano M, Van den Brule A, Plummer M, Weiderpass E, Posso H, Arslan A, et al. Determinants of clearance of human papillomavirus infections in Colombian women with normal cytology: a population-based, 5-year follow-up study. Am J Epidemiol. 2003;158:486–94.

23. Winer RL, Koutsky LA. Human papillomavirus through the ages. J Infect Dis. 2005;191:1787–9.

24. Sanders GD, Taira AV. Cost-effectiveness of a potential vaccine for human papillomavirus. Emerg Infect Dis. 2003;9:37–48.

25. Goldie SJ, Grima D, Kohli M, Wright TC, Weinstein MC, Franco E. A comprehensive natural history model of human papillomavirus (HPV) infection and cervical cancer: potential impact of and HPV 16/18 Vaccine. Int J Cancer. 2003;106:896–904.

26. Kataja V, Syrjanen K, Mantyjarvi R, Vayrynen M, Syrjanen S, Saarikoski S, et al. Prospective follow-up of cervical HPV infections: life table analysis of histopathological, cytological and colposcopic data. Eur J Epidemiol. 1989;5:1–7.

27. De Aloysio D, Miliffi L, Iannicelli T, Penacchioni P, Bottiglioni F. Intramuscular interferon-beta treatment of cervical intraepithelial neoplasia II associated with human papillomavirus infection. Acta Obstet Gynecol Scand. 1994;73:420–4.

28. Westergaard L, Norgaard M. Severe cervical dysplasia: control by biopsies or primary conization? A comparative study. Acta Obstet Gynecol Scand. 1981;60:549–54.

29. Sastre-Garau X, Cartier I, Jourdan-Da Silva N, De Cremoux P, Lepage V, Charron D, et al. Regression of low-grade cervical intraepithelial neoplasia in patients with HLA-DRB1*13 genotype. Obstet Gynecol. 2004;104:751–5.

30. Matsumoto K, Yasugi T, Oki A, Fujii T, Nagata C, Sekiya S, et al. IgG antibodies to HPV16, 52, 58 and 6 L1-capsids and spontaneous regression of cervical intraepithelial neoplasia. Cancer Lett. 2006;231:309–13.

31. Berkhof J, de Bruijne MC, Zielinski GD, Meijer CJ. Natural history and screening model for high-risk human papillomavirus infection, neoplasia and cervical cancer in the Netherlands. Int J Cancer. 2005;115:268–75.

32. Keshavarz H, Hillis SD, Kieke BA, Marchbanks PA. Hysterectomy surveillance—United States, 1994–1999. MMWR CDC Surveill Summ. 2002;51:1–8. Available from http://www.cdc.gov/mmwr/preview/mmwrhtml/ss5105a1.htm

33. Insinga RP, Glass AG, Rush BB. Pap screening in a U.S. health plan. Cancer Epidemiol Biomarkers Prev. 2004;13:355–60.

34. Bigras G, de Marval F. The probability for a Pap test to be abnormal is directly proportional to HPV viral load: results from a Swiss study comparing HPV testing and liquid-based cytology to detect cervical cancer precursors in 13,842 women. Br J Cancer. 2005;93:575–81.

35. Coste J, Cochand-Priollet B, De Cremoux P, Le Gales C, Cartier I, Molinie V, et al. Cross sectional study of conventional cervical smear, monolayer cytology, and human papillomavirus DNA testing for cervical cancer screening. BMJ. 2003;326:733.

36. Mitchell MF, Schottenfeld D, Tortolero-Luna G, Cantor SB, Richards-Kortum R. Colposcopy for the diagnosis of squamous intraepithelial lesions: a meta-analysis. Obstet Gynecol. 1998;91:626–31.

37. Chesson HW, Blandford JM, Gift TL, Tao G, Irwin KL. The estimated direct medical cost of sexually transmitted diseases among American youth, 2000. Perspect Sex Reprod Health. 2004;36:11–9.

38. Flannelly G, Langhan H, Jandial L, Mana E, Campbell M, Kitchener H. A study of treatment failures following large loop excision of the transformation zone for the treatment of cervical intraepithelial neoplasia. Br J Obstet Gynaecol. 1997;104:718–22.

39. Ries LAG, Eisner MP, Kosary CL, Hankey BF, Miller BA, Clegg L, et al. SEER cancer statistics review, 1975–2002. Bethesda (MD): National Cancer Institute; 2005. (cited 2006 Mar 13). Available from http://seer.cancer.gov/csr/1975_2002/

40. Cruickshank ME, Sharp L, Chambers G, Smart L, Murray G. Persistent infection with human papillomavirus following the successful treatment of high-grade cervical intraepithelial neoplasia. BJOG. 2002;109:579–81.

41. US Bureau of Labor Statistics. Statistical abstracts of the United States: consumer price index. Washington: National Center for Health Statistics; 2002.

42. Insinga RP, Dasbach EJ, Myers ER. The health and economic burden of genital warts in a set of private U.S. health plans. Clin Infect Dis. 2003;36:1397–403.

43. Medstat. MarketScan® database. Ann Arbor (MI): Thomson Medstat; 2001.

44. Kim JJ, Wright T, Goldie S. Cost-effectiveness of alternative triage strategies for atypical squamous cells of undetermined significance. JAMA. 2002;287:2382–90.

45. Gold MR, Siegel JE, Russell LB, Weinstein MC, editors. Cost-effectiveness in health and medicine. Report of the Panel on Cost-Effectiveness in Health and Medicine. New York: Oxford University Press; 1996.

46. Gold M, Franks P, McCoy K, Fryback D. Toward consistency in cost-utilities analysis: using national measures to create condition-specific values. Med Care. 1998;36:778–92.

47. Myers E, Green S, Lipkus I. Patient preferences for health states related to HPV infection: visual analogue scales vs. time trade-off elicitation. Proceedings of the 21st International Papillomavirus Conference. Abstract no. 390.2. Mexico City, Mexico. 2004.

48. Insinga RP, Glass AG, Myers ER, Rush BB. Abnormal outcomes following cervical cancer screening: event duration and health utility loss. Med Decis Making. 2006. In press.

49. Andersen B. Stress and quality of life following cervical cancer. J Natl Cancer Inst. 1996;21:65–70.

50. Wenzel L, DeAlba I, Habbal R, Kluhsman BC, Fairclough D, Krebs L, et al. Quality of life in long-term cervical cancer survivors. Gynecol Oncol. 2005;97:310–7.

Appendix Table 1. Baseline behavioral parameter values for the sexually active population*

Activity group	Proportion of population, %		Relative partner acquisition rate
	Male	Female	
1 (highest)	2.56	2.56	11.29
2	11.47	11.47	2.96
3 (lowest)	85.97	85.97	1.0
Age group, y	Relative partner acquisition rate	Overall mean partner acquisition rate	
12–14	0.11	0.1	
15–17	1.18	0.3	
18–19	2.42	1.3	
20–24	2.61		
25–29	2.55		
30–34	1.72		
35–39	1.65		
40–44	1.53		
45–49	1.38		
50–54	1.25		
55–59	1.00		
60–69	0.61	0.5	
≥70	0.44		

*Sources: Lauman et al. (*10*), Abma and Sonenstein (*11*).

Appendix Table 2. Baseline biologic parameter values for HPV disease categories*

Parameter	Base-case estimate	Source†

Progression in the presence of HPV 16/18 per year, %		
Normal to CIN 1	9.4	(RI)
Normal to CIN 1 to CIN 2	5.8	(*17*,RI)
Normal to CIN 1 to CIN 2 to CIN 3	3.5	(*17*,RI)
CIN 1 to CIN 2	13.6	(MRK)
CIN 2 to CIN 3 (severe dysplasia)	14.0	(*26,27*)
CIN 3 - severe dysplasia to CIN 3 - CIS 1	42.0	(*26,28*)
CIS 1 to CIS 2	5.0	
CIS 2 to LCC	18.0	
LCC to RCC	10.0	(*16,24,25,31*)
RCC to DCC	30.0	(*16*)
Progression in the presence of HPV 6/11 per year, %		
Normal to CIN 1	9.5	(RI)
Normal to CIN 1 to CIN 2	1.9	(RI)
Normal to CIN 1 to CIN 2 to CIN 3	0.0	(RI)
CIN 1 to CIN 2	0.0	(MRK)
Normal to genital warts	57	(*17*)
Mean duration of acute HPV infection, y		
HPV 16/18 infection	1.2	(RI)
HPV 6/11 infection	0.7	(RI)
Regression of HPV 16/18+ disease per year, %		
CIN 1 to normal/HPV	32.9	(MRK,*29*)
CIN 2 to normal/HPV	21.0	(*26,27,30*)
CIN 2 to CIN 1	13.3	(*27*)
CIN 3 (severe dysplasia) to normal/HPV	11.0	(*26*)
CIN 3 (severe dysplasia) to CIN 1	3.0	(*26,27*)
CIN 3 (severe dysplasia) to CIN 2	3.0	(*26,27*)
Regression of HPV 6/11+ disease per year, %		
CIN 1 to normal/HPV	55.2	(MRK)
Genital warts to normal/HPV	87.5	(*17*)
Age (y) and stage-specific cervical cancer mortality rates per year, 1997–2002, %		(*5*)
For LCC		
15–29	0.7	
30–39	0.6	
40–49	0.8	
50–59	1.9	
60–69	4.2	
≥70	11.6	
For RCC		
15–29	13.4	
30–39	8.9	
40–49	11.0	
50–59	10.1	
60–69	17.6	
≥70	28.6	
For DCC		
15–29	42.9	
30–39	41.0	
40–49	46.7	
50–59	52.7	
60–69	54.6	
≥70	70.3	

*HPV, human papillomavirus; CIN, cervical intraepithelial neoplasia; CIS, carcinoma in situ; LCC, localized cervical cancer; RCC, regional cervical cancer; DCC, distant cervical cancer.
†RI, R. Insinga, unpub. data; MRK, Merck, unpub. data.

Appendix Table 3. Hysterectomy, screening, and treatment parameters*

Parameter	Base-case	Source

	estimate	
Hysterectomy rate, % per year		(*32*)
15–24 y	0.02	
25–29 y	0.26	
30–34 y	0.53	
35–39 y	0.89	
40–44 y	1.17	
45–54 y	0.99	
≥55 y	0.36	
Cervical cytology screening, excluding those with hysterectomy, % per year		(*33*)
10–14 y	0.6	
15–19 y	21.0	
20–24 y	44.8	
25–29 y	61.6	
30–34 y	54.9	
35–39 y	50.5	
40–44 y	48.1	
45–49 y	49.1	
50–54 y	51.1	
55–59 y	46.7	
60–64 y	42.5	
65–69 y	38.9	
70–74 y	29.6	
75–79 y	20.1	
80–84 y	11.1	
≥85	5.5	
Females never screened, %	5.0	
Liquid-based cytology specificity, %	94	(*34,35*)
Colposcopy sensitivity, %	96	(*36*)
Colposcopy specificity, %	48	(*36*)
GW patients seeking physician care, %	75	(*37*)
Symptom development, % per year		Assumed
Localized cervical cancer	4	
Regional cervical cancer	18	
Distant cervical cancer	90	
Eradication with treatment, %		
For CIN 1	96	(*38*)
For CIN 2	92	(*38*)
For CIN 3, CIS	92	(*38*)
For localized cervical cancer	92	(*39*)
For regional cervical cancer	55	(*39*)
For distant cervical cancer	17	(*39*)
Persistence of HPV after treatment for CIN or GW, %	34	(*40*)

*HPV, human papillomavirus; GW, genital warts; CIN, cervical intraepithelial neoplasia; CIS, carcinoma in situ.

Appendix Table 4. Cost and quality-of-life parameters*

Parameter	Base-case estimate	Source
Costs of diagnosing and treating HPV disease		*(42–44)*
Genital warts	$489	
Liquid-based cytology screening	$99	
Colposcopy and biopsy	$318	
CIN 1	$1,554	
CIN 2/3, CIS	$3,483	
Localized cervical cancer	$26,470	

Regional cervical cancer	$28,330		
Distant cervical cancer	$45,376		
Quality-of-life weights (0–1 scale)			
CIN 1	0.91		*(47)*
CIN 2/3, CIS	0.87		*(47)*
Localized cervical cancer	0.76		*(47)*
Regional cervical cancer	0.67		*(47)*
Distant cervical cancer	0.48		*(46)*
Cervical cancer survivor	0.84		*(47,49,50)*
Genital warts	0.91		*(47)*
No condition	F	M	*(46)*
12–17 y	0.93	0.93	
18–34 y	0.91	0.92	
35–44 y	0.89	0.90	
45–54 y	0.86	0.87	
55–64 y	0.80	0.81	
65–74 y	0.78	0.76	
≥75 y	0.70	0.69	

*HPV, human papillomavirus; CIN, cervical intraepithelial neoplasia; CIS, carcinoma in situ; F, females; M, males.
