# Supplementary material for: Model for Assessing Human Papillomavirus Vaccination Strategies
Source: Emerg Infect Dis. 2007 Jan;13(1):28–41. doi: 10.3201/eid1301.060438 (PMC2725801; doi:10.3201/eid1301.060438)
Supplement: Supplementary Appendix — A Technical Report Accompanying Manuscript [file 06-0438_SuppApp-s2.pdf]

This material, provided by the authors as a supplement to Model for Assessing Human Papillomavirus Vaccination Strategies, is not part of Emerging Infectious Diseases contents.

# Supplementary Online Appendix

## A Technical Report Accompanying Manuscript:

Elbasha EH, Dasbach EJ, Insinga RP. Model for assessing human papillomavirus vaccination strategies. Emerg Infect Dis. 2007;13.

Elamin H. Elbasha, PhD  Erik J. Dasbach, PhD

Ralph P. Insinga, PhD

Health Economic Statistics, UG1C-60
Merck Research Laboratories
PO Box 1000
North Wales, PA 19454-1099

November 21, 2006

This material, provided by the authors as a supplement to Model for Assessing Human Papillomavirus Vaccination Strategies, is not part of Emerging Infectious Diseases contents.

# Contents

**1 Introduction** **5**

**2 The demographic model** **6**
2.1 Demographic model structure . . . . . . . . . . . . . . . . . . . . . 6
2.2 Estimates of the demographic model parameters . . . . . . . . . 7

**3 The epidemiologic model** **11**
3.1 HPV transmission . . . . . . . . . . . . . . . . . . . . . . . . . . . 11
3.1.1 Susceptible individuals $X$ . . . . . . . . . . . . . . . . . . 11
3.1.2 Infected individuals $Y$ . . . . . . . . . . . . . . . . . . . . 13
3.1.3 Partially immune individuals $Z$ . . . . . . . . . . . . . . . 14
3.1.4 Infected individuals with partial immunity $U$ . . . . . . . 15
3.1.5 Vaccinated individuals $V$ . . . . . . . . . . . . . . . . . . 16
3.1.6 Vaccinated individuals with waned immunity $S$ . . . . . . 16
3.1.7 Infectious vaccinated individuals $W$ . . . . . . . . . . . . 16
3.1.8 Vaccinated, partially immune individuals $Q$ . . . . . . . . 17
3.1.9 Vaccinated, infected individuals with partial immunity $P$ 17
3.2 Cervical intraepithelial neoplasia . . . . . . . . . . . . . . . . . . 18
3.2.1 Undetected CIN $CIN_s$ . . . . . . . . . . . . . . . . . . . 18
3.2.2 Detected CIN $DCIN_s$ . . . . . . . . . . . . . . . . . . . 20
3.2.3 Treated CIN $TCIN_s$ . . . . . . . . . . . . . . . . . . . . 21
3.2.4 Treated CIN but infectious $ICIN_s$ . . . . . . . . . . . . 21
3.3 Cervical carcinoma in situ . . . . . . . . . . . . . . . . . . . . . . 21
3.3.1 Undetected CIS $CIS_s$ . . . . . . . . . . . . . . . . . . . 21
3.3.2 Detected CIS $DCIS_s$ . . . . . . . . . . . . . . . . . . . 22
3.3.3 Treated CIS $TCIS_s$ . . . . . . . . . . . . . . . . . . . . 22
3.3.4 Treated CIS but infectious $ICIS_s$ . . . . . . . . . . . . 22
3.4 Cervical cancer . . . . . . . . . . . . . . . . . . . . . . . . . . . . 23
3.4.1 Undetected cervical cancer $CC_s$ . . . . . . . . . . . . . . 23
3.4.2 Detected cervical cancer $DCC_s$ . . . . . . . . . . . . . . 24
3.4.3 Cervical cancer survivors $SCC$ . . . . . . . . . . . . . . 24
3.5 Genital warts $GW$ . . . . . . . . . . . . . . . . . . . . . . . . . . 24
3.6 Hysterectomies for benign conditions . . . . . . . . . . . . . . . . 25
3.6.1 Susceptible individuals $HX$ . . . . . . . . . . . . . . . . 25
3.6.2 Infected individuals $HY$ . . . . . . . . . . . . . . . . . . 26
3.6.3 Partially immune individuals $HZ$ . . . . . . . . . . . . . 26
3.6.4 Infected individuals with partial immunity $HU$ . . . . . . 27
3.6.5 Vaccinated individuals $HV$ . . . . . . . . . . . . . . . . . 27
3.6.6 Vaccinated individuals with waned immunity $HS$ . . . . . 27
3.6.7 Infectious vaccinated individuals $HW$ . . . . . . . . . . . 27
3.6.8 Vaccinated, partially immune individuals $HQ$ . . . . . . . 28
3.6.9 Vaccinated, infected individuals with partial immunity $HP$ 28
3.6.10 Genital warts $GW$ . . . . . . . . . . . . . . . . . . . . . 29
3.7 Forces of HPV infection $\lambda$ . . . . . . . . . . . . . . . . . . . . . 29

This material, provided by the authors as a supplement to Model for Assessing Human Papillomavirus Vaccination Strategies, is not part of Emerging Infectious Diseases contents.

3.8 Mixing preferences . . . . . 30
3.8.1 Mixing matrix $\rho$ . . . . . 30
3.8.2 Estimates of the mixing matrix . . . . . 31
3.9 Balancing population . . . . . 33
3.10 Estimates of epidemiologic parameters . . . . . 33
3.10.1 Estimates of natural history parameters . . . . . 33
3.10.2 Estimates of other clinical parameters . . . . . 36
3.10.3 Estimates of vaccine parameters . . . . . 36

**4 Epidemiologic impact of screening and vaccination strategies 36**
4.1 Years of life . . . . . 36
4.2 Quality-adjusted life years . . . . . 36
4.3 Estimates of quality of life weights . . . . . 38

**5 Economic consequences of screening and vaccination strategies 39**
5.1 Screening costs . . . . . 39
5.2 Treatment costs . . . . . 40
5.3 Vaccination costs . . . . . 41
5.4 Total costs . . . . . 41
5.5 Estimates of costs . . . . . 41
5.6 Cost-effectiveness ratio . . . . . 42

**6 Analysis using the model 42**
6.1 Simulations with the baseline estimates of the parameters . . . . 42
6.2 Model validation . . . . . 43

# List of Figures

1 Age distribution for 2000 US population 12 years & above and model (0% annual growth). . . . . 10
2 Age distribution for 2000 US population 12 years & above and model (1.23% annual growth). . . . . 11
3 Unvaccinated compartments of the HPV model . . . . . 12
4 Vaccinated compartments of the HPV model . . . . . 15
5 Cervical intraepithelial neoplasia model . . . . . 19
6 Cervical cancer (CC) model . . . . . 23

# List of Tables

1 Description of variables and subscripts . . . . . 8
2 Description of parameters . . . . . 9
3 Baseline behavioral parameter values for the sexually active population . . . . . 32
4 Baseline biological parameter values for the HPV and disease compartments and hysterectomy . . . . . 34

This material, provided by the authors as a supplement to Model for Assessing Human Papillomavirus Vaccination Strategies, is not part of Emerging Infectious Diseases contents.

5 Annual age-specific cervical cancer mortality rates, 1997–2002 . . 35
6 Cervical cytology screening and colposcopy characteristics and rates of cure and symptom recognition . . . . . . . . . . . . . . . 37
7 Quality of life weights . . . . . . . . . . . . . . . . . . . . . . . . 39
8 Cost of screening, diagnosis, and treatment . . . . . . . . . . . . 42

This material, provided by the authors as a supplement to Model for Assessing Human Papillomavirus Vaccination Strategies, is not part of Emerging Infectious Diseases contents.

# 1 Introduction

This technical report includes the mathematical model that was constructed to assess the impact of HPV vaccination strategies. It provides a detailed description of various model components as they relate to HPV infection, disease progression, vaccine characteristics, vaccination strategies, and the impact of HPV vaccination on epidemiologic and economic outcomes. The model allows for aggregating costs of vaccination, screening, and treatment of the population over time, compares them with total health outcomes as measured, for example, by quality adjusted life years (QALYs), and calculates incremental cost-effectiveness ratios for various vaccination strategies.

In constructing this model, we reviewed other relevant previous models and incorporated some of their structures and inputs. These included cervical cancer screening cohort models [17, 18, 21, 58, 61, 50], HPV vaccination cohort models [76, 71, 53, 31, 32], and HPV vaccination dynamic models [39, 28, 5, 19]. This model differs from its predecessors in several ways. First, the approach is more comprehensive in the sense that it incorporates the epidemiology of HPV infection, disease, and economics into a single dynamic model. Besides capturing the direct and indirect 'herd immunity' benefits and costs of vaccination for the population over time, the added advantage of this latter approach is its transparency, making critical review of the model and reproducibility of results [81] feasible without needing to review the actual source code used to generate the results. In particular, publication of the model includes the mathematical equations that summarize in their entirety the actual workings of the model. These equations can then be entered into any standard mathematical software package such as Mathematica® (Wolfram Research, Champaign, IL) or MatLab® (MathWorks, Natick, MA) to reproduce the results. Second, we also convened an expert panel that reviewed model assumptions and provided guidance on some aspects of the natural history of disease where there was little or no clinical evidence. Finally, key inputs in this model are based on data from recent studies that were not available when previous models were constructed.

For ease of exposition, the model is divided into two major components. The first part, which is presented in section 2, is a description of the demographic aspects of the model. This component of the model is intended to mimic the current age structure of the US population. Section 3 includes the second part which consists of the epidemiologic model that describes HPV transmission, and progression to cervical intraepithelial neoplasia (CIN), cervical cancer, and genital warts. Because females who undergo hysterectomies for benign conditions are no longer at risk of developing CIN and cervical cancer but can contribute to the transmission of HPV, another submodule for benign hysterectomy is created. Descriptions of the forces of infection, mixing preferences, and estimates of the epidemiologic model completes section 3. In sections 4 and 5, we describe how the epidemiologic and economic impact of screening and vaccination strategies are assessed.

This material, provided by the authors as a supplement to Model for Assessing Human Papillomavirus Vaccination Strategies, is not part of Emerging Infectious Diseases contents.

# 2 The demographic model

## 2.1 Demographic model structure

The demographic model is a modified version of the initial-boundary-value problem for age-dependent population growth described in more details in [36]. The population is divided into $n$ age groups defined by the age intervals $[a_{i-1}, a_i]$, where $a_1 < a_2 < \ldots < a_n = \infty$ (all the symbols used to describe variables and parameters are defined in Table1 and 2). The number of individuals $N_i(t)$ at time $t$ in the age interval $[a_{i-1}, a_i]$ is the integral of the age distribution function from $a_{i-1}$ to $a_i$. Assuming that the population distribution has reached a steady state with exponential growth or decay of the form $e^{qt}$, Hethcote [36] derived a system of $n$ ordinary differential equations (ODEs) for the sizes of the $n$ age groups. The simple demographic model used here divides the population into 2 gender ($k = f, m$) groups, and 17 age ($i = 1, 2, \ldots 17$) groups (12–14, 15–17, 18–19, 20–24, 25–29, 30–34, 35–39,40–44, 45–49, 50–54, 55–59, 60–64, 65–69, 70–74, 75–79, 80–84, and over 85). This age grouping is chosen to accurately account for patterns of HPV transmission among sexually active groups, cervical cancer screening patterns, and risk of cervical cancer development among females, and genital wart occurrence among both males and females. Similar age groupings have been used by other sexually transmitted diseases models [24, 25]. However, these models assumed an age of sexual debut of 15 years. By setting the age of sexual debut to 12 years, our model captures HPV transmission and disease that occurs before age 15. Recent data suggest age of first sexual intercourse is younger than 15 for some teenagers and adolescents. For example, according to data from the National Survey of Family Growth, 19% of female teenagers had had sex before age 15 in 1995, compared with 21% of male teenagers [1].

The sexually active population is further stratified into $L$ sexual activity groups ($l = 1, \ldots, L$), defined according to the gender-, sexual activity-, and age-specific rate of sex partner change per unit time $c_{kli}$. The number of sexual activity groups considered here is 3 ($L = 3$). New additions to the sexually active population enter gender $k$, sexual activity $l$, and cervical screening category $b$ ($b = 1, 2$) at rate of $B_{klb}$. Because males do not participate in cervical screening, throughout the model the subscript $b$ does not apply to them. For example, $B_{mlb} = B_{ml}$. Individuals die of non-cervical cancer related causes at an age- and gender-specific per capita death rate $\mu_{ki}$ per year and females with cervical cancer (categories $CC_s$ and $DCC_s$) also have an additional age- and stage-dependent mortality rate $\chi_{si}$ ($s = L, R, D$). It is assumed that being in any CIN or genital warts state does not pose an additional risk of death. Individuals are transferred between successive age groups at an age- and gender-specific per capita rate $d_{ki}$ per year given by [36]

$$d_{ki} = \frac{\mu_{ki} + q}{\exp[band_i \times (\mu_{ki} + q)] - 1},$$

where $band_i$ is the number of years within age group $i$. The annual growth rate $q$ of this demographic model should also satisfy a modified age-group form of

This material, provided by the authors as a supplement to Model for Assessing Human Papillomavirus Vaccination Strategies, is not part of Emerging Infectious Diseases contents.

the Lotka characteristic equations [36]

$$\begin{aligned} B_{ml} &= (d_{m1} + \mu_{m1} + q)N_{ml1}(0), \\ B_{flb} &= \varrho_b(d_{f1} + \mu_{f1} + q)N_{fl1}(0), \end{aligned}$$

where $\varrho_b$ denotes the fraction of females entering cervical screening category $b$, with $\varrho_1 + \varrho_2 = 1$.

After taking into account cervical cancer-induced mortality and replacing fertility rates in Hethcote's model [36] by recruitment rates into the sexually active population $B_{klb}$, the demographic model is given by the following system of 102 (= 17 × 2 × 3) ODEs:

$$\begin{aligned} dN_{ml1}/dt &= B_{ml} - (\mu_{m1} + d_{m1})N_{ml1} \\ dN_{mli}/dt &= d_{mi-1}N_{mli-1} - (\mu_{mi} + d_{mi})N_{mli} \\ dN_{fl1}/dt &= \sum_{b=1}^{2} B_{flb} - \sum_{s} \chi_{s1}(DCC_{sl1} + \sum_{h=1}^{2}\sum_{b=1}^{2} CC^{h}_{sl1b}) - (\mu_{f1} + d_{f1})N_{fl1} \\ dN_{fli}/dt &= d_{fi-1}N_{fli-1} - \sum_{s} \chi_{si}(DCC_{sli} + \sum_{h=1}^{2}\sum_{b=1}^{2} CC^{h}_{slib}) - (\mu_{fi} + d_{fi})N_{fli}, \end{aligned}$$

$i \geq 2$, $s = L, R, D$, where $d_{k17} = 0$. All variables, parameters, and subscripts are defined in Tables 1 and 2 and the text.

## 2.2 Estimates of the demographic model parameters

Death rates for males and females without cervical cancer are obtained from Vital Statistics data on gender- and age-specific mortality rates, all races, 2002 [51]. Cancer mortality data are obtained from Surveillance, Epidemiology, and End Results (SEER) Cancer Statistics age-specific mortality rates, 1997–2002 [75]. Because the U.S. population grew at a decennial rate of 13.2% between 1990 and 2000, the annual population growth rate was 1.23%. With recruitment rates into the sexually active population of 1.9% of the male active population and 1.7% of the female population, the largest annual growth rate $q$ that satisfies the solution of the Lotka characteristic equation was 0.5%. Therefore, the annual growth rate $q$ of this demographic model was set to zero, and $B_{klb}$ was chosen to satisfy the Lotka characteristic equation. This will also ensure that variation in the results across strategies is mainly due to epidemiologic and program features rather than peculiar characteristics of the demographic model [36]. The sensitivity of the results to this assumption will be tested using an annual population growth rate of 1.23%.

The initial population size $\eta$ is set to 100,000, divided equally between males and females. With the proportion of adults in sexually activity class $l$ given by $\omega_l$, the total number of individuals in sexual activity group $l$ is given by

$$\sum_{i} N_{kli} = \frac{1}{2}\omega_l \eta$$

This material, provided by the authors as a supplement to Model for Assessing Human Papillomavirus Vaccination Strategies, is not part of Emerging Infectious Diseases contents.

| Symbol | Description |
|---|---|
| subscripts | |
| $k$ | gender ($f$ = females, $m$ = males) |
| $i, j$ | age groups |
| $l, m$ | sexual activity groups |
| $h$ | group of HPV types (16/18 = 1, 6/11 = 2, joint = 12) |
| $s$ | stage of cervical intraepithelial neoplasia (CIN) or cancer |
| $b$ | cervical screening category (never =1, routine = 2) |
| variables | |
| $\lambda^h_{kli}$ | force of infection with group type $h$ |
| $X_{klib}$ | susceptible to all types |
| $Y^h_{klib}$ | infected with type $h$, susceptible to the other type |
| $Z^h_{klib}$ | immune against type $h$, susceptible to the other type |
| $U^h_{klib}$ | infected with type $h$, immune to the other type |
| $V_{klib}$ | vaccinated against all types |
| $S_{klib}$ | vaccinated with immunity waned |
| $W^h_{klib}$ | vaccinated and infected with type $h$ |
| $Q^h_{klib}$ | vaccinated and immune to type $h$ |
| $P^h_{klib}$ | vaccinated infected with type $h$, immune to the other type |
| $Ho^h_{li}$ | hysterectomy, vaccine, infection status $o$ (e.g., $o = X$) |
| $CIN^h_{slib}$ | undetected CIN, grade $s$, type $h$ |
| $CIS^h_{slib}$ | undetected carcinoma in situ (CIS), stage $s$, type $h$ |
| $DCIN^h_{slib}$ | detected CIN, grade $s$ |
| $DCIS^h_{slib}$ | detected CIS, stage $s$ |
| $ICIN^h_{slib}$ | treated CIN, grade $s$, infected type $h$ |
| $ICIS^h_{slib}$ | treated CIS, stage $s$, infected type $h$ |
| $TCIN_{sli}$ | treated CIN, grade $s$, immune |
| $TCIS_{sli}$ | treated CIS, stage $s$, immune |
| $CC^h_{slib}$ | undetected cervical cancer, stage $s$ |
| $DCC_{sli}$ | detected cervical cancer, stage $s$ |
| $SCC_{li}$ | cervical cancer survivor |
| $GW^h_{slib}$ | undetected genital warts |
| $DGW^h_{slib}$ | detected genital warts |
| $N_{kli}$ | number of individuals |

Table 1: Description of variables and subscripts

This material, provided by the authors as a supplement to Model for Assessing Human Papillomavirus Vaccination Strategies, is not part of Emerging Infectious Diseases contents.

| Symbol | Description |
|---|---|
| demographic parameters | |
| $B_{klb}$ | new entrants into the sexually active population |
| $\mu_{ki}$ | death rate |
| $q$ | rate of population growth |
| $d_{ki}$ | transfer rate between age groups |
| $band_i$ | number of years within age group $i$ |
| behavioral parameters | |
| $c_{kli}$ | average rate of sexual partner change |
| $\rho_{klmij}$ | probability of sexual mixing |
| $\varepsilon_1, \varepsilon_2$ | mixing parameters between age and activity groups |
| $\omega_l$ | proportion of adults in sexual activity class $l$ |
| $\varrho_b$ | fraction of females recruits entering cervical screening category $b$ |
| biological parameters | |
| $1/\sigma_{zki}$ | average duration of immunity following natural infection |
| $\gamma_{ki}^h$ | recovery from infection with HPV type $h$ |
| $\tilde{\gamma}_{ki}^h$ | probability of recovering from type $h$ only, given coinfection |
| $\bar{\gamma}_{ki}^h$ | probability of recovering from type $h$, given CIN regression |
| $\bar{\gamma}_{gki}^h$ | probability of recovering from type $h$, given genital warts regression |
| $\theta_{ks}^h$ | progression from HPV infection to CIN states |
| $\theta_{cks}^h$ | progression from coinfection to CIN states |
| $\theta_{wks}^h$ | progression from breakthrough HPV infection to CIN states |
| $\theta_{wcks}^h$ | progression from breakthrough coinfection to CIN states |
| $\theta_{gk}^h$ | progression from HPV infection to genital warts |
| $\theta_{gwk}^h$ | progression from breakthrough HPV infection to genital warts |
| $\theta_{gs}^h$ | probability genital warts are asymptomatic and not treated |
| $\pi_{is}^h$ | progression between CIN states or cancer |
| $\tau_{ks}^h$ | regression from CIN states to normal or HPV |
| $\tau_{ksg}^h$ | regression from CIN state $s$ to CIN state $g$ |
| $\tau_{gk}^h$ | regression from genital warts state to normal |
| $\beta_k^h$ | transmission probability (from sex $k'$ to sex $k$) |
| $r_k^h$ | relative risk of transmission from vaccinated people |
| $\varphi_k^h$ | relative risk of infection of a vaccinated person |
| $\alpha_k^h$ | vaccinated person relative rate of infection clearance |
| $1/\sigma_{ki}$ | average duration of vaccine protection |
| $\chi_{si}$ | cervical cancer associated death |
| $\phi_{k0b}$ | percentage of 12-year olds vaccinated |
| $\phi_{kib}$ | percentage vaccinated in age group $i$ |
| $\Delta_{ki}$ | rate of hysterectomy at age $i$ |
| $\kappa_{sib}$ | detection rate of CIN, stage $s$ |
| $\theta_{rs}^h$ | recurrence of CIN stage $s$ |
| $\Gamma_s$ | cure rate of CIN |
| $\psi_s$ | percentage of CIN stage $s$ infected after treatment |
| $\upsilon_s$ | detection of cervical cancer, stage $s$ |
| $\Omega_s$ | cure rate of cervical cancer, stage $s$ |

Table 2: Description of parameters

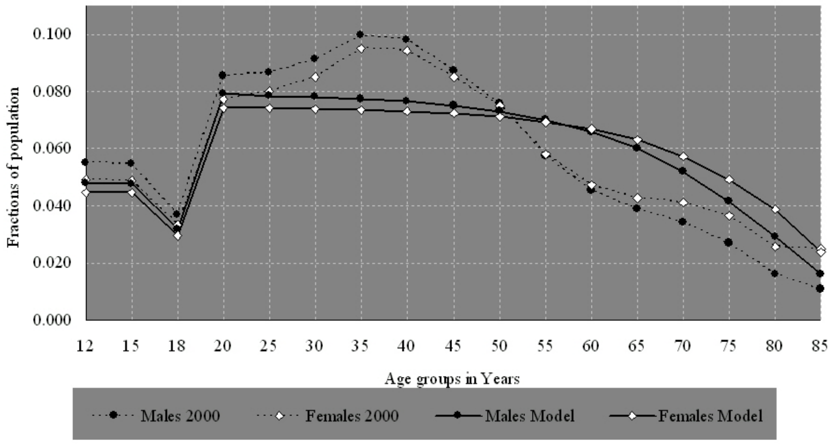


Figure 1: Age distribution for 2000 US population 12 years & above and model (0% annual growth).

By using $d_{ki-1}N_{kli-1} - (\mu_{ki} + d_{ki})N_{kli}$ with the above equation, we obtain the initial number of individuals in the youngest age group (12–14 years) of each gender and sexual activity category as

$$N_{kl1}(0) = \frac{1}{2}\omega_l \eta \left(1 + \sum_{i=2}^{17} \prod_{j=2}^{i} \frac{d_{kj-1}}{(d_{kj} + \mu_{kj})}\right)^{-1}.$$

The initial numbers of other age groups are given by

$$N_{kli}(0) = \frac{d_{ki-1}N_{kli-1}(0)}{d_{ki} + \mu_{ki}},$$

$l = 1, 2, 3$; $i = 2, 3, \ldots, 17$.

Note that the size of the male population in the model is always at a steady-state given by $\eta/2 = 50,000$. However, the size of the female population is not constant during the transient dynamics following vaccination because females are subject to additional cervical cancer-induced mortality.

The structure of the over 12-year old US population with 0% and 1.23% annual growth rates, together with data from the 2000 population census are plotted in Figures 1 and 2, respectively. The model fits well for early age groups, underestimates around age 40, and overestimates the number of people over age 40 years. It should be noted that the current model does not capture special characteristics of the US population such as the “Baby Boom” and migration.

This material, provided by the authors as a supplement to Model for Assessing Human Papillomavirus Vaccination Strategies, is not part of Emerging Infectious Diseases contents.

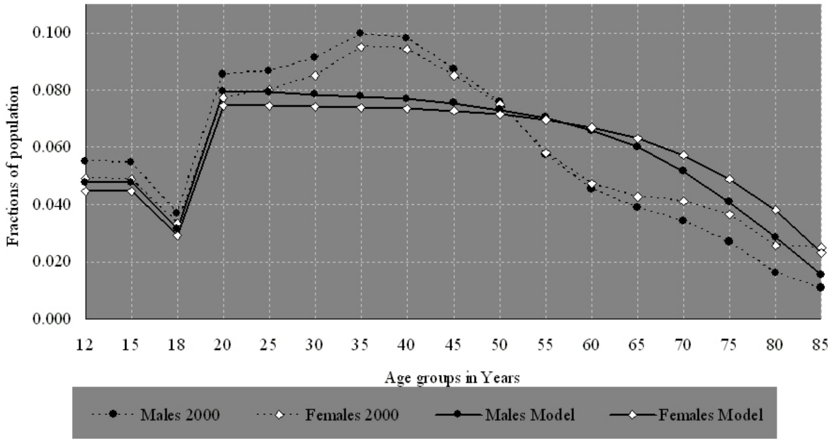


Figure 2: Age distribution for 2000 US population 12 years & above and model (1.23% annual growth).

# 3 The epidemiologic model

The epidemiologic model can be thought of as comprising three components: HPV transmission, cervical cancer development, and genital warts occurrence.

## 3.1 HPV transmission

To simplify the analysis, only three ( types 16/18 = 1, types 6/11 = 2, and coinfection =12) HPV type groupings are modeled. The sexually active host population of size $\eta$ at time $t$ is divided into distinct epidemiologic classes, depending on the host's susceptibility to infection or the host's status with respect to infection, disease, screening, and treatment. The HPV component consists of 17 epidemiologic classes $(X, V, Y, W, U, P, Z, Q)$, with each class further stratified by gender (2 groups), age (17 groups), and sexual activity (3 groups). The female population has two additional stratifications distinguishing females that are regularly screened from those who are never screened, and females who had hysterectomies from those with intact cervices. A schematical representation of the HPV transmission model is shown in Figures 3 and 4.

### 3.1.1 Susceptible individuals $X$

New additions to the sexually active population, at a rate of $B_{klb}$, enter into the uninfected (susceptible) category of gender $k$, sexual activity group $l$, and screening category $b$. A fraction of them is vaccinated at rate $\phi_{kl0b}$ and move to category $V$ and the remaining fraction enter category $X$ of susceptible individuals. The model also assumes that a proportion of individuals in other age

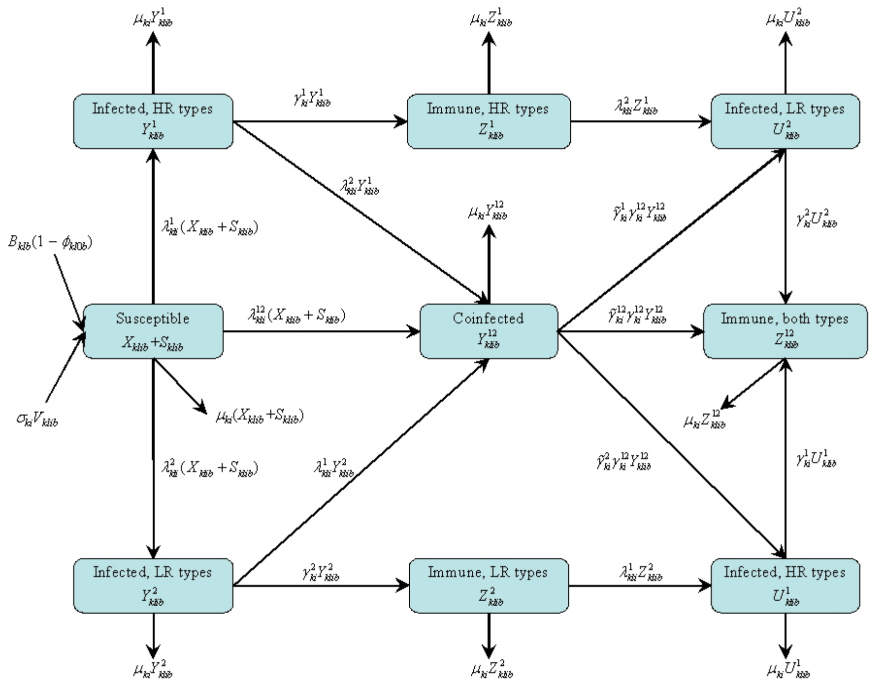


Figure 3: A simplified schematic presentation of the unvaccinated compartments of the HPV model. Individuals enter the population at rate $B_{klb}$ and a fraction $1 - \phi_{kl0b}$ of them move into the unvaccinated susceptible ($X$) compartment. Individuals leave all compartments at rate $\mu_{ki}$. A susceptible host may be infected by either or both HPV types. Susceptible individuals acquire type $h$ infection at rate $\lambda^h_{kli}$. A host infected with type $h$ can also be infected with the other type and move into compartment ($Y^{12}$). An infected individual clears infection with type $h$ at rate $\gamma^h_{ki}$. Co-infected individuals clear infection with type $h$ at rate $\tilde{\gamma}^h_{ki}\gamma^{12}_{ki}$.

This material, provided by the authors as a supplement to Model for Assessing Human Papillomavirus Vaccination Strategies, is not part of Emerging Infectious Diseases contents.

groups and epidemiologic classes is vaccinated at rate $\phi_{klib}$ and move into the vaccination classes $V$, $W$, $P$, or $Q$. It is assumed that the vaccine does not confer any therapeutic benefits to individuals already infected. Individuals in class $X$ acquire HPV infection with type $h$ at a gender, sexual activity group, age, and time dependent rate $\lambda^h_{kli}$, where $h = 1, 2, 12$. In this notation, $\lambda^1_{kli}$ denotes infection with types in group 1 (HPV 16/18) and $\lambda^{12}_{kli}$ infection with types in both groups (HPV 16/18 and HPV 6/11). The number of people in category $X_{klib}$ is reduced by infection $\lambda^h_{kli}$, vaccination $\phi_{klib}$, benign hysterectomy $\Delta_{ki}$, death from other causes $\mu_{ki}$, and aging $d_{ki}$. The ODEs for category $X$ are

$$\begin{aligned}
dX_{kl1b}/dt &= B_{klb}(1-\phi_{kl0b}) + \sum_{h\in\{1,2,12\}} \sigma^h_{zk1} Z^h_{kl1b} \\
&\quad - \sum_{h\in\{1,2,12\}} (\lambda^h_{kl1} + \phi_{kl1b} + \Delta_{k1} + \mu_{k1} + d_{k1}) X_{kl1b}, \\
dX_{klib}/dt &= d_{ki-1} X_{kli-1b} + \sum_h \sigma^h_{zki} Z^h_{klib} \\
&\quad - \sum_{h\in\{1,2,12\}} (\lambda^h_{kli} + \phi_{klib} + \Delta_{ki} + \mu_{ki} + d_{ki}) X_{klib},
\end{aligned}$$

$i = 2, \ldots, 17$; $l = 1, 2, 3$; $k = f, m$; $b = 1, 2$.

### 3.1.2 Infected individuals $Y$

When transmission occurs, the unvaccinated $X$ and vaccinated $S$ susceptible individuals enter the $Y$ class of infected individuals. Individuals enter class $Y$ after they recover from genital warts at rate $\tau_{gk}$ but are still infected with probability $1 - \bar{\gamma}^h_{gki}$ Females enter class $Y$ if their CIN spontaneously regress at rate $\tau_{fs}$ but are still infected with probability $1 - \bar{\gamma}^h_{ki}$. Individuals leave this class and enter the $Z$ class of recovered people with immunity when the infectious period for HPV ends. Unvaccinated infected individuals in the $Y$ class resolve infection at an age-, gender-, and type-specific per capita rate of $\gamma^h_{ki}$. Individuals develop CIN and genital warts at rate $\theta^h_{ks}$ and $\theta^h_{gk}$, respectively. The ODEs for category $Y^h$ are

$$\begin{aligned}
dY^h_{kl1b}/dt &= \lambda^h_{kl1}(X_{kl1b} + S_{kl1b}) + (1-\bar{\gamma}^h_{gk1})\tau^h_{gk}(GW^h_{kl1b} + DGW^h_{kl1b}) \\
&\quad + (1-\bar{\gamma}^h_{k1}) \sum_s \tau^h_{ks}(CIN^h_{sl1b} + DCIN^h_{sl1b}) \\
&\quad - (\lambda^{3-h}_{kl1} + \phi_{kl1b} + \gamma^h_{k1} + \sum_s \theta^h_{ks} + \theta^h_{gk} + \Delta_{k1} + \mu_{k1} + d_{k1}) Y^h_{kl1b}, \\
dY^h_{klib}/dt &= d_{ki-1} Y^h_{kli-1b} + \lambda^h_{kli}(X_{klib} + S_{klib}) + (1-\bar{\gamma}^h_{gki})\tau^h_{gk}(GW^h_{klib} + DGW^h_{klib}) \\
&\quad + (1-\bar{\gamma}^h_{ki}) \sum_s \tau^h_{ks}(CIN^h_{slib} + DCIN^h_{slib}) \\
&\quad - (\lambda^{3-h}_{kli} + \phi_{klib} + \gamma^h_{ki} + \sum_s \theta^h_{ks} + \theta^h_{gk} + \Delta_{ki} + \mu_{ki} + d_{ki}) Y^h_{klib}.
\end{aligned}$$

The ODEs for coinfection are given by

$$\begin{aligned}
dY^{12}_{kl1b}/dt &= \lambda^{12}_{kl1}(X_{kl1b}+S_{kl1b})+\sum_h \lambda^h_{kl1}Y^{3-h}_{kl1b}-(\phi_{kl1b}+\gamma^{12}_{k1} \\
&\quad +\sum_s \theta^{12}_{cks}+\theta^{12}_{gk}+\Delta_{k1}+\mu_{k1}+d_{k1})Y^{12}_{kl1b}, \\
dY^{12}_{klib}/dt &= d_{ki-1}Y^{12}_{kli-1b}+\sum_h \lambda^h_{kli}Y^{3-h}_{klib}-(\phi_{klib}+\gamma^{12}_{ki} \\
&\quad +\sum_s \theta^{12}_{cks}+\theta^{12}_{gk}+\Delta_{ki}+\mu_{ki}+d_{ki})Y^{12}_{klib}.
\end{aligned}$$

#### 3.1.3 Partially immune individuals $Z$

Individuals enter class $Z$ when recovered from CIN or genital warts and having resolved infection. It is assumed that immunity derived from natural infection can be temporary, and individuals in the $Z$ category can eventually move to the susceptible class $X$ at rate $\sigma^h_{zki}$. Individuals in the $Z$ class who are susceptible to one type can be infected with that type and move to class $U$. The ODEs for category $Z^h$ are

$$\begin{aligned}
dZ^h_{kl1b}/dt &= \gamma^h_{k1}Y^h_{kl1b}+\sum_{s=1}^{3}\{\bar{\gamma}^h_{k1}\tau^h_{ks}(CIN^h_{sl1b}+DCIN^h_{sl1b})+\gamma^h_{k1}ICIN^h_{sl1b}\} \\
&\quad +\sum_{s=1}^{2}\gamma^h_{k1}ICIS^h_{sl1b}+\bar{\gamma}^h_{gk1}\tau^h_{gk}(GW^h_{kl1b}+DGW^h_{kl1b}) \\
&\quad -(\lambda^{3-h}_{kl1}+\phi_{kl1b}+\Delta_{k1}+\sigma^h_{zk1}+\mu_{k1}+d_{k1})Z^h_{kl1b}, \\
dZ^h_{klib}/dt &= d_{ki-1}Z^h_{kli-1b}+\gamma^h_{ki}Y^h_{klib}+\sum_{s=1}^{3}\{\bar{\gamma}^h_{ki}\tau^h_{ks}(CIN^h_{slib}+DCIN^h_{slib}) \\
&\quad +\gamma^h_{ki}ICIN^h_{slib}\}+\sum_{s=1}^{2}\gamma^h_{ki}ICIS^h_{slib}+\bar{\gamma}^h_{gki}\tau^h_{gk}(GW^h_{klib}+DGW^h_{klib}) \\
&\quad -(\lambda^{3-h}_{kli}+\phi_{klib}+\Delta_{ki}+\sigma^h_{zki}+\mu_{ki}+d_{ki})Z^h_{klib}.
\end{aligned}$$

The ODEs for the fully immune individuals $Z^{12}$ are

$$\begin{aligned}
dZ^{12}_{kl1b}/dt &= \tilde{\gamma}^{12}_{k1}\gamma^{12}_{k1}Y^{12}_{kl1b}+\sum_h \gamma^h_{k1}U^h_{kl1b} \\
&\quad -(\phi_{kl1b}+\Delta_{k1}+\sigma^{12}_{zk1}+\mu_{k1}+d_{k1})Z^{12}_{kl1b}, \\
dZ^{12}_{klib}/dt &= \tilde{\gamma}^{12}_{ki}\gamma^{12}_{ki}Y^{12}_{klib}+\sum_h \gamma^h_{ki}U^h_{klib}-(\phi_{klib}+\Delta_{ki}+\sigma^{12}_{zki}+\mu_{ki}+d_{ki})Z^{12}_{klib}.
\end{aligned}$$

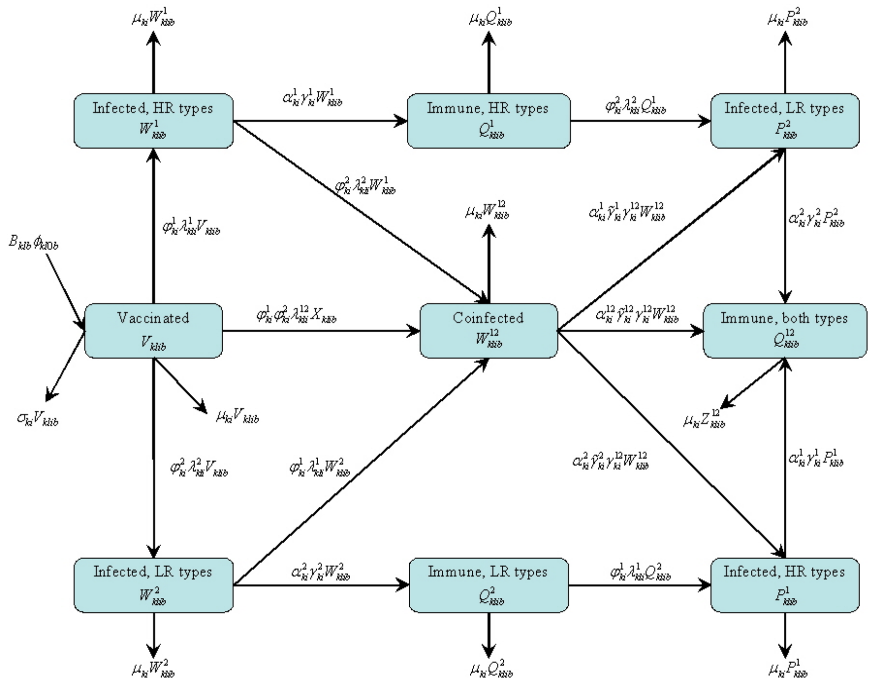


Figure 4: Schematic presentation of the vaccinated compartments of the HPV model. A fraction of the new susceptible recruits $\phi_{kl0b}$ are vaccinated and move into compartment $V$. The vaccine provides incomplete protection against the high-risk and low-risk types at rates $1-\varphi^1_{ki}$ and $1-\varphi^2_{ki}$, respectively. A vaccinated person moves into compartment $W$ upon infection with any type. Upon clearance of infection at rate $\alpha^h_{ki}$ faster than natural infection, the person moves to compartment $Q$. The vaccine-induced immunity wanes at rate $\sigma_{ki}$.

#### 3.1.4 Infected individuals with partial immunity $U$

The number of people in category $U$ is reduced by vaccination $\phi_{klib}$, resolution of infection $\gamma^h_{ki}$, and onset of disease. The ODEs for category $U$ are

$$\begin{aligned}
dU^h_{kl1b}/dt &= \lambda^h_{kl1} Z^{3-h}_{kl1b} + \tilde{\gamma}^{3-h}_{k1} \gamma^{12}_{k1} Y^{12}_{kl1b} \\
&\quad -(\phi_{kl1b} + \gamma^h_{k1} + \sum_s \theta^h_{ks} + \theta^h_{gk} + \Delta_{k1} + \mu_{k1} + d_{k1}) U^h_{kl1b}, \\
dU^h_{klib}/dt &= d_{ki-1} U^h_{kli-1b} + \lambda^h_{kli} Z^{3-h}_{klib} + \tilde{\gamma}^{3-h}_{ki} \gamma^{12}_{ki} Y^{12}_{klib} \\
&\quad -(\phi_{klib} + \gamma^h_{ki} + \sum_s \theta^h_{ks} + \theta^h_{gk} + \Delta_{ki} + \mu_{ki} + d_{ki}) U^h_{klib}.
\end{aligned}$$

### 3.1.5 Vaccinated individuals $V$

When 12-year olds are offered the vaccine, a fraction of them $\phi_{kl0}$ are vaccinated and move into the vaccination class $V$. Also, individuals in class $X$ are vaccinated at rate $\phi_{kl1b}$ and enter category $V$. The vaccine-induced immunity of those in the vaccinated class $V$ wanes, so that people eventually move to the susceptible class $S$ at an age- and gender-dependent rate $\sigma_{ki}$. It is assumed that when an individual loses vaccine-derived immunity, the individual becomes susceptible to infection with any of the types. Vaccinated individuals can also experience a break-through infection and enter the class $W$ of infective people at per capita rate $\varphi_k^h \lambda_{kli}^h$. The ODEs for category $V$ are

$$
\begin{aligned}
dV_{kl1b}/dt &= B_{klb}\phi_{kl0b} + \phi_{kl1b}X_{kl1b} - (\sum_h \varphi_k^h \lambda_{kl1}^h + \sigma_{k1} + \Delta_{k1} + \mu_{k1} + d_{k1})V_{kl1b}, \\
dV_{klib}/dt &= d_{ki-1}V_{kli-1b} + \phi_{klib}X_{klib} - (\sum_h \varphi_k^h \lambda_{kli}^h + \sigma_{ki} + \Delta_{ki} + \mu_{ki} + d_{ki})V_{klib}.
\end{aligned}
$$

### 3.1.6 Vaccinated individuals with waned immunity $S$

Individuals in this class can get infected at the same rate as those in the susceptible class $X$. The ODEs for class $S$ are

$$
\begin{aligned}
dS_{kl1b}/dt &= \sigma_{k1}V_{kl1b} - (\sum_h \lambda_{kl1}^h + \Delta_{k1} + \mu_{k1} + d_{k1})S_{kl1b}, \\
dS_{klib}/dt &= d_{ki-1}S_{kli-1b} + \sigma_{ki}V_{klib} - (\sum_h \lambda_{kli}^h + \Delta_{ki} + \mu_{ki} + d_{ki})S_{klib}.
\end{aligned}
$$

### 3.1.7 Infectious vaccinated individuals $W$

Individuals infected with one type and susceptible to the other move category $W$ when vaccinated. Vaccinated individuals are infected at an age- and gender-specific rate $\varphi_k^h$ times slower, and recover from infection at a rate $\alpha_{ki}^h$ faster than unvaccinated infected individuals and move to class $Q$. They also progress to disease at a different rate ($\theta_{wks}^h$ or $\theta_{gkw}^h$) compared with that of infected unvaccinated individuals. The ODEs for category $W$ are

$$
\begin{aligned}
dW_{kl1b}^h/dt &= \varphi_k^h \lambda_{kl1}^h V_{kl1b} + \phi_{kl1b}Y_{kl1b}^h - (\varphi_k^{3-h}\lambda_{kl1}^{3-h} + \alpha_{k1}^h \gamma_{k1}^h \\
&\quad + \sum_s \theta_{wks}^h + \theta_{gkw}^h + \Delta_{k1} + \mu_{k1} + d_{k1})W_{kl1b}^h, \\
dW_{klib}^h/dt &= d_{ki-1}W_{kli-1b}^h + \varphi_k^h \lambda_{kli}^h V_{klib} + \phi_{klib}Y_{klib}^h - (\varphi_k^{3-h}\lambda_{kli}^{3-h} \\
&\quad + \alpha_{ki}^h \gamma_{ki}^h + \sum_s \theta_{wks}^h + \theta_{gkw}^h + \Delta_{ki} + \mu_{ki} + d_{ki})W_{klib}^h.
\end{aligned}
$$

The ODEs for coinfection $W^{12}$ are

$$\begin{aligned}
dW^{12}_{kl1b}/dt &= \varphi^1_k\varphi^2_k\lambda^{12}_{kl1}V_{kl1b} + \sum_h \varphi^h_k\lambda^h_{kl1}W^{3-h}_{kl1b} + \phi_{kl1b}Y^{12}_{kl1b} - (\alpha^{12}_{k1}\gamma^{12}_{k1} \\
&\quad + \sum_s \theta^{12}_{wcks} + \theta^{12}_{kgw} + \Delta_{k1} + \mu_{k1} + d_{k1})W^{12}_{kl1b}, \\
dW^{12}_{klib}/dt &= d_{ki-1}W^{12}_{kli-1b} + \varphi^1_k\varphi^2_k\lambda^{12}_{kli}V_{klib} + \sum_h \varphi^h_k\lambda^h_{kli}W^{3-h}_{klib} + \phi_{klib}Y^{12}_{klib} \\
&\quad -(\alpha^{12}_{ki}\gamma^{12}_{ki} + \sum_s \theta^{12}_{wcks} + \theta^{12}_{gwk} + \Delta_{ki} + \mu_{ki} + d_{ki})W^{12}_{klib}.
\end{aligned}$$

#### 3.1.8 Vaccinated, partially immune individuals $Q$

Infected vaccinated individuals (category $W$) recovering from infection and individuals with natural immunity to one type (category $Z$) receiving the vaccine move to category $Q$. Individuals in this class who are susceptible to one type can be infected with that type and move to class $P$. The ODEs for category $Q$ are

$$\begin{aligned}
dQ^h_{kl1b}/dt &= \alpha^h_{k1}\gamma^h_{k1}W^h_{kl1b} + \phi_{kl1b}Z^h_{kl1b} - (\varphi^{3-h}_k\lambda^{3-h}_{kl1} + \Delta_{k1} + \mu_{k1} + d_{k1})Q^h_{kl1b}, \\
dQ^h_{klib}/dt &= d_{ki-1}Q^h_{kli-1b} + \alpha^h_{ki}\gamma^h_{ki}W^h_{klib} + \phi_{klib}Z^h_{klib} \\
&\quad -(\varphi^{3-h}_k\lambda^{3-h}_{kli} + \Delta_{ki} + \mu_{ki} + d_{ki})Q^h_{klib}.
\end{aligned}$$

The ODEs for $Q^{12}$ are

$$\begin{aligned}
dQ^{12}_{kl1b}/dt &= \alpha^{12}_{k1}\tilde{\gamma}^{12}_{k1}\gamma^{12}_{k1}W^{12}_{kl1b} + \sum_h \gamma^h_{k1}P^h_{kl1b} + \phi_{kl1b}Z^{12}_{kl1b} \\
&\quad -(\Delta_{k1} + \mu_{k1} + d_{k1})Q^{12}_{klib}, \\
dQ^{12}_{klib}/dt &= d_{ki-1}Q^{12}_{kli-1b} + \alpha^{12}_{ki}\tilde{\gamma}^{12}_{ki}\gamma^{12}_{ki}W^{12}_{klib} + \sum_h \gamma^h_{ki}P^h_{klib} + \phi_{klib}Z^{12}_{klib} \\
&\quad -(\Delta_{ki} + \mu_{ki} + d_{ki})Q^{12}_{klib}.
\end{aligned}$$

#### 3.1.9 Vaccinated, infected individuals with partial immunity $P$

Coinfected vaccinated individuals recovering from one infection (category $W^{12}$), vaccinated individuals (category $Q$) getiing infected, and individuals infected with one type (category $Z$) receiving the vaccine move to category $P$. The ODEs for category $P$ are

$$\begin{aligned}
dP^h_{kl1b}/dt &= \varphi^h_k\lambda^h_{kl1}Q^{3-h}_{kl1b} + \alpha^{3-h}_{k1}\tilde{\gamma}^{3-h}_{k1}\gamma^{12}_{k1}W^{12}_{kl1b} + \phi_{kl1b}U^h_{kl1b} \\
&\quad -(\alpha^h_{k1}\gamma^h_{k1} + \sum_s \theta^h_{wks} + \theta^h_{gwk} + \Delta_{k1} + \mu_{k1} + d_{k1})P^h_{kl1b}, \\
dP^h_{klib}/dt &= d_{ki-1}P^h_{kli-1b} + \varphi^h_k\lambda^h_{kli}Q^{3-h}_{klib} + \alpha^{3-h}_{ki}\tilde{\gamma}^{3-h}_{ki}\gamma^{12}_{ki}W^{12}_{klib} + \phi_{klib}U^h_{klib} \\
&\quad -(\alpha^h_{ki}\gamma^h_{ki} + \sum_s \theta^h_{wks} + \theta^h_{gwk} + \Delta_{ki} + \mu_{ki} + d_{ki})P^h_{klib}.
\end{aligned}$$

Note that for males, $\Delta_{mi} = \theta^h_{wms} = \tau^h_{ms} = \theta^h_{ms} = \theta^h_{cms} = 0$.

## 3.2 Cervical intraepithelial neoplasia

Infected females (whether vaccinated or not) can develop CIN and move to the CIN segment of the model. There are several states that represent the true histological health status of a female: infected with a normal cervix, CIN grade 1 (CIN 1), CIN grade 2 (CIN 2), and CIN grade 3 (CIN 3). Females in the CIN and cancer stages are further classified into unknown, detected, or treated classes. There are also two additional absorbing states where only females who are no longer at risk of developing cervical cancer enter. These are benign hysterectomy for reasons other than cervical cancer (at an age-specific rate $\Delta_{fi}$) and treated and cured CIN at stage-specific rate $(1-\psi_s)\Gamma_s$. Females in these two states are considered to be at no risk of developing cervical cancer [61]. However, females with hysterectomies for benign conditions can be infected and are at risk of developing genital warts [9]. Further, to take into account the fact that treatment of CIN does not completely eliminate the virus, another category of women with treated CIN who remain infected after treatment ($ICIN$) was created. Females enter this category from the detected state at rate $\psi_s\Gamma_s$ and stay there until their CIN recurs at rate $\theta^h_{rs}$ or they clear infection.

An infected female with a normal cervix can only directly progress to $CIN^h_s$ (at rate $\theta^h_{fs}$ if unvaccinated or $\theta^h_{wfs}$ if vaccinated), die due to causes other than cervical cancer, or remain infected without progressing to CIN (Figure 5). The respective progression rates given coinfection are $\theta^h_{cfs}$ and $\theta^h_{cwfs}$. For the base case, it is assumed that cases with coinfection progress to CIN according to the rate of high-risk HPV types. That is, $\theta^1_{cfs}=\theta^1_{fs}$, $\theta^2_{cfs}=0$, $\theta^1_{cwfs}=\theta^1_{wfs}$, and $\theta^2_{cwfs}=0$. It is assumed that infected females classified as CIN can progress only to higher CIN states (CIN1 to CIN2, CIN2 to CIN3), or cancer (CIN3 to cervical carcinoma in situ, CIS) at rate $\pi^h_{si}$, regress to normal at rate $\tau^h_s$ or CIN state $g$ at rate $\tau^h_{sg}$, die from other causes, be detected at rate $\kappa_{sib}$ and be treated and cured at rate $\Gamma_s$, or remain in that CIN state. Coinfection of females in CIN and cervical cancer states is not modeled. It is assumed that regression from CIN states does not necessarily imply recovery from HPV infection. A female whose CIN regresses to normal but is still infected moves to the infected category $Y^h_{fi}$ at an age- and stage-specific rate $\tau^h_s(1-\bar{\gamma}^h_{fi})$ regardless of her vaccination status. Only mutual regression from both HPV and CIN confers immunity against that type. Females regressing from CIN, whose HPV infection clears, move into class $Z$ at an age- and state-specific rate $\bar{\gamma}^h_{fi}\tau^h_s$ $(s=1,2,3)$.

The cervical neoplasia segment includes several epidemiologic classes ($CIN_s$, $DCIN_s$, $TCIN_s$, $ICIN_s$; $s=1,2,3$), with each class further subdivided into age (= 17), sexual activity (= 3), and screening (= 2) groups.

### 3.2.1 Undetected CIN $CIN_s$

The number of females with undetected CIN increases as infected females develop disease or fail treatment. Screening $\kappa_{sib}$, spontaneous regression $\tau^h_{fs}$, and progression to higher disease grades $\pi^h_{si}$ reduce the number of females in this

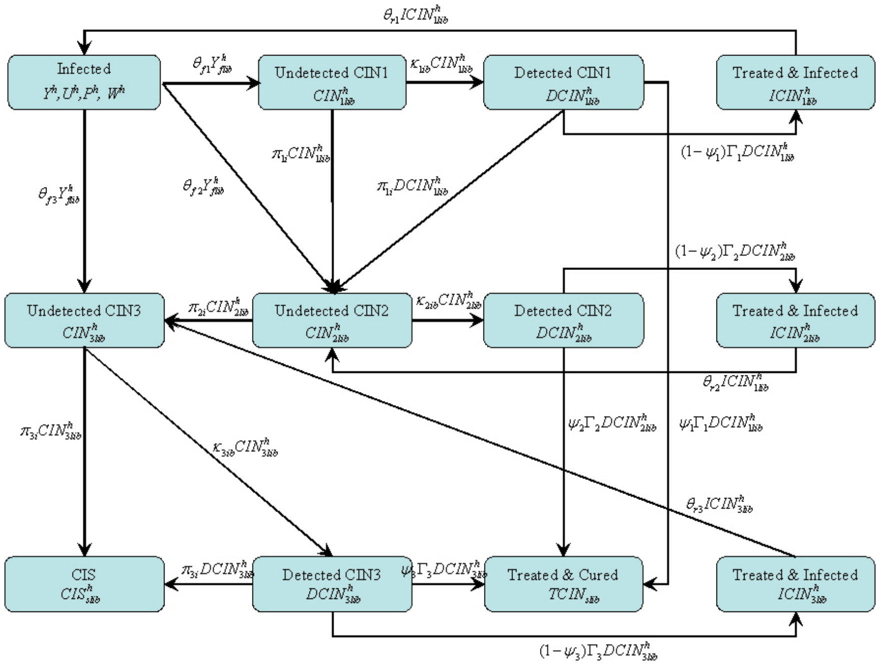


Figure 5: A simplified schematic presentation of the cervical intraepithelial neoplasia (CIN) model. Females can develop cervical intraepithelial neoplasia (CIN) and progress though several histological states: infected with a normal cervix, CIN 1, CIN 2, CIN 3, and cervical carcinoma in situ (CIS). Females with CIN can regress to normal with or without infection.

This material, provided by the authors as a supplement to Model for Assessing Human Papillomavirus Vaccination Strategies, is not part of Emerging Infectious Diseases contents.

category. Equations for undetected CIN are

$$
\begin{aligned}
dCIN^h_{1l1b}/dt &= \theta^h_{f1}(Y^h_{fl1b}+U^h_{fl1b})+\theta^h_{cf1}Y^{12}_{fl1b}+\theta^h_{wf1}(W^h_{fl1b}+P^h_{fl1b}) \\
&\quad +\theta^h_{wcf1}W^{12}_{fl1b}+\theta^h_{r1}ICIN^h_{1l1b}+\tau^h_{f21}CIN^h_{2l1b}+\tau^h_{f31}CIN^h_{3l1b} \\
&\quad -(\tau^h_{f1}+\pi^h_{11}+\Delta_{f1}+\kappa_{11b}+\mu_{f1}+d_{f1})CIN^h_{1l1b}, \\
dCIN^h_{1lib}/dt &= d_{fi-1}CIN^h_{1li-1b}+\theta^h_{f1}(Y^h_{flib}+U^h_{flib})+\theta^h_{cf1}Y^{12}_{flib} \\
&\quad +\theta^h_{wfi}(W^h_{flib}+P^h_{flib})+\theta^h_{wcfi}W^{12}_{flib}+\theta_{rs}ICIN^h_{1lib} \\
&\quad +\tau^h_{f21}CIN^h_{2lib}+\tau^h_{f31}CIN^h_{3lib} \\
&\quad -(\tau^h_{f1}+\pi^h_{1i}+\Delta_{fi}+\kappa_{1ib}+\mu_{fi}+d_{fi})CIN^h_{1lib},
\end{aligned}
$$

$$
\begin{aligned}
dCIN^h_{sl1b}/dt &= \theta^h_{fs}(Y^h_{fl1b}+U^h_{fl1b})+\theta^h_{cfs}Y^{12}_{fl1}+\theta^h_{wfs}(W^h_{fl1b}+P^h_{fl1b}) \\
&\quad +\theta^h_{wcfs}W^{12}_{fl1b}+\theta^h_{rs}ICIN^h_{1l1b} \\
&\quad +\pi^h_{s-11}(CIN^h_{s-1l1b}+DCIN^h_{s-1l1b}) \\
&\quad +\tau^h_{fs+1s}CIN^h_{s+1l1b}-(\tau^h_{fs}+\tau^h_{fss-1}+\tau^h_{fss-2}+\pi^h_{s1}+\Delta_{f1} \\
&\quad +\kappa_{s1b}+\mu_{f1}+d_{f1})CIN^h_{sl1b},
\end{aligned}
$$

$$
\begin{aligned}
dCIN^h_{slib}/dt &= d_{fi-1}CIN^h_{sli-1b}+\theta^h_{fs}(Y^h_{flib}+U^h_{flib})+\theta^h_{cfs}Y^{12}_{flib} \\
&\quad +\theta^h_{wfs}(W^h_{flib}+P^h_{flib})+\theta^h_{wcfi}W^{12}_{flib}+\theta^h_{rs}ICIN^h_{1lib} \\
&\quad +\pi^h_{s-1i}(CIN^h_{s-1lib}+DCIN^h_{s-1lib})+\tau^h_{fs+1s}CIN^h_{s+1lib} \\
&\quad -(\tau^h_{fs}+\tau^h_{fss-1}+\tau^h_{fss-2}+\pi^h_{si}+\Delta_{fi} \\
&\quad +\kappa_{sib}+\mu_{fi}+d_{fi})CIN^h_{slib},
\end{aligned}
$$

where $s = 2, 3$, and $\tau^h_{f43} = \tau^h_{f20} = 0$.

### 3.2.2 Detected CIN $DCIN_s$

Detection of CIN occurs only as result of screening at rate $\kappa_{sib}$. This rate depends on screening coverage and the characteristics of the screening and diagnostic tests. If it does not regress at rate $\tau^h_{fs}$ or is treated at rate $\Gamma_s$, CIN can progress to a higher grade at rate $\pi^h_{si}$. Equations for detected CIN are

$$
\begin{aligned}
dDCIN^h_{sl1b}/dt &= \kappa_{s1b}CIN^h_{sl1b}-(\tau^h_{fs}+\pi^h_{s1}+\Delta_{f1}+\Gamma_s+\mu_{f1}+d_{f1})DCIN^h_{sl1b}, \\
dDCIN^h_{slib}/dt &= d_{fi-1}DCIN^h_{sli-1b}+\kappa_{sib}CIN^h_{slib} \\
&\quad -(\tau^h_{fs}+\pi^h_{si}+\Delta_{fi}+\Gamma_s+\mu_{fi}+d_{fi})DCIN^h_{slib},
\end{aligned}
$$

where $s = 1, 2, 3$.

This material, provided by the authors as a supplement to Model for Assessing Human Papillomavirus Vaccination Strategies, is not part of Emerging Infectious Diseases contents.

#### 3.2.3 Treated CIN $TCIN_s$

It is assumed that treatment does not completely eliminate infection. A fraction of treated females $\psi_s$ will remain infectious after treatment and move to the category treated but infectious $ICIN_s$. Equations for treated CIN are

$$\begin{aligned}
dTCIN_{sl1}/dt &= (1-\psi_s)\Gamma_s \sum_h \sum_b DCIN^h_{sl1b} - (\Delta_{f1} + \mu_{f1} + d_{f1})TCIN_{sl1}, \\
dTCIN_{sli}/dt &= d_{fi-1}TCIN_{li-1} + (1-\psi_s)\Gamma_s \sum_h \sum_b DCIN^h_{slib} \\
&\quad -(\Delta_{fi} + \mu_{fi} + d_{fi})TCIN_{sli},
\end{aligned}$$

where $s = 1, 2, 3$.

#### 3.2.4 Treated CIN but infectious $ICIN_s$

CIN for females in this category can recur at rate $\theta_{rs}$ and move to category $CIN_s$. Infection can also resolve and individuals enter category $Z^h$. Equations for treated but infectious CIN are

$$\begin{aligned}
dICIN^h_{sl1b}/dt &= \psi_s\Gamma_s DCIN^h_{sl1b} - (\gamma^h_{f1} + \theta^h_{rs} + \Delta_1 + \mu_{f1} + d_{f1})ICIN^h_{sl1b}, \\
dICIN^h_{slib}/dt &= d_{fi-1}ICIN^h_{sli-1b} + \psi_s\Gamma_s DCIN^h_{slib} \\
&\quad -(\gamma^h_{fi} + \theta^h_{rs} + \Delta_i + \mu_{fi} + d_{fi})ICIN^h_{slib},
\end{aligned}$$

where $s = 1, 2, 3$.

### 3.3 Cervical carcinoma in situ

It is assumed that females classified as CIN can progress to carcinoma in situ (CIS). Because females spend, on average, a long time in CIS, two CIS states are modeled (CIS 1 and CIS 2). It is assumed that regression from CIS states is not possible. CIS is further divided into several epidemiologic classes ($CIS_s$, $DCIS_s$, $TCIS_s$, $ICIS_s$; $s = 1, 2$), with each class further subdivided into age (= 17), sexual activity (= 3), and screening (= 2) groups.

#### 3.3.1 Undetected CIS $CIS_s$

The number of females with undetected CIS increases as they progress from CIN 3 (severe dysplasia) or fail treatment. Screening $\kappa_{3+sib}$ and progression to higher disease grades $\pi^h_{3+si}$ reduce the number of females in this category, $s = 1, 2$. Equations for undetected CIS are

$$\begin{aligned}
dCIS^h_{1l1b}/dt &= \theta^h_{r4}ICIS^h_{1l1b} + \pi^h_{31}(CIN^h_{3l1b} + DCIN^h_{3l1b}) \\
&\quad -(\pi^h_{41} + \Delta_{f1} + \kappa_{41b} + \mu_{f1} + d_{f1})CIS^h_{1l1b}, \\
dCIS^h_{1lib}/dt &= d_{fi-1}CIS^h_{1li-1b} + \theta^h_{r4}ICIS^h_{1lib} + \pi^h_{3i}(CIN^h_{3lib} + DCIN^h_{3lib}) \\
&\quad -(\pi^h_{4i} + \Delta_{fi} + \kappa_{4ib} + \mu_{fi} + d_{fi})CIN^h_{1lib},
\end{aligned}$$

This material, provided by the authors as a supplement to Model for Assessing Human Papillomavirus Vaccination Strategies, is not part of Emerging Infectious Diseases contents.

$$\begin{aligned}
dCIS^h_{2l1b}/dt &= \theta^h_{r5}ICIS^h_{2l1b} + \pi^h_{41}(CIS^h_{1l1b} + DCIS^h_{1l1b}) \\
&\quad -(\pi^h_{51} + \Delta_{f1} + \kappa_{51b} + \mu_{f1} + d_{f1})CIS^h_{2l1b}, \\
dCIS^h_{2lib}/dt &= d_{fi-1}CIS^h_{2li-1b} + \theta^h_{r5}ICIS^h_{2lib} + \pi^h_{4i}(CIS^h_{2lib} + DCIS^h_{2lib}) \\
&\quad -(\pi^h_{51} + \Delta_{f1} + \kappa_{51b} + \mu_{f1} + d_{f1})CIS^h_{2l1b}.
\end{aligned}$$

#### 3.3.2 Detected CIS $DCIS_s$

Detection of CIS occurs only as result of screening at rate $\kappa_{3+sib}$. If it is not treated and cured at rate $\Gamma_{3+s}$, CIS can progress to a higher grade $\pi^h_{3+si}$ or cancer. Equations for detected CIS are

$$\begin{aligned}
dDCIS^h_{sl1b}/dt &= \kappa_{3+s1b}CIS^h_{sl1b} - (\pi^h_{3+s1} + \Delta_{f1} + \Gamma_{3+s} + \mu_{f1} + d_{f1})DCIS^h_{sl1b}, \\
dDCIS^h_{slib}/dt &= d_{fi-1}DCIS^h_{sli-1b} + \kappa_{3+sib}CIS^h_{slib} \\
&\quad -(\pi^h_{3+si} + \Delta_{fi} + \Gamma_{3+s} + \mu_{fi} + d_{fi})DCIS^h_{slib},
\end{aligned}$$

where $s = 1, 2$.

#### 3.3.3 Treated CIS $TCIS_s$

It is assumed that treatment does not completely eliminate infection. A fraction of treated females $\psi_{3+s}$ will remain infectious after treatment and move to the category treated but infectious $ICIS_s$. Equations for treated CIS are

$$\begin{aligned}
dTCIS_{sl1}/dt &= (1 - \psi_{3+s})\Gamma_{3+s}\sum_h\sum_b DCIS^h_{sl1b} - (\Delta_{f1} + \mu_{f1} + d_{f1})TCIS_{sl1}, \\
dTCIS_{sli}/dt &= d_{fi-1}TCIS_{li-1} + (1 - \psi_{3+s})\Gamma_{3+s}\sum_h\sum_b DCIS^h_{slib} \\
&\quad -(\Delta_{fi} + \mu_{fi} + d_{fi})TCIS_{sli},
\end{aligned}$$

where $s = 1, 2$.

#### 3.3.4 Treated CIS but infectious $ICIS_s$

It is assumed that CIS recurs at rate $\theta^h_{r3+s}$ and women with recuring CIS move to category $CIS_s$. Infection can also resolve and individuals enter category $Z^h$. Equations for treated but infectious CIS are

$$\begin{aligned}
dICIS^h_{sl1b}/dt &= \psi_{3+s}\Gamma_{3+s}DCIS^h_{sl1b} - (\gamma^h_{f1} + \theta^h_{r3+s} + \Delta_1 + \mu_{f1} + d_{f1})ICIS^h_{sl1b}, \\
dICIS^h_{slib}/dt &= d_{fi-1}ICIS^h_{sli-1b} + \psi_{3+s}\Gamma_{3+s}DCIN^h_{slib} \\
&\quad -(\gamma^h_{fi} + \theta^h_{r3+s} + \Delta_i + \mu_{fi} + d_{fi})ICIS^h_{slib},
\end{aligned}$$

where $s = 1, 2$.

This material, provided by the authors as a supplement to Model for Assessing Human Papillomavirus Vaccination Strategies, is not part of Emerging Infectious Diseases contents.

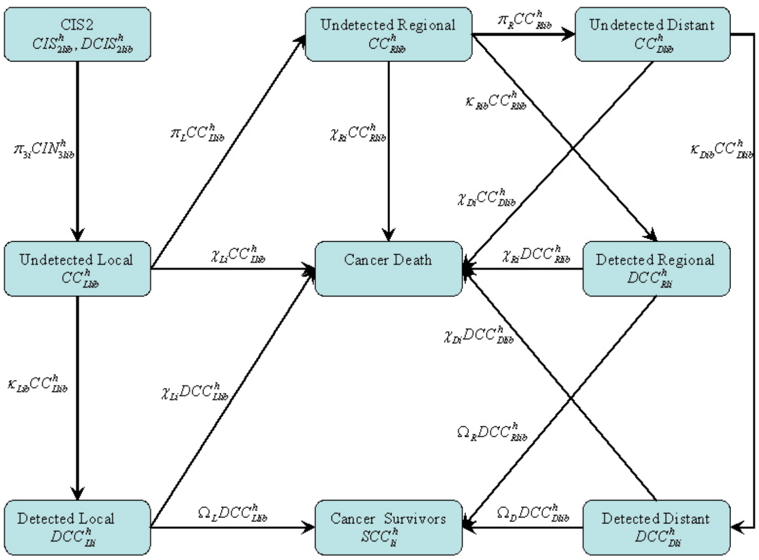


Figure 6: Schematic presentation of the cervical cancer (CC) model. Females can develop cervical cancer from carcinoma in situ stage 2 (CIS2) and progress though several cancer stages: early invasive local cervical cancer, regional late invasive cervical cancer, and distant late invasive cervical cancer. If detected, cancer cases are treated. After successful treatment, those cases move to the cancer survivors compartment. Cancer cases die at rate $\chi_{si}$.

## 3.4 Cervical cancer

There are several states that represent the health status of a female with cervical cancer: localized cervical cancer (LCC), regional cervical cancer (RCC), distant cervical cancer (DCC), and cancer survivors who are free from cancer (Figure 6). Females in cancer stages are further classified into unknown, detected, or treated classes. A female with an invasive cancer can progress only to the next higher cancer state $CC_s^h$ (LCC to RCC, RCC to DCC) at rate $\pi_{si}$ ($s = L, R$), her cervical cancer is detected at rate $\upsilon_{sib}$ and successfully treated and move to the cancer survivors state at rate $\Omega_s$, die from cancer at rate $\chi_{si}$, or stay in that undetected cancer state. Regression from invasive cancer to normal is not allowed. It is assumed that females who were successfully treated for invasive cancer are no longer infectious.

### 3.4.1 Undetected cervical cancer $CC_s$

CIS 2 cases that are not detected and treated can progress to localized cervical cancer at rate $\pi_{5i}^h$. Undetected cancer cases, if undetected at rate $\upsilon_{sib}$, can progress to more advanced stages at rate $\pi_s$, $s = L, R$. Cervical cancer has an

This material, provided by the authors as a supplement to Model for Assessing Human Papillomavirus Vaccination Strategies, is not part of Emerging Infectious Diseases contents.

additional mortality rate $\chi_{si}$. Equations for undetected $CC$ are

$$
\begin{aligned}
dCC^h_{Ll1b}/dt &= \pi^h_{51}(CIS^h_{2l1b} + DCIS^h_{2l1b}) - (\pi_L + \upsilon_{L1b} + \chi_{L1} + \mu_{f1} + d_{f1})CC^h_{Ll1b},\\
dCC^h_{Llib}/dt &= d_{fi-1}CC^h_{Lli-1b} + \pi^h_{5i}(CIS^h_{5lib} + DCIS^h_{5lib})\\
&\quad -(\pi_L + \upsilon_{Lib} + \chi_{Li} + \mu_{fi} + d_{fi})CC^h_{Llib},\\
dCC^h_{Rl1b}/dt &= \pi_L CC^h_{Ll1b} - (\pi_R + \upsilon_{R1b} + \chi_{R1} + \mu_{f1} + d_{f1})CC^h_{Rl1b},\\
dCC^h_{Rlib}/dt &= d_{fi-1}CC^h_{Rli-1b} + \pi_L CC^h_{Llib} - (\pi_D + \upsilon_{Rib} + \chi_{Ri} + \mu_{fi} + d_{fi})CC^h_{Rlib},\\
dCC^h_{Dl1b}/dt &= \pi_R CC^h_{Rl1b} - (\upsilon_{D1b} + \chi_{D1} + \mu_{f1} + d_{f1})CC^h_{Dl1b},\\
dCC^h_{Dlib}/dt &= \pi_R CC^h_{Rlib} + d_{fi-1}CC^h_{Dli-1b} - (\upsilon_{Dib} + \chi_{Di} + \mu_{fi} + d_{fi})CC^h_{Dlib},
\end{aligned}
$$

where $i \geq 2$.

#### 3.4.2 Detected cervical cancer $DCC_s$

Detected cancer cases are treated and cured at rate $\Omega_s$ and move to the cancer survivors category $SCC$. Equations for detected $CC$ are

$$
\begin{aligned}
dDCC_{sl1}/dt &= \sum_h \sum_b \upsilon_{s1b} CC^h_{sl1b} - (\Omega_s + \chi_{s1} + \mu_{f1} + d_{f1})DCC_{sl1},\\
dDCC_{sli}/dt &= d_{fi-1}DCC_{sli-1} + \sum_h \sum_b \upsilon_{sib} CC^h_{slib} - (\Omega_s + \chi_{si} + \mu_{fi} + d_{fi})DCC_{sli},
\end{aligned}
$$

where $s = L, R, D$.

#### 3.4.3 Cervical cancer survivors $SCC$

Equations for cancer survivors are

$$
\begin{aligned}
dSCC_{l1}/dt &= \sum_s \Omega_s DCC_{sl1} - (\mu_{f1} + d_{f1})SCC_{l1},\\
dSCC_{li}/dt &= d_{fi-1}SCC_{li-1} + \sum_s \Omega_s DCC_{sli} - (\mu_{fi} + d_{fi})SCC_{li}.
\end{aligned}
$$

### 3.5 Genital warts $GW$

Individuals (whether vaccinated or not) infected with HPV 6/11 can develop genital warts at rate $\theta^2_{gwk}$ and move to the genital warts class $GW$. Of those, a proportion $\theta_{gs}$ will remain asymptomatic and will not be treated whereas the rest will be recognized and treated. Individuals recovering from genital warts at rate $\tau^2_{kg}$ move to class $Z$. It is assumed that only infection with HPV 6/11 can cause genital warts whereas infection with HPV 16/18 does not lead to genital

This material, provided by the authors as a supplement to Model for Assessing Human Papillomavirus Vaccination Strategies, is not part of Emerging Infectious Diseases contents.

warts [84]. The asymptomatic genital warts class consists of the following ODEs

$$\begin{aligned}
dGW^2_{kl1b}/dt &= \theta_{gs}(\theta^2_{gk}(Y^2_{kl1b} + U^2_{kl1b}) + \theta^{12}_{gk}Y^{12}_{kl1b} + \theta^2_{gwk}(W^2_{kl1b} + P^2_{kl1b}) \\
&\quad + \theta^{12}_{gwk}W^{12}_{kl1b}) - (\tau^2_{gk} + \Delta_{k1} + \mu_{k1} + d_{k1})GW^2_{kl1b}, \\
dGW^2_{klib}/dt &= d_{ki-1}GW^2_{kli-1b} + \theta_{gs}(\theta^2_{gk}(Y^2_{klib} + U^2_{klib}) + \theta^{12}_{gk}Y^{12}_{klib} \\
&\quad + \theta^2_{gwk}(W^2_{klib} + P^2_{klib}) + \theta^{12}_{gwk}W^{12}_{klib}) \\
&\quad - (\tau^2_{gk} + \Delta_{ki} + \mu_{ki} + d_{ki})GW^2_{klib}.
\end{aligned}$$

The symptomatic genital warts class consists of the following ODEs

$$\begin{aligned}
dDGW^2_{kl1b}/dt &= (1 - \theta_{gs})(\theta^2_{gk}(Y^2_{kl1b} + U^2_{kl1b}) + \theta^{12}_{gk}Y^{12}_{kl1b} \\
&\quad + \theta^2_{gwk}(W^2_{kl1b} + P^2_{kl1b}) + \theta^{12}_{gwk}W^{12}_{kl1b}) \\
&\quad - (\tau^2_{gk} + \Delta_{k1} + \mu_{k1} + d_{k1})DGW^2_{kl1b}, \\
dDGW^2_{klib}/dt &= d_{ki-1}GW^2_{kli-1b} + (1 - \theta_{gs})(\theta^2_{gk}(Y^2_{klib} + U^2_{klib}) \\
&\quad + \theta^{12}_{gk}Y^{12}_{klib} + \theta^2_{gwk}(W^2_{klib} + P^2_{klib}) + \theta^{12}_{gwk}W^{12}_{klib}) \\
&\quad - (\tau^2_{gk} + \Delta_{ki} + \mu_{ki} + d_{ki})DGW^2_{klib}.
\end{aligned}$$

## 3.6 Hysterectomies for benign conditions

Females who undergo hysterectomies for benign conditions move to the $H$ compartment and stay there at no risk of developing CIN or cervical cancer. However, females in this compartment can be infected, can transmit infection, and can develop genital warts. There are several epidemiologic classes within the $H$ compartment $(HX, HV, HS, HY, HW, HU, HP, HZ, HQ, HGW)$, with each class further stratified by age (=17) and sexual activity (=3) groups.

### 3.6.1 Susceptible individuals $HX$

The ODEs for category $HX$ are

$$\begin{aligned}
dHX_{fl1}/dt &= \Delta_{f1}\sum_b X_{fl1b} + \sum_h \sigma^h_{zf1}HZ^h_{fl1b} - (\sum_h \lambda^h_{fl1} + \mu_{f1} + d_{f1})HX_{fl1}, \\
dHX_{fli}/dt &= d_{fi-1}HX_{fli-1} + \Delta_{fi}\sum_b X_{flib} + \sum_h \sigma^h_{zfi}HZ^h_{flib} \\
&\quad - (\sum_h \lambda^h_{fli} + \mu_{fi} + d_{fi})HX_{fli},
\end{aligned}$$

This material, provided by the authors as a supplement to Model for Assessing Human Papillomavirus Vaccination Strategies, is not part of Emerging Infectious Diseases contents.

### 3.6.2 Infected individuals $HY$

The ODEs for category $HY$ are

$$\begin{aligned} dHY^h_{fl1}/dt &= \lambda^h_{fl1}(HX_{fl1}+HS_{fl1}) - (\lambda^{3-h}_{fl1}+\gamma^h_{f1}+\theta^h_{gf}+\mu_{f1}+d_{f1})HY^h_{fl1} \\ &\quad +\Delta_{f1}(\sum_b Y^h_{fl1b}+\sum_s(CIN^h_{sl1b}+DCIN^h_{sl1b}+ICIN^h_{sl1b})), \\ dHY^h_{fli}/dt &= d_{fi-1}HY^h_{fli-1}+\lambda^h_{fli}(HX_{fli}+HS_{fli}) \\ &\quad -(\lambda^{3-h}_{fli}+\gamma^h_{fi}+\theta^h_{gf}+\mu_{fi}+d_{fi})HY^h_{fli} \\ &\quad +\Delta_{fi}(\sum_b Y^h_{flib}+\sum_s(CIN^h_{slib}+DCIN^h_{slib}+ICIN^h_{slib})). \end{aligned}$$

The ODEs for $HY^{12}$ are

$$\begin{aligned} dHY^{12}_{fl1}/dt &= \lambda^{12}_{fl1}(HX_{fl1}+HS_{fl1})+\sum_h \lambda^h_{fl1}HY^{3-h}_{fl1}+\Delta_{f1}\sum_b Y^{12}_{fl1b} \\ &\quad -(\gamma^{12}_{f1}+\theta^{12}_{gf}+\mu_{f1}+d_{f1})HY^{12}_{fl1}, \\ dHY^{12}_{fli}/dt &= d_{fi-1}HY^{12}_{fli-1}+\lambda^{12}_{fli}(HX_{fli}+HS_{fli})+\sum_h \lambda^h_{fli}HY^{3-h}_{fli} \\ &\quad +\Delta_{fi}\sum_b Y^{12}_{flib}-(\gamma^{12}_{fi}+\theta^{12}_{gf}+\mu_{fi}+d_{fi})HY^{12}_{fli}. \end{aligned}$$

### 3.6.3 Partially immune individuals $HZ$

The ODEs for category $HZ$ are

$$\begin{aligned} dHZ^h_{fl1}/dt &= \gamma^h_{f1}HY^h_{fl1}+\Delta_{f1}\sum_b Z^h_{fl1b}+\tau^h_{gf}(HGW^h_{fl1}+DHGW^h_{fl1}) \\ &\quad -(\lambda^{3-h}_{fl1}+\sigma^h_{zf1}+\mu_{f1}+d_{f1})HZ^h_{fl1}, \\ dHZ^h_{fli}/dt &= d_{fi-1}HZ^h_{fli-1}+\gamma^h_{fi}HY^h_{fli}+\Delta_{fi}\sum_b Z^h_{flib} \\ &\quad +\tau^h_{gf}(HGW^h_{fli}+DHGW^h_{fli})-(\lambda^{3-h}_{fli}+\sigma^h_{zfi}+\mu_{fi}+d_{fi})HZ^h_{fli}. \end{aligned}$$

The ODEs for $HZ^{12}$ are

$$\begin{aligned} dHZ^{12}_{fl1}/dt &= \tilde{\gamma}^{12}_{f1}\gamma^{12}_{f1}HY^{12}_{fl1}+\sum_h \gamma^h_{f1}HU^h_{fl1}+\Delta_{f1}(\sum_b Z^{12}_{fl1b}+\sum_s TCIN_{sl1}) \\ &\quad -(\sigma^{12}_{zf1}+\mu_{f1}+d_{f1})HZ^{12}_{fl1}, \\ dHZ^{12}_{fli}/dt &= d_{fi-1}HZ^{12}_{fli-1}+\tilde{\gamma}^{12}_{fi}\gamma^{12}_{fi}HY^{12}_{fli}+\sum_h \gamma^h_{fi}U^h_{flib} \\ &\quad +\Delta_{fi}(\sum_b Z^{12}_{flib}+\sum_s TCIN_{sli})-(\sigma^h_{zfi}+\mu_{fi}+d_{fi})HZ^{12}_{fli}. \end{aligned}$$

This material, provided by the authors as a supplement to Model for Assessing Human Papillomavirus Vaccination Strategies, is not part of Emerging Infectious Diseases contents.

### 3.6.4 Infected individuals with partial immunity $HU$

The ODEs for category $HU$ are

$$\begin{aligned} dHU^h_{fl1}/dt &= \lambda^h_{fl1} HZ^{3-h}_{fl1} + \tilde{\gamma}^{3-h}_{f1} \gamma^{12}_{f1} HY^{12}_{fl1} + \Delta_{f1} \sum_b U^h_{fl1b} \\ &\quad -(\gamma^h_{f1} + \theta^h_{gf} + \mu_{f1} + d_{f1}) HU^h_{fl1}, \\ dHU^h_{fli}/dt &= d_{fi-1} HU^h_{fli-1} + \lambda^h_{fli} HZ^{3-h}_{fli} + \tilde{\gamma}^{3-h}_{fi} \gamma^{12}_{fi} HY^{12}_{fli} \\ &\quad +\Delta_{fi} \sum_b U^h_{flib} - (\gamma^h_{fi} + \theta^h_{gf} + \mu_{fi} + d_{fi}) HU^h_{fli}. \end{aligned}$$

### 3.6.5 Vaccinated individuals $HV$

The ODEs for category $HV$ are

$$\begin{aligned} dHV_{fl1}/dt &= \Delta_{f1} \sum_b V^h_{fl1b} - (\sum_h \varphi^h_f \lambda^h_{fl1} + \sigma_{f1} + \mu_{f1} + d_{f1}) HV_{fl1}, \\ dHV_{fli}/dt &= d_{fi-1} HV_{fli-1} + \Delta_{fi} \sum_b V^h_{flib} - (\sum_h \varphi^h_f \lambda^h_{fli} + \sigma_{fi} + \mu_{fi} + d_{fi}) HV_{fli}. \end{aligned}$$

### 3.6.6 Vaccinated individuals with waned immunity $HS$

The ODEs for classes $HS$ are

$$\begin{aligned} dHS_{fl1}/dt &= \sigma_{f1} HV_{fl1} + \Delta_{f1} \sum_b S_{fl1b} - (\sum_h \lambda^h_{fl1} + \Delta_{f1} + \mu_{f1} + d_{f1}) HS_{fl1}, \\ dHS_{fli}/dt &= d_{fi-1} HS_{fli-1} + \sigma_{fi} HV_{fli} + \Delta_{fi} \sum_b S_{flib} - (\sum_h \lambda^h_{fli} + \mu_{fi} + d_{fi}) HS_{fli}. \end{aligned}$$

### 3.6.7 Infectious vaccinated individuals $HW$

The ODEs for category $HW$ are

$$\begin{aligned} dHW^h_{fl1}/dt &= \varphi^h_f \lambda^h_{fl1} HV_{fl1} + \Delta_{f1} \sum_b W^h_{fl1b} \\ &\quad -(\varphi^{3-h}_f \lambda^{3-h}_{fl1} + \alpha^h_{f1} \gamma^h_{f1} + \theta^h_{gfw} + \mu_{f1} + d_{f1}) HW^h_{fl1}, \\ dHW^h_{fli}/dt &= d_{fi-1} HW^h_{fli-1} + \varphi^h_f \lambda^h_{fli} HV_{fli} + \Delta_{fi} \sum_b W^h_{flib} \\ &\quad -(\varphi^{3-h}_f \lambda^{3-h}_{fli} + \alpha^h_{fi} \gamma^h_{fi} + \theta^h_{gfw} + \mu_{fi} + d_{fi}) HW^h_{fli}, \end{aligned}$$

This material, provided by the authors as a supplement to Model for Assessing Human Papillomavirus Vaccination Strategies, is not part of Emerging Infectious Diseases contents.

The ODEs for $HW^{12}$ are

$$\begin{aligned} dHW^{12}_{fl1}/dt &= \varphi^1_f\varphi^2_f\lambda^{12}_{fl1}HV_{fl1} + \sum_h \varphi^h_f\lambda^h_{fl1}HW^{3-h}_{fl1} + \Delta_{f1}\sum_b W^{12}_{fl1b} \\ &\quad -(\alpha^{12}_{f1}\gamma^{12}_{f1} + \theta^{12}_{gwf} + \mu_{f1} + d_{f1})HW^{12}_{fl1}, \\ dHW^{12}_{fli}/dt &= d_{fi-1}HW^{12}_{fli-1} + \varphi^1_f\varphi^2_f\lambda^{12}_{fli}HV_{fli} + \sum_h \varphi^h_f\lambda^h_{fli}HW^{3-h}_{fli} \\ &\quad +\Delta_{fi}\sum_b W^{12}_{flib} - (\alpha^{12}_{fi}\gamma^{12}_{fi} + \theta^{12}_{gwf} + \mu_{fi} + d_{fi})HW^{12}_{fli}. \end{aligned}$$

### 3.6.8 Vaccinated, partially immune individuals $HQ$

The ODEs for category $HQ$ are

$$\begin{aligned} dHQ^h_{fl1}/dt &= \alpha^h_{f1}\gamma^h_{f1}HW^h_{fl1} + \Delta_{f1}\sum_b Q^h_{fl1b} \\ &\quad -(\varphi^{3-h}_f\lambda^{3-h}_{fl1} + \mu_{f1} + d_{f1})HQ^h_{fl1}, \\ dHQ^h_{fli}/dt &= d_{fi-1}HQ^h_{fli-1} + \alpha^h_{fi}\gamma^h_{fi}HW^h_{fli} + \Delta_{fi}\sum_b Q^h_{flib} \\ &\quad -(\varphi^{3-h}_f\lambda^{3-h}_{fli} + \mu_{fi} + d_{fi})HQ^h_{fli}, \end{aligned}$$

The ODEs for $HQ^{12}$ are

$$\begin{aligned} dHQ^{12}_{fl1}/dt &= \alpha^{12}_{f1}\tilde{\gamma}^{12}_{f1}\gamma^{12}_{f1}HW^{12}_{fl1} + \sum_h \gamma^h_{f1}HP^h_{fl1} + \Delta_{f1}\sum_b Q^{12}_{fl1b} \\ &\quad -(\mu_{f1} + d_{f1})HQ^{12}_{fli}, \\ dHQ^{12}_{fli}/dt &= d_{fi-1}HQ^{12}_{fli-1} + \alpha^{12}_{fi}\tilde{\gamma}^{12}_{fi}\gamma^{12}_{fi}HW^{12}_{fli} + \sum_h \gamma^h_{fi}HP^h_{fli} + \Delta_{fi}\sum_b Q^{12}_{flib} \\ &\quad -(\mu_{fi} + d_{fi})HQ^{12}_{fli}. \end{aligned}$$

### 3.6.9 Vaccinated, infected individuals with partial immunity $HP$

The ODEs for category $HP$ are

$$\begin{aligned} dHP^h_{fl1}/dt &= \varphi^h_f\lambda^h_{fl1}HQ^{3-h}_{fl1} + \alpha^{3-h}_{f1}\tilde{\gamma}^{3-h}_{f1}HW^{12}_{fl1} \\ &\quad +\Delta_{f1}\sum_b P^h_{fl1b} - (\alpha^h_{f1}\gamma^h_{f1} + \theta^h_{gwf} + \mu_{f1} + d_{f1})HP^h_{fl1}, \\ dHP^h_{fli}/dt &= d_{fi-1}HP^h_{fli-1} + \varphi^h_f\lambda^h_{fli}HQ^{3-h}_{fli} + \alpha^{3-h}_{fi}\tilde{\gamma}^{3-h}_{fi}HW^{12}_{fli} \\ &\quad +\Delta_{fi}\sum_b P^h_{flib} - (\alpha^h_{fi}\gamma^h_{fi} + \theta^h_{gwf} + \mu_{fi} + d_{fi})HP^h_{flib}. \end{aligned}$$

This material, provided by the authors as a supplement to Model for Assessing Human Papillomavirus Vaccination Strategies, is not part of Emerging Infectious Diseases contents.

### 3.6.10 Genital warts $GW$

The genital warts class consists of the following differential equations

$$
\begin{aligned}
dHGW^2_{kl1b}/dt &= \theta_{gs}(\theta^2_{gk}(HY^2_{kl1b} + HU^2_{kl1b}) + \theta^{12}_{gk}HY^{12}_{kl1b} \\
&\quad + \theta^2_{gwk}(HW^2_{kl1b} + HP^2_{kl1b}) + \theta^{12}_{gwk}HW^{12}_{kl1b}) + \Delta_{k1}GW^2_{klib} \\
&\quad - (\tau^2_{gk} + \mu_{k1} + d_{k1})HGW^2_{kl1b}, \\
dHGW^2_{klib}/dt &= d_{ki-1}HGW^2_{kli-1b} + \theta_{gs}(\theta^2_{gk}(HY^2_{klib} + HU^2_{klib}) + \theta^{12}_{gk}HY^{12}_{klib} \\
&\quad + \theta^2_{gwk}(HW^2_{klib} + HP^2_{klib}) + \theta^{12}_{gwk}HW^{12}_{klib}) + \Delta_{ki}GW^2_{klib} \\
&\quad - (\tau^2_{gk} + \mu_{ki} + d_{ki})HGW^2_{kl1b},
\end{aligned}
$$

$$
\begin{aligned}
dDHGW^2_{kl1b}/dt &= (1 - \theta_{gs})(\theta^2_{gk}(HY^2_{kl1b} + HU^2_{kl1b}) + \theta^{12}_{gk}HY^{12}_{kl1b} \\
&\quad + \theta^2_{gwk}(HW^2_{kl1b} + HP^2_{kl1b}) + \theta^{12}_{gwk}HW^{12}_{kl1b}) + \Delta_{k1}HGW^2_{kl1b} \\
&\quad - (\tau^2_{gk} + \mu_{k1} + d_{k1})DHGW^2_{kl1b}, \\
dDHGW^2_{klib}/dt &= d_{ki-1}HGW^2_{kli-1b} + (1 - \theta_{gs})(\theta^2_{gk}(HY^2_{klib} + HU^2_{klib}) \\
&\quad + \theta^{12}_{gk}HY^{12}_{klib} + \theta^2_{gwk}(HW^2_{klib} + HP^2_{klib}) + \theta^{12}_{gwk}HW^{12}_{klib}) \\
&\quad + \Delta_{ki}HGW^2_{klib} - (\tau^2_{gk} + \mu_{ki} + d_{ki})DGW^2_{klib}.
\end{aligned}
$$

## 3.7 Forces of HPV infection $\lambda$

The rate at which susceptible individuals acquire infection with type $h$ (per capita force of infection) $\lambda^h_{kli}$ is gender, sexual activity, age, and time dependent. The rate $\lambda^h_{kli}$ at which individuals of gender $k$, sexual activity group $l$, age class $i$, at time $t$ acquire infection with type $h$ depends on the number of gender partnerships and the way they form partnerships with individuals of the opposite gender $k'$, the fraction of infected sex partners, and the transmission probability $\beta^h_k$ per partnership. The force of HPV infection $\lambda^h_{kli}$ is given by

$$
\begin{aligned}
\lambda^h_{mli} &= \beta^h_m \sum_{j=1}^{17}\sum_{a=1}^{3} c_{mlaij}\rho_{mlaij}\Bigg(\sum_{b=1}^{2}[r_f(W^h_{fajb} + P^h_{fajb} + W^{12}_{fajb}) + Y^h_{fajb} \\
&\quad + Y^{12}_{fajb} + U^h_{fajb} + \sum\nolimits_s^3 \left(CIN^h_{sajb} + DCIN^h_{sajb} + ICIN^h_{sajb}\right) + GW^h_{fajb} \\
&\quad + DGW^h_{fajb} + \sum_{s}^{L,R,D} CC^h_{sajb}] + \sum\nolimits_s^2 \left(CIS^h_{sajb} + DCIS^h_{sajb} + ICIS^h_{sajb}\right)] \\
&\quad + r_f(HP^h_{faj} + HW^h_{faj} + HW^{12}_{faj}) + HU^h_{faj} + HY^{12}_{faj} \\
&\quad + HGW^h_{faj} + DHGW^h_{faj}\Big) / N_{faj},
\end{aligned}
$$

$$
\begin{aligned}
\lambda^h_{fli} &= \beta^h_f \sum_{j=1}^{17}\sum_{a=1}^{3} c_{flaij}\rho_{flaij}\left(Y^h_{maj} + U^h_{maj} + Y^{12}_{maj} + GW^h_{maj} + DGW^h_{maj}\right. \\
&\quad \left. + r_m\left(W^h_{maj} + W^{12}_{maj} + P^h_{maj}\right)\right) / N_{maj},
\end{aligned}
$$

$h = 1, 2$. Coinfection occurs at rate

$$\lambda_{mli}^{12} = \beta_m^1 \beta_m^2 \sum_{j=1}^{17} \sum_{a=1}^{3} c_{mlaij} \rho_{mlaij} \times \left( HY_{faj}^{12} + r_f HW_{faj}^{12} + \sum_{b=1}^{2} (Y_{fajb}^{12} + r_f W_{fajb}^{12}) \right) / N_{faj},$$

$$\lambda_{fli}^{12} = \beta_f^1 \beta_f^2 \sum_{j=1}^{17} \sum_{a=1}^{3} c_{flaij} \rho_{flaij} \left( Y_{maj}^{12} + r_m W_{maj}^{12} \right) / N_{maj}.$$

## 3.8 Mixing preferences

### 3.8.1 Mixing matrix $\rho$

The way sex partnerships are formed is governed by the conditional probability matrix $\rho$. Thus, $\rho_{klmij}$ is the probability of someone of gender $k$, sexual activity group $l$, age class $i$ having a partner from the opposite gender from sexual activity group $m$ and age class $j$. This depends on the proportion of sex partners from the opposite gender from sexual activity group $m$ and age class $j$, $c_{k'mj} N_{k'mj}(0)$, in the total sexually active population. In generating the mixing matrix $\rho$, the parameters $\epsilon_1$ and $\epsilon_2$ are used to depict the degree of assortative mixing between age and sexual activity groups, respectively. Thus, mixing is fully assortative ($\rho$ is the identity matrix $\rho_{klmij} = \delta_{lm} \delta_{ij}$, where $\delta_{ij}$ is the Kronecker delta) if $\epsilon_1 = \epsilon_2 = 0$ and proportionate when $\epsilon_1 = \epsilon_2 = 1$ [24, 25, 26, 27]. The mixing matrix $\rho_{klmij}$ is given by

$$\rho_{klmij} = \left( (1 - \epsilon_1) \delta_{ij} + \epsilon_1 \frac{\sum_{s=1}^{3} c_{k'sj} N_{k'sj}(0)}{\sum_{u=1}^{17} \sum_{a=1}^{3} c_{k'au} N_{k'au}(0)} \right) \times \left( (1 - \epsilon_2) \delta_{lm} + \epsilon_2 \frac{\sum_{u=1}^{17} c_{k'mu} N_{k'mu}(0)}{\sum_{u=1}^{17} \sum_{a=1}^{3} c_{k'au} N_{k'au}(0)} \right).$$

The model should satisfy the constraints balancing the supply of and demand for sexual partnerships: $c_{klmij} \rho_{klmij} N_{kli} = c_{k'mlji} \rho_{k'mlji} N_{k'mj}$. This is accomplished by specifying the mean rates of sex partner change as functions of the initial imbalance in the supply and demand of sex partnerships. Thus,

$$c_{klmij} = c_{kli} B_{lmij}^{0.5},$$

where

$$B_{lmij} = \frac{c_{k'mj} \rho_{k'mlji} N_{k'mj}(0)}{c_{kli} \rho_{klmij} N_{kli}(0)}.$$

The differential effects of cervical cancer-induced mortality are also likely to cause an imbalance between the demand for and supply of sex partnerships. There are few options for rectifying this. One option is to let the rates of sex

This material, provided by the authors as a supplement to Model for Assessing Human Papillomavirus Vaccination Strategies, is not part of Emerging Infectious Diseases contents.

partner change and mixing pattern of one gender vary over time so as to satisfy the above constraints. Another option is to fix the mixing patterns of both sexes and to let their rates of sex partner change vary over time so as to balance the supply of and demand for sex partnerships [25]. However, this latter option requires adding additional differential equations that may considerably increase the size of the model. Because of this additional complexity only the former option is tried. Thus,

$$c_{k'mlji}(t) = \frac{c_{klmij}\rho_{klmij}N_{kli}(t)}{\rho_{k'mlji}N_{k'mj}(t)}.$$

In the sensitivity analysis, the gender that will be chosen first will be varied to test the robustness of the results.

### 3.8.2 Estimates of the mixing matrix

Even though the crucial role of the mixing matrix in the spread of many sexually transmitted infections has been repeatedly emphasized before [24, 25, 26, 27], there are no adequate data to generate such a matrix. The current analysis follows previous work in this area by examining the range of patterns that are likely to arise in practice. This range is governed by the parameters $\epsilon_1$ and $\epsilon_2$ whose respective values are set to 0.6 and 0.7 in the baseline analysis and varied over a wide range in the sensitivity analysis. These estimates are obtained from the National Health and Social Life Survey (NHSLS) [55, 63, 64]. Higher values for $\epsilon_2$ are reported for high-risk populations. For example, Garnett et al [26] estimated a value of 0.9 using data from a sample of patients with STD seen at the Harborview Medical Center. The baseline parameter values for the rate of sex partner change, stratified by gender, sexual activity, and age, are calculated from Table 3 using data from the NHSLS and the procedure outlined in Garnett and Anderson [24, 25]. Briefly, this procedure can be described as follows. Let the relative partner acquisition rate of sexual activity group $l$ relative to the lowest group be $pc_l$. Similarly, define the relative partner acquisition rate of age group $i$ relative to the lowest group as $pa_i$. Therefore, the rate of sex partner change for people in age group 18–59 is

$$c_{kli} = \frac{pc_l pa_i \bar{c}_3 \sum_{l=1}^{3}\sum_{j=3}^{11} N_{klj}(0)}{\sum_{l=1}^{3}\sum_{j=3}^{11} N_{klj}(0) pc_l pa_j},$$

where $\bar{c}_3$ is the weighted mean rate of sex partner change rate. The rates of sex partner change for the individuals in the age groups 12–14, 15–17, and over 60 years are calculated in a similar fashion. For individuals in the sexually active age groups 18–59, a value for $\bar{c}_3$ of 1.3 new partners per year was used in the analysis [55]. A value for $\bar{c}_1$of 0.1 and $\bar{c}_2$ of 0.3 new partners per year was used for individuals in age groups 12–14, and 15–17, respectively [1]. It is assumed

This material, provided by the authors as a supplement to Model for Assessing Human Papillomavirus Vaccination Strategies,
is not part of Emerging Infectious Diseases contents.

| Activity group | Proportion of population, % | | Relative partner acquisition rate | Reference |
|---|---|---|---|---|
| | males, $\omega_m$ | females, $\omega_f$ | (RPAR), $pc_l$ | |
| 1 (highest) | 2.56 | 2.56 | 11.29 | [55] |
| 2 | 11.47 | 11.47 | 2.96 | |
| 3 (lowest) | 85.97 | 85.97 | 1 | |
| **Age group** | **RPAR, $pa_i$** | **Mean partner acquisition rate, $\bar{c}_j$** | | |
| 12–14 | 0.11 | 0.1 | | [1] |
| 15–17 | 1.18 | 0.3 | | [1] |
| 18–19 | 2.42 | 1.3 | | [55] |
| 20–24 | 2.61 | | | |
| 25–29 | 2.55 | | | |
| 30–34 | 1.72 | | | |
| 35–39 | 1.65 | | | |
| 40–44 | 1.53 | | | |
| 45–49 | 1.38 | | | |
| 50–54 | 1.25 | | | |
| 55–59 | 1.00 | | | |
| 60 − 69 | 0.61 | 0.5 | | assumed |
| ≥ 70 | 0.44 | | | |
| | males | females | | |
| Population size, $N_k$ | 50,000 | 50,000 | | |

Table 3: Baseline behavioral parameter values for the sexually active population

This material, provided by the authors as a supplement to Model for Assessing Human Papillomavirus Vaccination Strategies, is not part of Emerging Infectious Diseases contents.

that for individuals 60 years and older $\bar{c}_4$ is 0.5. Other values were used in the sensitivity analysis.

## 3.9 Balancing population

To close the model, the total number of people in each gender category $k$, ($k = f, m$), age group $i$ ( $i$ = 1, 2, …,17) and sexual activity group $l$ ($l$ = 1, 2, 3) must be equal to the sum of individuals in each epidemiologic class in the respective gender, age, and sexual activity groups. That is,

$$\begin{aligned} N_{mli} &= \sum_{h=1}^{2} \left(Y^h_{mli} + Z^h_{mli} + U^h_{mli} + W^h_{mli} + Q^h_{mli} + P^h_{mli} + GW^h_{mli}\right) \\ &\quad + X_{mli} + V_{mli} + S_{mli} + Y^{12}_{mli} + Z^{12}_{mli} + W^{12}_{mli} + Q^{12}_{mli}, \end{aligned}$$

For females this requires

$$\begin{aligned} N_{fli} &= \sum_{b=1}^{2} \left( \sum_{h=1}^{2} [Y^h_{flib} + Z^h_{flib} + U^h_{flib} + W^h_{flib} + Q^h_{flib} + P^h_{flib} + GW^h_{flib} \right. \\ &\quad + \sum_{s=1}^{3} (CIN^h_{slib} + DCIN^h_{slib} + ICIN^h_{slib}) + \sum_{s=1}^{2} (CIS^h_{slib} + DCIS^h_{slib} \\ &\quad + ICIS^h_{slib}) + \sum_{s}^{L,R,D} CC^h_{slib}] + X_{flib} + V_{flib} + S_{flib} + Y^{12}_{flib} \\ &\quad \left. + Z^{12}_{flib} + W^{12}_{flib} + Q^{12}_{flib} \right) + \sum_{s=1}^{3} TCIN_{sli} + \sum_{s=1}^{2} TCIS_{sli} \sum_{s}^{L,R,D} DCC_{sli} \\ &\quad + \sum_{h=1}^{2} \left(HY^h_{fli} + HZ^h_{fli} + HU^h_{fli} + HW^h_{fli} + HQ^h_{fli} + HP^h_{fli} + HGW^h_{fli}\right) \\ &\quad + HX_{fli} + HV_{fli} + HS_{fli} + HY^{12}_{fli} + HZ^{12}_{fli} + HQ^{12}_{fli} + HW^{12}_{fli} + SCC_{li}. \end{aligned}$$

As evident from the system of equations described above, the demographic model, the HPV model, the cancer model, and the genital warts model are fully integrated, and can only be solved together. The total number of differential equations in the entire model is 7191.

## 3.10 Estimates of epidemiologic parameters

A comprehensive search of the literature was conducted in order to obtain baseline values for the natural history and clinical parameters.

### 3.10.1 Estimates of natural history parameters

The values of natural history parameters are reported in Tables 4–5. The way these estimates were derived is explained elsewhere [46].

This material, provided by the authors as a supplement to Model for Assessing Human Papillomavirus Vaccination Strategies, is not part of Emerging Infectious Diseases contents.

| Parameter | Estimate | Reference |
|---|---|---|
| mean duration of acute HPV infection , years | | [29] |
| HPV 16/18, $1/(\gamma^1_{ki} + \sum_s \theta^1_{ks})$ | 1.2 | |
| HPV 6/11, $1/(\gamma^2_{ki} + \theta^2_{kg} + \sum_s \theta^2_{ks})$ | 0.7 | |
| progression in the presence of HPV 16/18 per year, % | | |
| Normal to CIN1, $\theta^1_{k1}$ | 9.4 | [38] |
| Normal to CIN2, $\theta^1_{k2}$ | 5.8 | [84] |
| Normal to CIN3, $\theta^1_{k3}$ | 5.3 | [84] |
| CIN1 to CIN2, $\pi^1_{1i}$ | 13.6 | [42] |
| CIN2 to CIN3, $\pi^1_{2i}$ | 14 | [48, 16] |
| CIN3 to CIS1, $\pi^1_{3i}$ | 42 | [48, 80] |
| CIS1 to CIS2, $\pi^1_{4i}$ | 5 | |
| CIS2 to LCC, $\pi^1_{5i}$ | 18 | |
| LCC to RCC, $\pi^1_{Li}$ | 10 | [32, 71, 61] |
| RCC to DCC, $\pi^1_{Ri}$ | 30 | [61] |
| progression in the presence of HPV 6/11 HPV per year, % | | |
| Normal to CIN1, $\theta^2_{k1}$ | 9.5 | [43] |
| Normal to CIN2, $\theta^2_{k2}$ | 1.9 | [43, 3, 20, 40, 68] |
| CIN1 to CIN2, $\pi^2_{1i}$ | 0 | [43, 3, 20, 40, 68] |
| Normal to genital warts, $\theta^2_{kg}$ | 57 | [84] |
| regression in the presence of HPV 16/18 per year, % | | |
| CIN1 to normal/HPV, $\tau^1_{f1}$ | 32.9 | [43, 72] |
| CIN2 to normal/HPV, $\tau^1_{f2}$ | 31 | [48, 16, 57] |
| CIN2 to CIN1, $\tau^1_{f21}$ | 13.3 | [16] |
| CIN3 to normal/HPV, $\tau^1_{f3}$ | 11 | [48] |
| CIN3 to CIN1, $\tau^1_{f31}$ | 3 | [48, 16] |
| CIN3 to CIN2, $\tau^1_{f32}$ | 3 | [48, 16] |
| regression in the presence of HPV 6/11 HPV per year, % | | |
| CIN1, $\tau^2_{f1}$ | 55.2 | [43] |
| genital warts, $\tau^2_{gk}$ | 87.5 | [84] |
| hysterectomy rate, $\Delta_i$, % | | [49] |
| 15–24 years | 0.02 | |
| 25–29 years | 0.26 | |
| 30–34 years | 0.53 | |
| 35–39 years | 0.89 | |
| 40–44 years | 1.17 | |
| 45–54 years | 0.99 | |
| $\geq$ 55 years | 0.36 | |

Table 4: Baseline biological parameter values for the HPV and disease compartments and hysterectomy

This material, provided by the authors as a supplement to Model for Assessing Human Papillomavirus Vaccination Strategies, is not part of Emerging Infectious Diseases contents.

| Parameter | Estimate | Reference |
|---|---|---|
| age-specific cervical cancer mortality rates, % per year | | [75] |
| for LCC, $\chi_L$ | | |
| 15–29 years | 0.7 | |
| 30–39 years | 0.6 | |
| 40–49 years | 0.8 | |
| 50–59 years | 1.9 | |
| 60–69 years | 4.2 | |
| $\geq$ 70 years | 11.6 | |
| for RCC, $\chi_R$ | | |
| 15–29 years | 13.4 | |
| 30–39 years | 8.9 | |
| 40–49 years | 11.0 | |
| 50–59 years | 10.1 | |
| 60–69 years | 17.6 | |
| $\geq$ 70 years | 28.6 | |
| for DCC, $\chi_D$ | | |
| 15–29 years | 42.9 | |
| 30–39 years | 41.0 | |
| 40–49 years | 46.7 | |
| 50–59 years | 52.7 | |
| 60–69 years | 54.6 | |
| $\geq$ 70 years | 70.3 | |

Table 5: Annual age-specific cervical cancer mortality rates, 1997–2002

#### 3.10.2 Estimates of other clinical parameters

The values of screening, diagnosis, and treatment parameters are reported in Tables 6.

#### 3.10.3 Estimates of vaccine parameters

The efficacy of the vaccine against incident infection (HPV 6/11 or 16/18) was assumed to be 90%. It was also assumed that infected vaccinated individuals do not progress to disease [52, 78]. We assumed the vaccine does not affect the natural course of disease. The duration of immunity conferred by vaccination is currently unknown. We assumed the duration of protection of HPV vaccination to be lifelong for the base case as was done in previous models [32] and examined a duration of 10 years in sensitivity analyses. Given HPV vaccination coverage is unknown, we assumed that 70% of adolescents will receive a 3-dose vaccine before they turn 12 similar to the coverage rates used in previous models [71, 32]. Coverage was also assumed to increase linearly from 0% up to 70% during the first five years of the program and remain at 70% thereafter. We assumed that vaccine coverage for the catch-up program would increase linearly from 0% up to 50% during the first 5 years and then drop to 0% after 5 years.

# 4 Epidemiologic impact of screening and vaccination strategies

To assess the epidemiologic impact of each vaccination strategy several intermediate and two final outcome measures of effectiveness were chosen. Examples of some of the intermediate outcome are shown in Figures **??**–**??** and discussed below.

## 4.1 Years of life

The first final outcome measure is the total number of years spent alive by the active population. Thus, the discounted total number of years of life achieved using strategy $a$ is given by

$$YL_a = \int_0^T \left( \sum_{k\in\{f,m\}} \sum_{l=1}^{3} \sum_{i=1}^{17} N_{kli} \right) e^{-\xi t} dt$$

where $N_{kli}$ is the size of the population of gender $k$, in sexual activity group $l$, and in age group $i$; $\xi$ is the discount rate; and $T$ is the planning horizon.

## 4.2 Quality-adjusted life years

The second final measure of effectiveness assigns quality of life weights to each health state and integrates the sum of all these quality-adjusted health states

This material, provided by the authors as a supplement to Model for Assessing Human Papillomavirus Vaccination Strategies, is not part of Emerging Infectious Diseases contents.

| Parameter | Estimate | Reference |
|---|---|---|
| Routine cervical screening, $cover_i$, % per year | | [44] |
| 10–14 years | 0.6 | |
| 15–19 years | 21.0 | |
| 20–24 years | 44.8 | |
| 25–29 years | 61.6 | |
| 30–34 years | 54.9 | |
| 35–39 years | 50.5 | |
| 40–44 years | 48.1 | |
| 45–49 years | 49.1 | |
| 50–54 years | 51.1 | |
| 55–59 years | 46.7 | |
| 60–64 years | 42.5 | |
| 65–69 years | 38.9 | |
| 70–74 years | 29.6 | |
| 75-79 years | 20.1 | |
| 80-84 years | 11.1 | |
| 85+ | 5.5 | |
| Females never screened, $\varrho_1$ | 5 | |
| Liquid-based cytology sensitivity, $papsn_s$, % | | |
| for CIN1 | 28 | [7] |
| for $\geq$ CIN2/3 | 59 | [7] |
| Liquid-based cytology specificity, $papsp$, % | 94 | [7, 14] |
| Colposcopy sensitivity, $colpsn$, % | 96 | [60] |
| Colposcopy specificity, $colpsp$, % | 48 | [60] |
| Genital wart patients seeking physician care, $1 - \theta_{gs}$, % | 75 | [12] |
| Symptoms recognition, % | | |
| LCC, $recog_L$ | 3.8 | |
| RCC, $recog_R$ | 18 | |
| DCC, $recog_d$ | 90 | |
| Cure rate with treatment per year, % | | |
| for CIN1, $cure_1$ | 96 | [22] |
| for CIN2, $\Gamma_2$ | 92 | [22] |
| for CIN3, $\Gamma_3$ | 92 | [22] |
| for LCC, $\Omega_L$ | 92 | [69] |
| for RCC, $\Omega_R$ | 53 | [69] |
| for DCC, $\Omega_D$ | 17 | |
| Persistence of HPV after treatment for CIN, % | 34 | [15] |

Table 6: Cervical cytology screening and colposcopy characteristics and rates of cure and symptom recognition

over the planning horizon $(0, T)$. Let $qcin_s$, $qcis_s$, $qcc_s$, $qccs$, $qgw_k$, and $q_{ki}$ denote the quality of life weights for an individual in the detected health state CIN stage $s$, CIS stage $s$, cervical cancer stage $s$, genital warts, and normal of gender $k$ at age $i$; respectively. The discounted total number of quality-adjusted life years using strategy $a$ over the planning horizon $(0, T)$ is given by

$$QALY_a = \int_0^T e^{-\xi t} \left\{ \sum_{l=1}^{3} \sum_{i=1}^{17} q_{mi} \left( N_{mli} - (1 - qgw_m) DGW_{mli}^2 \right) \right.$$
$$+ q_{fi} \left[ N_{fli} - (1 - qgw_f)(DHGW_{fli}^2 + \sum_{b=1}^{2} DGW_{flib}^2) - (1 - qccs) SCC_{li} \right.$$
$$- \sum_{h=1}^{2} \left( \sum_{s=1}^{3} (1 - qcin_s) \sum_{b=1}^{2} DCIN_{slib}^h + \sum_{s=1}^{2} (1 - qcis_s) \sum_{b=1}^{2} DCIS_{slib}^h \right)$$
$$\left. \left. - \left( \sum_{s=L,R,D} (1 - qcc_s) DCC_{sli} \right) \right] \right\} dt.$$

Note that the quality-adjusted years of life for females are reduced by time spent in diagnosed genital warts, CIN, and cancer states $DCIN_s$, $DCC_s$, $DGW$, and $SCC$. Males' quality of life deteriorates by spending time with detected genital warts. The probability of genital warts being recognized and treated is assumed to be 75%. It is assumed here that if a persons's health condition is not detected, the quality of life of that person will be the same as that of a person without the condition. This assumption biases the results against the vaccine. In the sensitivity analysis, the magnitude of the quality of life improvements for persons with undetected conditions prevented by the vaccine will be quantified.

## 4.3 Estimates of quality of life weights

Women diagnosed with CIN1 and CIN2/3 were assumed to have quality weight of 0.91 and 0.87, respectively [62, 54]. The quality weight for genital warts is assumed to be 0.91 [62]. Females with local and regional cancer are assumed to have a quality of life weight of 0.76 and 0.67, respectively [62]. A quality weight for invasive distant cancer of 0.48 was derived from Gold et al [30] using the 25th percentiles of female genital cancer weights. It is assumed that the quality of life for cervical cancer survivors after successful treatment will continue to be lower (at 0.76) than that of healthy females [4, 83]. Undiagnosed HPV, genital warts, CIN, and cervical cancer states and successfully treated CIN states are assumed to have a quality of life weight similar to those of individuals without HPV disease. Gender- and age-specific quality weights for other health states were derived from Gold [30]. Similar values were reported from the Beaver Dam Health Outcomes study [23]. CIN and cancer health states were multiplied by the age- and gender-specific weights to reflect the variation in quality of life by age and gender groups.

This material, provided by the authors as a supplement to Model for Assessing Human Papillomavirus Vaccination Strategies, is not part of Emerging Infectious Diseases contents.

| Condition | Estimate | | Reference |
|---|---|---|---|
| | females | males | |
| genital warts, $qgw_k$ | 0.91 | 0.91 | [62] |
| CIN1, $qcin_1$ | 0.91 | | [62] |
| CIN2, $qcin_2$ | 0.87 | | [62] |
| CIN3, $qcin_3$ | 0.87 | | [62] |
| CIS, $qcis_s$ | 0.87 | | [62] |
| EICC, $qcc_L$ | 0.76 | | [62] |
| RLICC, $qcc_R$ | 0.67 | | [62] |
| DLICC, $qcc_D$ | 0.48 | | [30] |
| Cancer survivors, $qccs$ | 0.76 | | [83] |
| No condition, $q_{ki}$ | | | |
| 12–17 years | 0.93 | 0.93 | [30] |
| 18–34 years | 0.91 | 0.92 | [30] |
| 35–44 years | 0.89 | 0.90 | [30] |
| 45–54 years | 0.86 | 0.87 | [30] |
| 55–64 years | 0.80 | 0.81 | [30] |
| 65–74 years | 0.78 | 0.76 | [30] |
| $\geq$ 75 years | 0.70 | 0.69 | [30] |

Table 7: Quality of life weights

# 5 Economic consequences of screening and vaccination strategies

The total costs of each strategy includes costs of cytology screening per unit time, cost of vaccination, lifetime cost of treating detected genital warts, CIN and invasive cancer cases, and the cost of following false positive results of screening.

## 5.1 Screening costs

The cost of cytology screening per unit time is the product of the cost per test $scn$, the test compliance rate $cover_{ib}$ given the frequency of administering the test per unit time (e.g., every year), and the size of the population eligible for screening $\sum_l \sum_i \{\sum_b (X_{flib}+V_{flib}+S_{flib}+Y^{12}_{flib}+Z^{12}_{flib}+W^{12}_{flib}+Q^{12}_{flib}+\sum_h [Y^h_{flib}+Z^h_{flib}+U^h_{flib}+W^h_{flib}+Q^h_{flib}+P^h_{flib}+GW^h_{flib}+\sum_s CIN^h_{slib}+\sum_s CIS^h_{slib}+\sum_s CC^h_{slib}]$. For simplicity, it is assumed that females in the hysterectomy class are not screened. However, this may not be the case as suggested by recent studies [70]. The cost of following false positive results of the cytology test is the product of the cost of colposcopy $colp$ of those females who do not have a *repeat* cytology test, one minus cytology specificity $papsp$ and the size of the screened population that is truly negative $\sum_l \sum_i \{\sum_b (X_{flib}+V_{flib}+S_{flib}+Y^{12}_{flib}+Z^{12}_{flib}+W^{12}_{flib}+Q^{12}_{flib}+\sum_h [Y^h_{flib}+Z^h_{flib}+U^h_{flib}+W^h_{flib}+Q^h_{flib}+P^h_{flib}+GW^h_{flib}]$. Since

This material, provided by the authors as a supplement to Model for Assessing Human Papillomavirus Vaccination Strategies, is not part of Emerging Infectious Diseases contents.

colposcopy is not 100% specific, to this it should be added the cost of a false positive colposcopy result. This, in turn, equals the product of the cost of biopsy *biopsy*, one minus colposcopy specificity *colsp* and the size of the screened population that has false cytology results. We also assumed that females in categories $TCIN_s, ICIN_s$, $ICIN_s$, and $SCC$ receive annual Pap tests, some of which will be false positives resulting in additional colposcopies and biopsies. Total screening costs associated with strategy $a$ at time $t$ are

$$\begin{aligned}
&Screen_a(t) = \\
&scn \times \{\sum_l \sum_i (\sum_b cover_{ib} \times (X_{flib} + V_{flib} + S_{flib} + Y^{12}_{flib} + Z^{12}_{flib} + W^{12}_{flib} \\
&+Q^{12}_{flib} + \sum_h [Y^h_{flib} + Z^h_{flib} + U^h_{flib} + W^h_{flib} + Q^h_{flib} + P^h_{flib} + GW^h_{flib} \\
&+\sum_s CIN^h_{slib} + \sum_s CIS^h_{slib} + \sum_s CC^h_{slib})]\} + \\
&(1 - papsp) \times [repeat \times scn + (1 - repeat)(colp + biopsy \times (1 - colpsp))] \\
&\times\{\sum_l \sum_i [\sum_b cover_{ib} \times (X_{flib} + V_{flib} + S_{flib} + Y^{12}_{flib} + Z^{12}_{flib} + W^{12}_{flib} \\
&+Q^{12}_{flib} + \sum_h [Y^h_{flib} + Z^h_{flib} + U^h_{flib} + W^h_{flib} + GW^h_{flib} + Q^h_{flib} + P^h_{flib})]\} \\
&+\{scn + (1 - papsp) \times [colp + biopsy \times (1 - colpsp)]\} \\
&\times\{\sum_l \sum_i [SCC_{li} + \sum_s TCIN_{sli} + \sum_h \sum_b (\sum_s ICIN^h_{slib} + \sum_s ICIS^h_{slib})]\}.
\end{aligned}$$

## 5.2 Treatment costs

Treatment costs of genital warts, CIN, and cancer cases are the product of the number of cases detected and treated and the cost of treatment. Cases of genital warts occur at rate $(1-\theta_{gs}) \sum_k \{\theta^2_{gk}[HY^2_{kli} + HU^2_{kli} + \sum_b (Y^2_{klib} + U^h_{klib})] + \theta^{12}_{gk}(HY^{12}_{kli} + \sum_b Y^{12}_{klib}) + \theta^2_{gwk}[HW^2_{kli} + HP^2_{kli} + \sum_b (W^2_{klib} + P^h_{klib})] + \theta^{12}_{gwk}(HW^{12}_{kli} + \sum_b W^{12}_{klib})\}$ at a cost of $cgw_k$ per case. Because it is assumed that the rate of treatment for diagnosed CIN is $\Gamma_s$ and all cancer cases are treated, the number of cases treated at time $t$ is the total number of treated CIN and cancer detected $\sum_h \sum_l \sum_i \sum_b [\sum_s \Gamma_s DCIN^h_{slib} + \sum_s \Gamma_{3+s} DCIS^h_{slib} + \sum_s \upsilon_{sib} CC^h_{slib})]$. The cost of treating CIN and cancer at stage $s$ is denoted by $ctcin_s$ and $ctcc_s$, respectively.

Thus, total treatment costs at time $t$ if strategy $a$ is adopted is:

$$Treat_a(t) =$$
$$\sum_l \sum_i \sum_k cgw_k(1-\theta_{gs})\{\theta^2_{gk}[HY^2_{kli} + HU^2_{kli} + \sum_b (Y^2_{klib} + U^2_{klib})]$$
$$+\theta^{12}_{gk}(HY^{12}_{kli} + \sum_b Y^{12}_{klib}) + \theta^2_{gwk}[HW^2_{kli} + HP^2_{kli}$$
$$+\sum_b (W^2_{klib} + P^2_{klib})] + \theta^{12}_{gwk}(HW^{12}_{kli} + \sum_b W^{12}_{klib})\} + \sum_h \sum_l \sum_i \sum_b$$
$$\left(\sum_s ctcin_s(\Gamma_s DCIN^h_{slib} + \Gamma_{3+s} DCIS^h_{slib}) + \sum_s (ctcc_s \times \upsilon_{sib} CC^h_{slib})\right).$$

## 5.3 Vaccination costs

Total vaccination costs at time $t$ include the cost of the vaccine and the number of people vaccinated $\sum_k \sum_l \sum_b \{B_{klb}\phi_{kl0b} + \sum_i \phi_{klib}[Y^{12}_{klib} + Z^{12}_{klib} + \sum_h (X^h_{klib} + Y^h_{klib} + Z^h_{klib} + U^h_{klib} + GW^h_{klib} + \sum_s (CIN^h_{slib} + CIS^h_{slib} + CC^h_{slib}))]\}$. Thus, total vaccination costs at time $t$ associated with strategy $a$ are:

$$\begin{aligned} Vaccinate_a(t) &= vaccine \times \sum_l \sum_k \sum_b \{B_{klb}\phi_{kl0b} + \sum_i \phi_{klib}[Y^{12}_{klib} + Z^{12}_{klib} \\ &\quad + \sum_h (X^h_{klib} + Y^h_{klib} + Z^h_{klib} + U^h_{klib} + GW^h_{klib} \\ &\quad + \sum_s (CIN^h_{slib} + CIS^h_{slib} + CC^h_{slib}))]\}. \end{aligned}$$

## 5.4 Total costs

Discounted total cost over the planning horizon $(0, T)$ of following strategy $a$ is

$$Cost_a = \int_0^T [Screen_a(t) + Treat_a(t) + Vaccinate_a(t)]\, e^{-\xi t} dt.$$

## 5.5 Estimates of costs

Direct medical costs for screening and diagnosis were estimated from the 2001 Medstat Marketscan® commercial insurance database [56] and updated to 2005 dollar values by using the medical care component of the U.S. consumer price index [77]. The direct medical costs in 2005 of liquid-based cytology were estimated at \$99. The cost of colposcopy was \$165 and colposcopy with cervical biopsy at the same visit was \$318. The direct medical costs of treatment of CIN and cervical cancer were based on the results of Kim et al [50] and updated to 2005 dollar values [77]. The costs of CIN 1 were \$1554, CIN 2/3 \$3483, local invasive cervical cancer \$26,470, regional invasive cervical cancer \$28,330, and

This material, provided by the authors as a supplement to Model for Assessing Human Papillomavirus Vaccination Strategies, is not part of Emerging Infectious Diseases contents.

| Condition | Estimate females | males | Reference |
|---|---|---|---|
| cytology test, *scn* | \$99 | | [56] |
| colposcopy, *colp* | \$165 | | [56] |
| colposcopy and biopsy, *biopsy* | \$318 | | [56] |
| genital warts, $cgw_k$ | \$489 | \$489 | [41] |
| CIN1, $ctcin_1$ | \$1554 | | [50] |
| CIN2, $ctcin_2$ | \$3483 | | [50] |
| CIN3/CIS, $ctcin_3$ | \$3483 | | [50] |
| EICC, $ctcc_L$ | \$26,470 | | [50] |
| RLICC, $ctcc_R$ | \$28,330 | | [50] |
| DLICC, $ctcc_D$ | \$45,376 | | [50] |

Table 8: Cost of screening, diagnosis, and treatment

local invasive cervical cancer \$45,376. Treatment of genital warts is assumed to cost \$489 in 2005 dollars [41].

### 5.6 Cost-effectiveness ratio

To compare mutually exclusive vaccination strategies $a$ and $a'$, we calculate the incremental cost-effectiveness ratio [82]

$$\frac{Cost_a - Cost_{a'}}{QALY_a - QALY_{a'}}.$$

## 6 Analysis using the model

### 6.1 Simulations with the baseline estimates of the parameters

Mathematica® (Wolfram Research, Champaign, IL) version 5.2 was used to generate numerical solutions of the model. The NDSolve subroutine in Mathematica is a general numerical differential equations solver. Since the model consists of non-stiff ODEs, the Explicit Runge Kutta methods, with adaptive embedded pairs of 2(1) through 9(8), provide accurate and less expensive solutions [85]. Other methods such as the Predictor-Corrector Adams method, with orders 1 through 12, produced the same results, but took longer to compute the solution.

The following strategy for simulations was followed. First, the baseline parameter estimates were used to solve the model for the pre-vaccination steady-state values of the variables. Second, the pre-vaccination data were used as initial values for the vaccination model and the model was solved for the entire time path of the variables until the system approached the steady state (approximately 100 years). The solution approximates the potential impact of various HPV

This material, provided by the authors as a supplement to Model for Assessing Human Papillomavirus Vaccination Strategies, is not part of Emerging Infectious Diseases contents.

vaccination programs, including routine vaccination of 12-years old individuals. Finally, once the solution is obtained the results can be presented for various outcomes in many different formats.

### 6.2 Model validation

The validity of a complex model like this cannot be established directly. Instead, its face validity may be judged by how reasonable model assumptions are [34, 81]. In the process of building this model, we comprehensively reviewed previous relevant models and consulted experts on the natural history of HPV infection and HPV-related diseases. A comprehensive review of the literature was conducted to identify studies to inform model inputs. To facilitate independent review of the model and the ability to replicate its results, all model equations and inputs are made available. All model equations and inputs are programmed in Mathematica$^{TM}$ (Wolfram Research, Champaign, IL). A series of tests were performed to debug and establish the technical accuracy of the Mathematica programs. For example, the sum of the number of individuals of a given gender, age, and sexual activity group in each compartment is verified to be equal to the total number of people $N_{kli}$ at each point in time (see section 3.9 on balancing population). Finally, the predictive validity of the model was evaluated by looking at age-specific HPV prevalence, CIN, genital warts, and cervical cancer incidence rates predicted by the model and comparing them with those reported in the literature [29, 47, 73, 74, 75, 41, 45]. The model predictions were well within the range of values found in the literature. For example, the predicted HPV 16/18 attributable cervical cancer incidence curve in the absence of screening had a shape and magnitude at peak (55.9 per 100,000 women years for age 50–54) similar to that estimated for unscreened populations [33, 58].

# References

[1] Abma, J.C., Sonenstein, F.L., 2001. Sexual activity and contraceptive practices among teenagers in the United States, 1988 and 1995. National Center for Health Statistics. Vital Health Stat. 23(21), 1–79.

[2] IARC Working Group on the Evaluation of Carcinogenic Risks to Humans, 1995. IARC monographs on the evaluation of carcinogenic risks to humans. Vol. 64. Human papillomaviruses. Lyons, France: International Agency for Research on Cancer.

[3] Aoyama, C., Peters, J., Senadheera, S., et al., 1998. Uterine cervical dysplasia and cancer: identification of c-myc status by quantitative polymerase chain reaction. Diagn. Mol. Pathol. 7, 324–330.

[4] Andersen, B., 1996. Stress and quality of life following cervical cancer. J. Natl. Cancer Inst. 21, 65–70.

This material, provided by the authors as a supplement to Model for Assessing Human Papillomavirus Vaccination Strategies, is not part of Emerging Infectious Diseases contents.

[5] Barnabas, R. V., Garnett, G. P., 2004. The potential public health impact of vaccines against human papillomavirus. The Clinical Handbook of Human Papillomavirus. W. Prendiville and P. Davies. Lancaster, UK, Parthenon Publishing/Parthenon Medical Communications.

[6] Barnabas, R.V., Laukkanen, P., Koskela, P., et al., 2006. Epidemiology of HPV 16 and cervical cancer in Finland and the potential impact of vaccination: mathematical modelling analyses. PLOS Medicine 3, 1–9. (www.plosmedicine.org).

[7] Bigras, G., de Marval, F., 2005. The probability for a Pap test to be abnormal is directly proportional to HPV viral load: results from a Swiss study comparing HPV testing and liquid-based cytology to detect cervical cancer precursors in 13,842 women. Br. J. Cancer 93, 575–581.

[8] Bosch, F.X., de Sanjose, S., 2003. Chapter 1: Human papillomavirus and cervical cancer-burden and assessment of causality. J. Natl. Cancer Inst. Monogr. 31, 3–13.

[9] Castle, P.E., Schiffman, M., Bratti, M.C., Hildesheim, A., Herrero, R., et al., 2004. A population-based study of vaginal human papillomavirus infection in hysterectomized women. J. Infect. Dis. 190, 458–67.

[10] Center on the Evaluation of Value and Risk in Health. The cost-effectiveness analysis registry [Internet]. (Boston), Tufts-New England Medical Center, ICRHPS. Available from: <http://www.tufts-nemc.org/cearegistry/> (Accessed March 13, 2006).

[11] Centers for Disease Control and Prevention, 2004. Prevention of genital human papillomavirus infection. Report to Congress, Washington DC, January.

[12] Chesson, H.W., Blandford, J.M., Gift, T.L., et al., 2004. The estimated direct medical cost of sexually transmitted diseases among American youth, 2000. Perspect. Sex. Reprod. Health 36, 11–19.

[13] Costa, S., De Simone, P., Venturoli, S., Cricca, M., Zerbini, M.L., et al., 2003. Factors predicting human papillomavirus clearance in cervical intraepithelial neoplasia lesions treated by conization. Gynecol. Oncol. 90, 358–65.

[14] Coste, J., Cochand-Priollet, B., De Cremoux, P., et al., 2003. Cross sectional study of conventional cervical smear, monolayer cytology, and human papillomavirus DNA testing for cervical cancer screening. BMJ 326, 733.

[15] Cruickshank, M.E., Sharp, L., Chambers, G., et al., 2002. Persistent infection with human papillomavirus following the successful treatment of high grade cervical intraepithelial neoplasia. BJOG 109, 579–581.

This material, provided by the authors as a supplement to Model for Assessing Human Papillomavirus Vaccination Strategies,
is not part of Emerging Infectious Diseases contents.

[16] De Aloysio, D., Miliffi, L., Iannicelli, T., et al., 1994. Intramuscular interferon-beta treatment of cervical intraepithelial neoplasia II associated with human papillomavirus infection. Acta. Obstet. Gynecol. Scand. 73, 420–424.

[17] Eddy, D.M., 1980. Screening for cancer: theory, analysis, and design. Prentice-Hall, Englewood Cliffs, New Jersey.

[18] Eddy, D.M., 1990 Screening for cervical cancer. Ann. Intern. Med. 113, 214–226.

[19] Elbasha, E.H., Galvani, A.P., 2005. Vaccination against multiple HPV types. Math. Biosci. 197, 88–117.

[20] Evans, M.F., Mount, S.L., Beatty, B.G., et al., 2002. Biotinyl-tyramide-based in situ hybridization signal patterns distinguish human papillomavirus type and grade of cervical intraepithelial neoplasia. Mod. Pathol. 15, 1339–1347.

[21] Fahs, M.C., Mandelblatt, J., Schechter, C., Muller, C., 1992. Cost effectiveness of cervical cancer screening for the elderly. Ann. Intern. Med. 117, 520–527.

[22] Flannelly, G., Langhan, H., Jandial, L., Mana, E., Campbell, M., Kitchener, H., 1997. A study of treatment failures following large loop excision of the transformation zone for the treatment of cervical intraepithelial neoplasia. Br. J. Obstet. Gynaecol. 104, 718–22.

[23] Fryback, D., Dasbach, E., Klein, R., et al., 1993. Initial catalog of health-state quality factors. Med. Decis. Making 13, 89–102.

[24] Garnet, G.P., Anderson, R.M., 1993. Factors controlling the spread of HIV in heterosexual communities in developing countries: patterns of mixing between age and sexual activity classes. Phil. Trans. R. Soc. Lond. B. 342, 137–159.

[25] Garnett, G.P., Anderson, R.M., 1994. Balancing sexual partnerships in age and activity stratified model of HIV transmission in heterosexual populations. IMA J. Math. Appl. Med. Biol. 11, 161–192.

[26] Garnett, G.P., Hughes, J.P., Anderson, R.M., Stoner, B.P., Aral, S.O., Whittington, W.L., Handsfield, H.H., Holmes, K.K., 1996. The determination of the sexual mixing pattern of patients attending STD and other clinics in Seattle, USA, by contact tracing. Sex. Transm. Dis. 23, 248–257.

[27] Garnett, G.P., Anderson, R.M., 1993. Contact tracing and the estimation of sexual mixing patterns: The epidemiology of gonoccal infections. Sex. Transm. Dis. 20,181–191.

[28] Garnett, G.P., Waddell, H., 2000. Public health paradoxes and the epidemiological impact of an HPV vaccine. J. Clinical Virology 19, 101–111.

[29] Giuliano, A.R., Harris, R., Sedjo, R.L., et al., 2002. Incidence, prevalence, and clearance of type-specific human papillomavirus infections: The Young Women's Health Study. J. Infect. Dis. 186, 462–469.

[30] Gold, M., Franks, P., McCoy, K., Fryback, D., 1998. Toward consistency in cost-utilities analysis. Med. care 36, 778–792.

[31] Goldie, S.J., Grima, D., Kohli, M., Wright, T.C., Weinstein, M.C., Franco, E., 2003. A comprehensive natural history model of human papillomavirus (HPV) infection and cervical cancer: Potential impact of and HPV 16/18 Vaccine. Int. J. Cancer 106, 896–904.

[32] Goldie, S.J., Kohli, M., Grima, D., Weinstein, M.C., Wright, T.C., Bosch, F.X., Franco, E., 2004. Projected Clinical Benefits and Cost-Effectiveness of a Human Papllomavirus 16/18 Vaccine. J. Natl. Cancer Inst. 96, 604–615.

[33] Gustafsson, L., Ponten, J., Bergstrom, R., Adami, H.O., 1997. International incidence rates of invasive cervical cancer before cytological screening. Int. J. Cancer 71, 159–65.

[34] Hammerschmidt, T., Goertz, A., Wagenpfeil, S., et al., 2003. Validation of Health Economic Models. The Example of EVITA. Value Health 6, 551–559.

[35] Harper, D., Franco, E., Wheeler, C., Ferris, D., Jenkins, D., et al., 2004. Efficacy of a bivalent L1 virus-like particle vaccine in prevention of infection with human papillomavirus types 16 and 18 in young women: a randomised controlled trial. Lancet 364, 1757–1765.

[36] Hethcote, H., 1997. An age-structured model of pertussis transmission. Math. Biosci. 145, 89–136.

[37] Ho, G.Y.F., Burk, R.D., Klein, S., et al., 1995. Persistent genital human papillomavirus infection as a risk factor for persistent cervical dysplasia. J. Natl. Cancer Inst. 87, 1365–1371.

[38] Hoyer, H., Scheungraber, C., Kuehne-Heid, R., et al., 2005. Cumulative 5-year diagnoses of CIN2, CIN3 or cervical cancer after concurrent high-risk HPV and cytology testing in a primary screening setting. Int. J. Cancer 116, 136–143.

[39] Hughes, J.P., Garnett, G.P., Koutsky, L.A., 2002. The theoretical population level impact of a prophylactic human papilloma virus vaccine. Epidemiology 13, 631–639.

This material, provided by the authors as a supplement to Model for Assessing Human Papillomavirus Vaccination Strategies, is not part of Emerging Infectious Diseases contents.

[40] Isacson, C., Kessis, T.D., Hedrick, L., et al., 1996. Both cell proliferation and apoptosis increase with lesion grade in cervical neoplasia but do not correlate with human papillomavirus type. Cancer Res. 56, 669–674.

[41] Insinga, R.P., Dasbach, E.J., Myers, E.R., 2003. The health and economic burden of genital warts in a set of private U.S. Health Plans. Clin. Infect. Dis. 36, 1397–1403.

[42] Insinga, R.P., 2006. The natural history of low-grade cervical intraepithelial neoplasia. Manuscript in preparation.

[43] Insinga, R.P., 2006. The natural history of cervical HPV 6/11 infection. 2006. Manuscript in preparation.

[44] Insinga, R.P., Glass, A.G., Rush, B.B., 2004. Pap screening in a U.S. health plan. Cancer Epidemiol. Biomarkers Prev. 13, 355–360.

[45] Insinga, R.P., Glass, A.G., Rush, B.B., 2004. Diagnoses and outcomes in cervical cancer screening: a population-based study. Am. J. Obstet. Gynecol. 191, 105–113.

[46] Insinga, R.P., Dasbach, E.J., Elbasha, E.H., 2006. Epidemiologic natural history and clinical outcomes of human papillomavirus (HPV) disease: a critical review of the literature in the development of an HPV dynamic transmission model. Manuscript in preparation.

[47] Jacobs, M.V., Walboomers, J.M., Snijders, P.J., Voorhorst, F.J., Verheijen, R.H., Fransen-Daalmeijer, N., Meijer, C.J., 2000. Distribution of 37 mucosotropic HPV types in women with cytologically normal cervical smears: the age-related patterns for high-risk and low-risk types. Int. J. Cancer 87, 221–227.

[48] Kataja, V., Syrjanen, K., Mantyjarvi, R., et al., 1989. Prospective follow-up of cervical HPV infections: life table analysis of histopathological, cytological and colposcopic data. Eur. J. Epidemiol. 5, 1–7.

[49] Keshavarz, H., Hillis, S.D., Kieke, B.A., et al., 2002. Hysterectomy surveillance-United States, 1994-1999. MMWR CDC Surveill. Summ. 51, 1–8.

[50] Kim, J., Wright, T., Goldie, S., 2002. Cost-effectiveness of alternative triage strategies for atypical squamous cells of undetermined significance. JAMA 287, 2382–90.

[51] Kochanek, K.D., Murphy, S.L., Anderson, R.N., Scott, C., 2004. Deaths: Final data for 2002. Natl. Vital. Stat. Rep. 53.

[52] Koutsky, L. A., Ault, K. A., Wheeler, C. M., Brown, D. R., Barr, E., et al., 2002. A controlled trial of a human papillomavirus type 16 vaccine. N. Engl. J. Med. 347, 1645–1651.

This material, provided by the authors as a supplement to Model for Assessing Human Papillomavirus Vaccination Strategies, is not part of Emerging Infectious Diseases contents.

[53] Kulasingam, S.L., Myers, E.R., 2003. Potential health and economic impact of adding a human papillomavirus vaccine to screening programs. JAMA 290, 781–789.

[54] Kulasingam, S., Harper, D., Tosteson, A., Myers, E., 2002. Impact of quality-of-life assumptions on cost-effectiveness of cervical cancer screening. 20th International Papillomavirus Conference, Paris, France, October (abstract 121).

[55] Lauman, E., Gagnon, J., Michael, R., Michaels, S., 1994. The social organization of sexuality. University of Chicago Press, Chicago, IL.

[56] Medstat, 2001. MarketScan® database, Thomson Medstat. Ann Arbor, MI.

[57] Matsumoto, K., Yasugi, T., Oki, A., et al., 2006. IgG antibodies to HPV16, 52, 58 and 6 L1-capsids and spontaneous regression of cervical intraepithelial neoplasia. Cancer Lett. 231, 309–313.

[58] McCrory, D., Mather, D., Bastain, L., Datta, S., Hasselblad, V., Hickey, J., Myers, E., Nanda, K., 1999. Evaluation of cervical cytology. Evidence Report/Technology Assessment No.5 (Prepared by Duke University under Contract No. 290-97-0014). AHCPR Publication No. 99-E010. Rockville, MD, Agency for Health Care Policy and Research, February. Available: http://www.ahrq.gov/clinic/epcsums/ cervsumm.htm.

[59] McIntyre, J.A., Leeson, P.A., 2006. Gardasil™: anti-papillomavirus vaccine. Drugs Future 3, 97–100.

[60] Mitchell, M.F., Schottenfeld, D., Tortolero Luna, G., Cantor, S.B., Richards Kortum, R., 1998. Colposcopy for the diagnosis of squamous intraepithelial lesions: a meta analysis. Obstet. Gynecol. 91, 626–31.

[61] Myers, E., McCrory, D., Nanda, K., Bastian, L., Matchar, D., 2000. Mathematical model for the natural history of human papillomavirus infection and cervical carcinogenesis. Am. J. Epidemiol. 151, 1158–71.

[62] Myers, E., Green, S., Lipkus, I., 2004. Pateint preferences for health states related to HPV infection: visual analogue scales vs. time trade-off elicitation. Proceedings of the 21st International Papillomavirus Conference, 390.2, Mexico City, Mexico.

[63] Michael, R., Gagnon, J., Lauman, E., Kolata, G., 1994. Sex in America. Little, Brown & Co, Inc., New York, NY.

[64] Michael, R., Wadsworth, J., Feinleib, J., Johnson, A., Lauman, E., Wellings, K., 1998. Private sexual behavior, public opinion, and public health policy related to sexualy transmitted diseases: a US-British comparison. Am. J. Public Health 88, 749–754.

This material, provided by the authors as a supplement to Model for Assessing Human Papillomavirus Vaccination Strategies, is not part of Emerging Infectious Diseases contents.

[65] Nobbenhuis, M.A., Walboomers, J.M., Helmerhorst, T.J., et al., 1999. Relation of human papillomavirus status to cervical lesions and consequences for cervical-cancer screening: a prospective study. Lancet 354, 20–25.

[66] Parkin, D.M., Bray, F., Ferlay, J., Pisani, P., 2005. Global cancer statistics. CA Cancer J. Clin. 55, 74–108.

[67] Peyton, C., Gravitt, P., Hunt, W., Hundley, R., Zhao, M., Apple, R.J., Wheeler, C.M., 2001. Determinants of genital human papillomavirus detection in a U.S. population. J. Infect. Dis. 183, 1554–1564.

[68] Quade, B.J., Park, J.J., Crum, C.P., et al., 1998. In vivo cyclin E expression as a marker for early cervical neoplasia. Mod Pathol 11, 1238–1246.

[69] Ries, L., Eisner, M., Kosary, C., et al., 2005. SEER cancer statistics review, 1975–2002. Bethesda, MD, National Cancer Institute, http://seer.cancer.gov/csr/1975_2002/.

[70] Saint, M., Gildengorin, G., Sawaya, G. F., 2005. Current cervical neoplasia screening practices of obstetrician/gynecologists in the US. Am. J. Obstet. Gynecol. 192, 414–21.

[71] Sanders, G.D., Taira, A.V., 2003. Cost Effectiveness of a Potential Vaccine for Human Papillomavirus. Emerg. Infect. Dis. 9, 37–48.

[72] Sastre-Garau, X., Cartier, I., Jourdan-Da Silva, N., et al., 2004. Regression of low-grade cervical intraepithelial neoplasia in patients with HLA-DRB1*13 genotype. Obstet. Gynecol. 104, 751–755.

[73] Sellors, J., Mahony, J., Kaczorowski, J., Lytwyn, A., Bangura, H., Lorincz, A., et al., 2000. Prevalence and predictors of human papillomavirus infection in women in Ontario, Canada. CMAJ 163, 503–508.

[74] Sellors, J., Kaczorowski, T., Kaczorowski, J., Mahony, J., Lytwyn, A., Chong, S., et al., 2002. Prevalence of infection with carcinogenic human papillomavirus among older women. CMAJ 167, 871–872.

[75] Surveillance, Epidemiology, and End Results (SEER) Program (www.seer.cancer.gov) SEER*Stat Database: Survival - SEER 9 Regs Public-Use, Nov 2004 Sub (1973–2002), National Cancer Institute, DCCPS, Surveillance Research Program, Cancer Statistics Branch, released April 2005, based on the November 2004 submission.

[76] Stratton, K., Durch, J., Lawerence, R., eds., 2000. Committee to Study Priorities for Vaccine Development. Institute of Medicine. Vaccines for the 21st century: A tool for decisionmaking. Appendix 11. Human Papillomavirus pp 213–222. Washington DC: National Academy Press, http://www.iom.edu/report.asp?id=5648.

This material, provided by the authors as a supplement to Model for Assessing Human Papillomavirus Vaccination Strategies, is not part of Emerging Infectious Diseases contents.

[77] US Bureau of Labor Statistics, 2002. Statistical abstracts of the United States: Consumer price index. National Center for Health Statistics, Washington, DC.

[78] Villa, L.L., Costa, R.L.R., Petta, C.A., Andrade, R.P., Ault, K. A., et al., 2005. Prophylactic quadrivalent human papillomavirus (types 6, 11, 16, and 18) L1 virus-like particle vaccine in young women: a randomised double-blind placebo-conrolled multicentre phase II efficacy trial. Lancet Oncol. 6, 271–78.

[79] Wallin, K.-L., Wiklund, F., Ångström, T., et al.,1999. Type-specific persistence of human papillomavirus DNA before the development of invasive cervical cancer. N. Engl. J. Med. 341, 1633–1638.

[80] Westergaard, L., Norgaard, M., 1981. Severe cervical dysplasia. Control by biopsies or primary conization? A comparative study. Acta Obstet. Gynecol. Scand. 60, 549–554.

[81] Weinstein, M.C., O'Brien, B., Hornberger, J., Jackson, J., Johannesson, M., McCabe, C., Luce, B.R., 2003. Principles of good decision analytic modeling. Value Health 6, 9–17.

[82] Weinstein, M., 1996. From cost-effectiveness ratios to resource allocation: Where to draw the line? In Valuing health care: costs, benefits, and effectiveness of pharmaceuticals and other medical technologies. ed., F Sloan, pp. 77–97. New York: Cambridge University Press.

[83] Wenzel, L., DeAlba, I., Habbal, R., Kluhsman, B.C., Fairclough, D., et al., 2005. Quality of life in long-term cervical cancer survivors. Gynecol. Oncol. 97, 310–7.

[84] Winer, R.L., Kiviat, N.B., Hughes, J.P., Adam, D.E., Lee, S.K., et al., 2005. Development and duration of human papillomavirus lesions, after initial infection. J. Infect. Dis. 191, 731–8.

[85] Wolfram, S., 2005. The Mathematica book, 5th ed. Wolfram Media, Wolfram Research, Inc., Champaign, IL, USA.
